# Targeted Transgene Expression in Rice Using a Callus Strong Promoter for Selectable Marker Gene Control

**DOI:** 10.3389/fpls.2020.602680

**Published:** 2020-12-11

**Authors:** Jie Zhou, Dongyue Li, Chao Zheng, Rumeng Xu, Ersong Zheng, Yong Yang, Yang Chen, Chulang Yu, Chengqi Yan, Jianping Chen, Xuming Wang

**Affiliations:** ^1^State Key Laboratory for Managing Biotic and Chemical Threats to the Quality and Safety of Agro-Products, Ministry of Agriculture Key Laboratory for Plant Protection and Biotechnology, Zhejiang Provincial Key Laboratory of Plant Virology, Zhejiang Academy of Agricultural Sciences, Hangzhou, China; ^2^College of Plant Protection, Northwest A&F University, Yangling, China; ^3^College of Chemistry and Life Sciences, Zhejiang Normal University, Jinhua, China; ^4^Institute of Plant Virology, Ningbo University, Ningbo, China; ^5^Institute of Biotechnology, Ningbo Academy of Agricultural Sciences, Ningbo, China

**Keywords:** transgenic rice, selectable marker, callus strong promoter, transgene expression, β-glucuronidase

## Abstract

Precise expression of a transgene in the desired manner is important for plant genetic engineering and gene function deciphering, but it is a challenge to obtain specific transgene expression free from the interference of the constitutive promoters used to express the selectable marker gene, such as the Cauliflower mosaic virus (CaMV) 35S promoter. So, the solutions to avoid these inappropriate regulations are largely demanded. In this study, we report the characterization of a callus strong promoter (*CSP1*) in rice and its application for accurate transgene expression. Our results indicate that the high expression of the *CSP1* promoter in the callus enables efficient selection of hygromycin equivalent to that provided by the CaMV 35S promoter, whereas its expression in other tissues is low. To evaluate possible leaky effects, the expression of a β-glucuronidase reporter driven by six specific promoters involving hormone signaling, pathogen response, cell fate determination, and proliferation was observed in transgenic rice plants generated by *CSP1*-mediated selection. Distinct β-glucuronidase expression was found consistently in most of the transgenic lines obtained for each promoter. In addition, we applied these specific marker lines to investigate the root cellular responses to exogenous cytokinin and auxin treatment. The results reveal that the root growth inhibition by cytokinin was differently regulated at high and low concentrations. In summary, we have established the feasibility of using callus-specific promoter-dependent selection to mitigate the transgene misexpression in rice. By enabling efficient transformation, rice plants with reliable transgene expression will be easily acquired for broad applications.

## Introduction

Genetic transformation is a valuable method to regulate agronomically important traits both for molecular breeding and for deciphering gene function (Roy et al., 2000). The expression level and specificity of the introduced transgene are largely dependent on the promoters used in the expression cassettes. Tissue-specific and condition-dependent promoters are highly preferred over constitutive promoters because they provide better gene expression and product accumulation while notably alleviating negative effects on plant growth. Numerous tissue-specific promoters have, therefore, been characterized and widely utilized (Potenza et al., 2004; Biłas et al., 2016). Striking examples include placing bioactive genes under the control of endosperm-specific promoters (*Gt13a* or *Gt1*) to produce plant-made pharmaceuticals (Ning et al., 2008; He et al., 2011) or to accumulate β-carotene in Golden Rice seeds (Paine et al., 2005). The cytokinin dehydrogenase gene (*OsCKX4*) controlled by the root-specific promoter (*RCc3*) can promote root development without shoot growth defects (Gao et al., 2014). Recently, the rice embryogenic initiation gene (*BBM1*) was found to trigger parthenogenesis when it was ectopically expressed under the control of the Arabidopsis egg-cell-specific promoter (*pDD45*), which enabled clonal seeds to be formed through asexual propagation (Khanday et al., 2019).

On the other hand, to enable efficient selection of transgenic plants, strong promoters are frequently used to ensure abundant transcription of the selectable marker genes. The 35S promoter from Cauliflower mosaic virus (CaMV) is one of the commonly used constitutive promoters to achieve this purpose (Franck et al., 1980). However, increasing evidence has shown that the 35S promoter used for selection affects the expression pattern of the transgene, possibly because of interactions with the enhancer sequence in the 35S promoter (Yoo et al., 2005). For example, a tapetum-specific promoter (TA29) was used to conditionally express two ribonuclease genes and induce male sterility in transgenic *Brassica napus* and tobacco plants (Mariani et al., 1990). However, it was observed later that the CaMV 35S or its double-enhancer variant used to express the marker gene caused the leaky expression of the cytotoxic gene, hindering the efficient production of stable male sterile plants (Jagannath et al., 2001). Using a spacer DNA fragment between the tapetum-specific promoter and the CaMV 35S promoter significantly improved the recovery of viable male sterile lines (Jagannath et al., 2001). However, this approach is not widely used because the spacers needed to block the interaction differ for each enhancer/promoter combination and so need to be determined case by case (Gudynaite-Savitch et al., 2009).

Several other strategies have been proposed to prevent unintended interactions between the promoters used for selection and transgene expression (Gudynaite-Savitch et al., 2009; Zhou et al., 2014). Clearly, the simple and practical strategy would be to use an alternative promoter that has no/low misexpression effect. The promoters derived from *Agrobacterium tumefaciens*, such as nopaline synthase (*nos*) (Kim et al., 1993), can reduce the ectopic expression of transgenes in some cases (Jagannath et al., 2001; Yoo et al., 2005; Gudynaite-Savitch et al., 2009), but the interference between the *nos* promoter and the target gene promoter often remains considerable especially when they are placed in head-to-head orientation (Denis et al., 1993; Ponstein et al., 2002; Gudynaite-Savitch et al., 2009; Zhou et al., 2014). Among the plant-derived promoters, the tobacco cryptic promoter (tCUP) has proved to be a promising substitute for selectable marker gene expression. It can maintain the appropriate expression of some seed-specific promoters in Arabidopsis regardless of the T-DNA configuration and the distance between the two promoters (Gudynaite-Savitch et al., 2009). The tCUP-derived promoter (tCUP1) also has a conserved property in rice for distinctive expression of *DR5:GUS* in the root apical meristem (Zhou et al., 2014). Although the tCUP promoter and its enhanced versions are strongly expressed in many plant species and tissues, with activities exceeding that of the 35S promoter (Malik et al., 2002; Tian et al., 2003), its activity proved too weak to drive vigorous growth of resistant calli in rice (Zhou et al., 2013). This prompted us to look for other cis-regulatory sequences to confer a strong expression of the selectable marker gene but with a low leaky effect on the specificity of the transgene.

Because the expression of the selectable marker gene is unnecessary once the transformed plants are acquired, the replacement of a strong constitutive promoter by one that is active only during the selection stage is a better choice to eliminate the interference from selectable markers on the expression of the target transgenes. Embryo differentiated callus is the most preferred explant for efficient and convenient rice transformation, and therefore, callus-specific promoters are largely required for marker gene selection, and some of these have been characterized. The β-glucanase 9 (*Gns9*) gene promoter was found to be active only in rice calli and has been used to express a selectable marker gene and obtain transformed plants without the accumulation of antibiotic-resistant protein in other tissues, especially in rice seeds (Huang et al., 2001). A callus-specific promoter from rice β-cysteine protease (*CP*) gene was also used to drive codon-optimized hygromycin phosphotransferase (*HPT*) gene expression for efficient rice transformation (Wang et al., 2012), but it was less effective to drive the *HPT* gene without optimization. These experiments proved that callus-specific promoters could lead to successful transformation and avoid the dispersal of the antibiotic-resistant protein, but their possible interference with transgene expression was not studied.

In the work described here, we identified a callus strong promoter (*CSP1*) by searching a gene expression microarray database. The *CSP1* promoter activity was investigated in rice by fusion to both the β-glucuronidase (*GUS*) reporter and the *HPT* selectable marker. This resulted in strong GUS expression preferentially in callus and provided equivalent levels of a selection of hygromycin as compared with the 35S promoter. Used as an effective selectable marker promoter in rice, the influence of *CSP1* on the tissue-specific promoters was evaluated using GUS as the reporter gene. GUS expression of six synthetic or native promoters was carefully observed in shoots and roots of transgenic plants, including the quiescent-center-specific promoter *QHB*, the cell cycle protein cyclin B1 promoter *CYCB1*, the auxin-inducible promoter *DR5*, the cytokinin two-component signaling sensor *TCSn*, the promoters of cytokinin responsive A-type response regulator *OsRR6*, and the pathogenesis-related gene *PR1b*. All these promoters were specifically expressed in most of the lines as expected, including the responsiveness to cytokinin and auxin applications, which confirmed the root development change controlled by these hormones. Thus, *CSP1*-mediated selection provides a robust and reliable tool to target transgene expression in a tissue-specific and condition-dependent manner in rice.

## Materials and Methods

### GENEVESTIGATOR Analysis

Use the Anatomy tool from the GENE SEARCH toolset of GENEVESTIGATOR^1^ and chose callus as a target. From the selected Affymetrix Rice Genome Array dataset, 10 distinct genes meeting the criteria were identified (Supplementary Figure 1). Then, using the Anatomy tool from the CONDITION SEARCH toolset to compare the target gene expression level in different tissues, the gene LOC_Os10g14020 was identified to be better than the other nine genes (Supplementary Figure 2). Use the Perturbation tool from the CONDITION SEARCH toolset to analyze the response to various stimuli.

### Vector Construction

To construct the *CSP1:GUS* expression vector, the *CSP1* promoter was amplified with polymerase chain reaction (PCR) primers CSP1-Pr-F1 and CSP1-Pr-R1 from the 5′ upstream regulatory sequence of the gene LOC_Os10g14020. A 1,996-bp PCR fragment before the translation start codon (Supplementary Sequence 1) was cloned into the pMD19-T vector (Takara, Dalian, China) and then digested by *Sac*I and *Kpn*I for insertion into the GUS reporter vector *tCUP1-HPT-T35S-GUS-Tnos* (a3) at the same sites (Zhou et al., 2014). The position of cis-elements in the *CSP1* promoter sequence was predicted by the PlantCARE program (Lescot et al., 2002).

The generation of new GUS reporter vectors relied on *CSP1*-activated *HPT* expression. The *CSP1* promoter was amplified with primers CSP1-Pr-F2 and CSP1-Pr-R2 (attached to *Asc*I) to replace the CaMV 35S promoter in the a1 vector (Zhou et al., 2014). The resulting vector was named *CSP1-HPT-GUS* (Figure 2A and Supplementary Excel 1) and *rCSP1-HPT-GUS* (Supplementary Figure 4A) with forward and reverse *CSP1* insertion, respectively. Meanwhile, the *CSP1* promoter was amplified again by primers CSP1-Pr-FW and CSP1-Pr-RV to replace *tCUP1* by In-Fusion HD Cloning (Clontech Laboratories, Inc., CA, United States) into the b3 vector digested with *Asc*I (Zhou et al., 2014). The resulting vector was named *HPT-CSP1-GUS*.

Six specific promoters were selected for the GUS expression assay in the *CSP1-HPT-GUS* vector. The *DR5:GUS* vector was obtained by replacing the *tCUP1* promoter in the a3-DR5 vector (Zhou et al., 2014) with the *Asc*I-digested *CSP1* promoter.

For the *QHB:GUS* vector, a fragment containing the promoter (−1,495 +821) of the *QHB* gene (LOC_Os01g63510) was amplified from the *QHB-GUS-101.3* vector (Jun et al., 2011) by using primers QHB-Pr-F and QHB-Pr-R. After cloning into the pMD19-T vector, it was released by *Sal*I (from pMD19-T) and *Kpn*I (added to the primer) and inserted to the same sites of the *CSP1-HPT-GUS* vector without in-frame fusion with the GUS reporter gene.

The *CYCB1;1:GUS* vector was constructed as described previously (Chen et al., 2013). A translational fusion of the 2,317-bp fragment upstream of the *OsCYCB1;1* (LOC_Os01g59120) start codon plus a 912-bp fragment of the ORF starting at the ATG start site was amplified with the primers CYCB1;1-Pr-F and CYCB1;1-Pr-R. The 3,229-bp PCR product was re-amplified using primers CYCB1;1-Pr-FW and CYCB1;1-Pr-RV for In-Fusion HD Cloning into the *CSP1-HPT-GUS* vector digested with *Xba*I and *Sal*I.

For the *TCSn:GUS* vector, the DNA sequence of *TCSn1* (Supplementary Sequence 2) was synthesized commercially (Genscript, Nanjing, China) according to the vector sequence of TCSn1:GFP-ER (Zurcher et al., 2013). A *Pst*I-digested fragment with mini 35S promoter and TMVΩ translation enhancer (Supplementary Sequence 3) was inserted to the end of *TCSn1* to form a functional *TCSn* promoter. This was then digested by *Sal*I and *Kpn*I for insertion into the *CSP1-HPT-GUS* vector at the same sites.

For the *RR6:GUS* vector, a 2,811-bp PCR fragment upstream of the translation start codon of the cytokinin type A response regulator *OsRR6* (LOC_Os04g57720) was amplified using the primers RR6-Pr-F and RR6-Pr-R. After being cloned into the pMD19-T vector, it was digested by *Xba*I and *Sal*I and inserted into the *CSP1-HPT-GUS* vector at the same sites.

For the *PR1b:GUS* vector, a 2,579-bp PCR fragment upstream of the translation start codon of the pathogenesis-related gene *PR1b* (LOC_Os01g28450) was amplified using the primers PR1b-Pr-FW and PR1b-Pr-RV for In-Fusion Cloning into the *CSP1-HPT-GUS* vector digested with *Xba*I and *Sal*I.

All PCR primer sequences are listed in Supplementary Table 1. Vectors used for experiments are listed in Supplementary Table 2.

### Plant Material and Transformation

The binary vectors described earlier were introduced separately into the *A. tumefaciens* strain EHA105 by electroporation and transformed to embryogenic calli developed from mature seeds of rice (*Oryza sativa* L. cv. Nipponbare) by the method previously reported (Hiei and Komari, 2008) with modifications; no hygromycin is included in the regeneration medium. For comparison of the two *CSP1*-based *HPT-GUS* vectors with their corresponding 35S versions a1 and c1 (Zhou et al., 2014), transformed calli were used to measure the callus growth activities and leaky GUS expression capacities as previously described (Zhou et al., 2013, 2014). Stable transgenic plants were obtained for tissue-specific GUS expression analysis of each promoter tested.

### β-Glucuronidase Histochemical Staining

Histochemical analysis of GUS activity was performed as described by Jefferson et al. (1987). Transformed calli after the first round of selection (approximately 20 days) were incubated in X-gluc staining solution at 37°C for 16 h. For the *CSP1:GUS* transgenic plants, leaf and root samples were taken from 7-day-old (after germination) seedlings of the T_1_ generation of three independent lines in solution culture. Spikelets before flowering were dissected from T_1_ plants at the boot stage in the field. Seeds from the T_2_ generation were de-husked. All these prepared samples were stained with X-gluc solution at 37°C for 16 h. For the six specific promoters constructed in the *CSP1-HPT-GUS* vector, leaf and root samples from T_0_ transgenic plants and the 7-day-old seedlings of descendent generations were incubated with X-gluc solution at 37°C for 16 h, except that the root samples from *DR5:GUS*, *TCS:GUS*, and *CYCB1;1:GUS* transgenic plants were incubated for 30 min. The staining solution contained 1.0-mM 5-bromo-4-chloro-3-indolyl-β-D-glucuronide in 0.1-M phosphate buffer (pH 7.0) with 10-mM ethylene diamine tetraacetic acid disodium salt, 1-mM potassium ferricyanide, 1-mM potassium ferrocyanide, 20% (v/v) methyl hydrate, and 0.5% (v/v) Triton X-100.

### Microscopic Observation

After GUS staining, photos of callus, leaf segment, floret, and seed were taken through a Nikon SMZ1000 stereomicroscope equipped with a Nikon digital camera DS-Fi1. Samples containing chlorophyll were cleared in 100% ethanol before photography. For root samples, the staining solution was removed with water and infiltrated under vacuum for four to five periods of 30 s (Eppendorf, Concentrator Plus, Mode: D-AQ, 30°C). The water was replaced with enough chloral hydrate/glycerol solution (1.6-g chloral hydrate to 1-ml 20% glycerol) to cover the tissue and cleared for several hours. Cleared samples were mounted in chloral hydrate/glycerol solution under a coverslip and directly viewed with a Nikon Eclipse Ti inverted DIC microscope imaging system. The localization of GUS expression in rice roots was described according to the anatomy model illustrated by Coudert et al. (2010).

### Growth Conditions and Hormone Treatments

Germinated transgenic rice seeds were grown on a plastic net floating in International Rice Research Institute rice culture solution in a growth chamber with 30/25°C (day/night) temperature and 60–70% humidity under a 12-h photoperiod. The rice culture solution was supplemented with 0.5-mM 2-(N-morpholino)ethanesulfonic acid to stabilize the pH at 5.2. For cytokinin treatment, 6-day-old *QHB:GUS*, *CYCB1;1:GUS*, and *TCS:GUS* seedlings (after germination) were moved to fresh culture solution with or without 100-μM kinetin (KT) and treated for 24 h (*TCS:GUS*) or time series of 6, 10, and 24 h (*QHB:GUS* and *CYCB1;1:GUS*). *QHB:GUS* and *CYCB1;1:GUS* seedlings were also grown in culture solution with or without 0.2-μM KT for 1 week. For auxin treatment, 6-day-old *DR5:GUS* seedlings were moved to fresh culture solution with or without 1-μM naphthalene acetic acid (NAA) and treated for 24 h. Leaves or primary roots were sampled from treated and mock seedlings at each time point and stained in 1-mM X-gluc for the times indicated in the figure legend.

### Quantitative Real-Time Polymerase Chain Reaction Analysis

For expression analysis of the *CSP1* gene, total RNAs were exacted from 14-day-old young leaves and roots, flag leaves, young and mature spikelets of the wild-type plants, and resistant calli transformed by *35S-HPT-GUS* (a1). Total RNAs were extracted using Trizol reagent (Invitrogen, Carlsbad, CA, United States). First-strand complementary DNA was synthesized by using the iScript cDNA Synthesis Kit (Bio-Rad, Mississauga, ON, Canada) with 0.5-μg total RNA of each sample according to the manufacturer’s protocol. Quantitative real-time (qRT)-PCR was performed on the LightCycler 480 real-time PCR system (Roche Diagnostics, Basel, Switzerland) with UltraSYBR mixture (CWBIO, Beijing, China). The relative expression levels of messenger RNA (mRNA) were normalized using the rice *Actin 1* gene (LOC_Os11g06390), and fold difference in expression was analyzed by the 2^–Δ^ ^Δ^ ^*CT*^ method (Livak and Schmittgen, 2001). Error bars are standard deviations of three technical replicates for each sample. Three biological replicate samples were analyzed. Significant differences were accepted at *P* < 0.05 by the Student *t*-test.

To compare the activities of the *CSP1* and 35S promoters, independent resistant calli were randomly collected from 25 primary calli in two independent transformations with the vectors of *CSP1-HPT-GUS* and *35S-HPT-GUS* (a1) after 14 days of the second round of selection. Relative expressions of *HPT* and *GUS* were analyzed, as described earlier. All the primers used for qRT-PCR are listed in Supplementary Table 3.

### Southern Blot

Sixteen transgenic T_0_ plants from each transformation of *HPT-35S-GUS* (c1) or *HPT-CSP1-GUS* vector were randomly selected for Southern blot analysis. Rice genomic DNA was extracted from 1-g leaves of transgenic T_0_ plants using the sodium dodecyl sulfate method, according to Doyle and Doyle (1990), with a few modifications. Approximately 3-μg genomic DNA per sample was digested by *Nde*I (NEB, Ipswich, United Kingdom) and hybridized with an *HPT* probe, which was prepared using a random primed digoxigenin-DNA labeling kit (DIG High Prime DNA Labeling and Detection Starter Kit II, Roche Diagnostics, Basel, Switzerland) according to the manufacturer’s protocol. Primers for probe amplification are listed in Supplementary Table 1. The processes of DNA digestion, transfer, and hybridization were as previously reported (Zhou et al., 2013). Southern blot hybridization signals were detected with the chemiluminescence substrate CSPD and visualized by the Amersham Imager 600 (GE Healthcare Life Sciences, Marlborough, MA, United States). Copy number was estimated for each line by Image J software.

## Results

### Identification of a Gene Dominantly Expressed in Rice Callus

**FIGURE 1:**
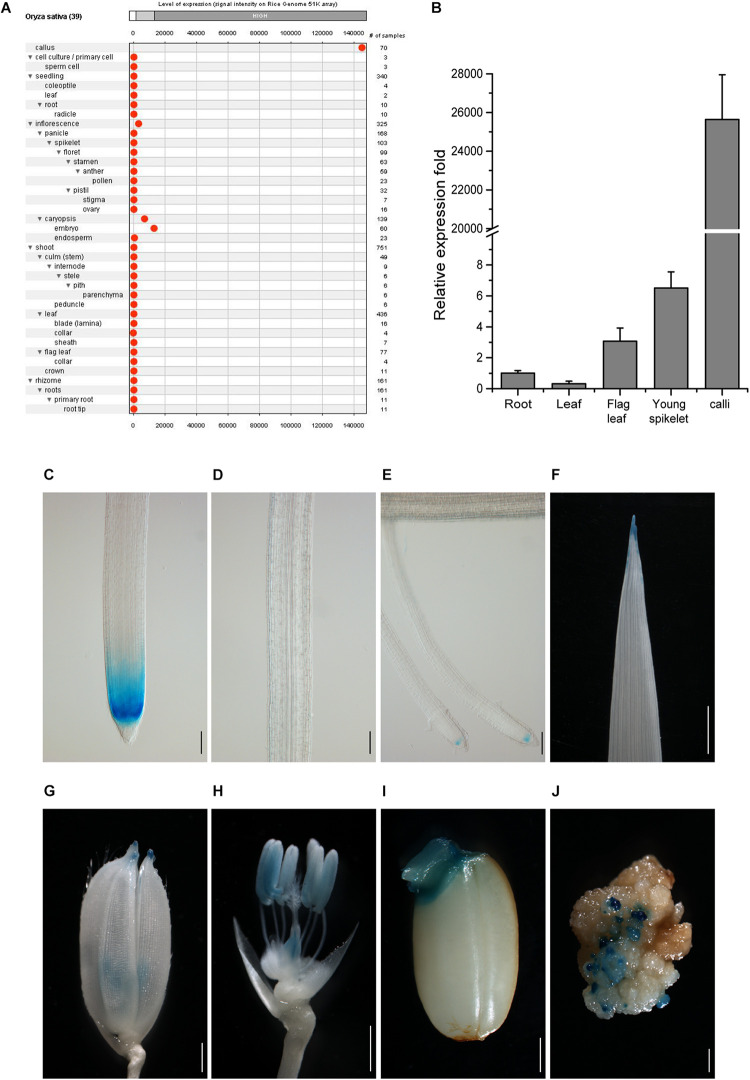
Tissue expression pattern of *CSP1* in rice. **(A)** Tissue expression atlas of *CSP1* gene. Gene expression profile among 39 specific tissues was retrieved from transcriptomes by the Genevestigator program (www.genevestigator.com). **(B)** Real-time RT-PCR analysis of *CSP1* gene expression in different rice tissues. Roots and leaves were collected from 14-day-old solution cultured seedlings. Flag leaf, young spikelets were collected from soil-grown plants at maximum tillering stage. Embryogenic calli were collected from mature seeds after 30 days of inoculation on callus induction medium. *ACT1* was used as the internal reference gene, and relative expression level in each sample is shown as a fold difference compared with roots. Data are means ± SD of three technical replicates as representative of three independent experiments. **(C–E)** Expression pattern of *CSP1:GUS* in roots of 9-day-old seedlings. Three parts of the primary root are shown from distal to proximal end: primary root tip **(C)**, elongation zone **(D)**, differentiation zone with developed lateral roots **(E)**. Scale bars, 100 mm. **(F–J)** Expression of *CSP1:GUS* in leaf tip of 9-day-old seedling **(F)**, tip of lemma and palea **(G)**, anther and ovary **(H)** of mature flower, geminated embryo **(I)**, and resistant calli **(J)**. Scale bars, 1 mm. Images are representatives of three independent GUS-positive transgenic lines.

To search for genes more specifically expressed in callus cells as compared with other tissues, we analyzed rice Affymetrix expression microarray database by GENEVESTIGATOR (Hruz et al., 2008). Better than the other targeted genes (Supplementary Figure 2), the gene LOC_Os10g14020 was strictly expressed in the callus at a high level and only slightly expressed in the inflorescence and embryo (Figure 1A). We thus chose this gene as a candidate for further analysis and named its promoter as *CSP1*.

To confirm the transcriptome data, we first analyzed the gene expression level in different rice tissues by qRT-PCR. Consistent with the gene expression atlas provided by the Anatomy tool of GENEVESTIGATOR, the transcripts were abundant in the embryogenic calli and extremely low in other tissues (Figure 1B), although relatively higher expression was observed in young spikelets than in other tissues except for calli. We also found that the *CSP1* expression level in resistant calli was similar or slightly lower than that in embryogenic calli (Supplementary Figure 3), suggesting that hygromycin treatment did not greatly affect the high *CSP1* activity in the selection stage. To demonstrate the distinct expression pattern of the *CSP1* promoter in rice, the 5′ upstream regulatory sequence before the translation start codon was subject to cis-element analysis. Within a 1,996-bp fragment (−1,896 to +100) (Supplementary Sequence 1), cis-elements related to the responsiveness of light, drought, cold, anaerobic induction, and seed or meristem-specific regulation were predicted (Supplementary Sequence 1 and Supplementary Excel 2). Then, this promoter fragment was cloned into the GUS reporter vector for transformation. After selection, 22 resistant calli were picked randomly for GUS staining, and all of them showed strong GUS expression on the surface of the calli (Figure 1J). After regeneration, 23 PCR-positive, fertile transgenic lines were recovered. Weak or undetectable GUS expressions were found in the T_1_ seedlings of the transgenic lines obtained. Most of the GUS-positive lines had a similar expression pattern to that shown in the representative line (Figures 1C–I), with specific expression in the meristematic zone of primary and developed lateral root (LR) tips (Figures 1C,E) and also in the tips of leaves, lemmas, and paleas (Figures 1F,G). As expected, there was also an expression in the reproductive organs (Figure 1H) and germinated embryo (Figure 1I). These results indicate that the *CSP1* promoter is expressed in a tissue-specific manner in rice and is highly activated in callus.

### *CSP1* Promoter Has Strong Promoter Activity Comparable With Cauliflower Mosaic Virus 35S in Rice Callus

**FIGURE 2:**
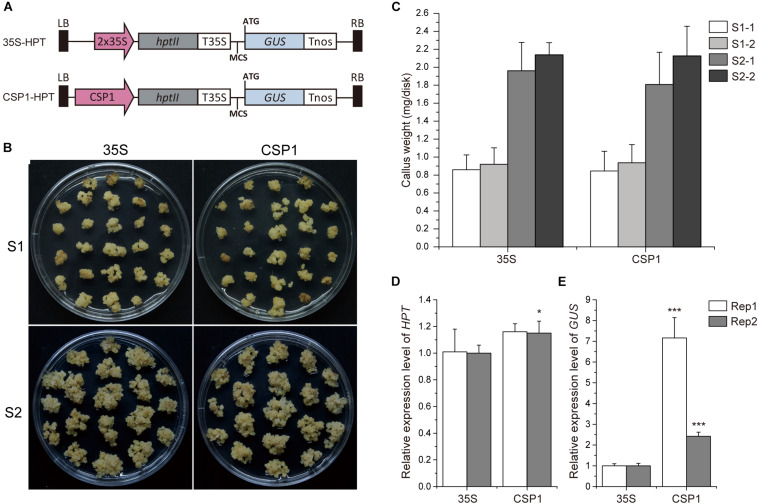
*CSP1* promoter has high transcriptional activity in callus comparable with that of the CaMV 35S promoter. **(A)** Schematic diagram showing the structure of the *HPT-GUS* vectors with CaMV 35S and *CSP1* promoters near the left border (LB) and controlling the expression of *HPT*. A promoterless GUS cassette with multiple cloning sites (MCS) was designed for histochemical analysis. **(B)** Growth of transformed calli 21 days after the onset of first-round selection on 50 mg/L Hyg (S1) and 14 days after the second-round selection on fresh medium with the same Hyg concentration (S2). Left panel, transformed with 35S controlled *HPT-GUS* vector; right panel, transformed with *CSP1* controlled *HPT-GUS* vector. A representative dish of calli from two independent transformations with each vector is shown. **(C)** Hyg-tolerant growth of the calli transformed by the *HPT-GUS* vectors with *HPT* controlled by 35S and *CSP1*, respectively. Weight of fresh calli per dish was measured at the beginning of the first (S1) and second (S2) rounds of selection. Data are mean ± SD (*n* ≥ 5). Experiments were performed twice in two independent transformations (indicated as –1 and –2) with each vector. No significant difference was found at *P* < 0.1 in a Student’s *t*-test. **(D,E)** Relative *HPT* and *GUS* expression levels in secondary resistant calli transformed by 35S and *CSP1*-controlled *HPT-GUS* vectors. Data are mean ± SD of three technical replicates in each of two independent experiments (indicated as Rep 1 and Rep 2). * and *** indicate a significant difference at *P* < 0.05 and 0.001, respectively, in a Student’s *t*-test.

As the *CSP1* promoter is predominantly expressed in the callus, we compared its strength with the constitutive promoter CaMV 35S, which is used for selective marker expression in most binary vectors. We first compared the strength of the *CSP1* and CaMV 35S promoters by their effects on the tolerance of the resistant calli to the selective agent. As shown in Figure 2B, the resistant calli with *HPT* controlled by *CSP1* grew as vigorously as those controlled by CaMV 35S during both the first (S1) and second (S2) rounds of selection with hygromycin. There were no statistical differences in fresh weights at both the start of S1 and S2 between the CaMV 35S and *CSP1*-controlled vectors in two replicated experiments (Figure 2C). At the end of S1, there were approximately 2.3- to 2.4-fold and 2.2- to 2.3-fold increases of weight in calli selected with the CaMV 35S and *CSP1* vectors, respectively (Supplementary Table 4). After the second round of selection, proliferated hygromycin-resistant calli were collected for qRT-PCR analysis. Consistent with the growth results, similar or slightly higher *HPT* mRNA levels were transcribed in resistant calli selected by the *CSP1* promoter compared with those selected by CaMV 35S (Figure 2D). These results demonstrate that the *CSP1* promoter could be used as effectively as the CaMV 35S promoter for embryogenic callus-based transformation in rice.

**FIGURE 3:**
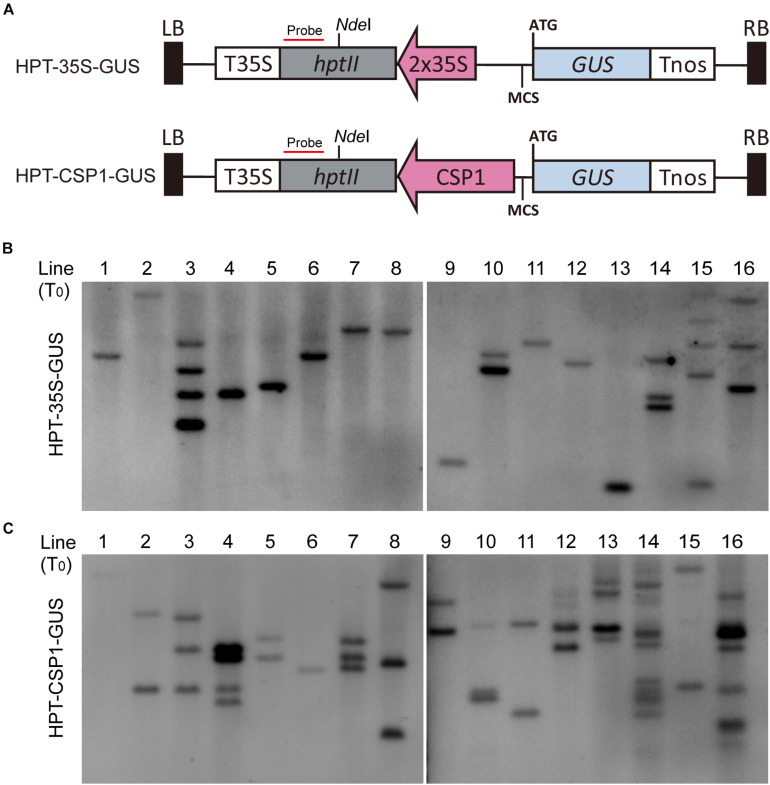
Comparison of copy numbers among transgenic T_0_ plants transformed with *HPT-GUS* vectors controlled by the CaMV 35S and *CSP1* promoters. **(A)** Schematic diagram showing the structure of the *HPT-GUS* vectors with CaMV 35S and *CSP1* promoters in reverse and upstream of the promoterless *GUS* gene and controlling the expression of *HPT*. Probe for Southern blot hybridization was designed from the 3′ end of *HPT* gene after *Nde*I. **(B,C)** Southern blot analysis of integrated T-DNA copy number in 16 independent T_0_ transgenic plants transformed with *HPT-GUS* vectors controlled by 35S **(B)** or *CSP1* **(C)** promoters using the *HPT* probe shown in **(A)**.

Previous work suggested that the activity of the promoter used for selective marker gene expression would affect the inserted T-DNA numbers in the transgenic plants (Zhou et al., 2013). We, therefore, investigated the T-DNA copy numbers integrated into the plants transformed via vectors with *HPT* controlled by CaMV 35S or *CSP1* promoters near the GUS reporter (Figure 3A). Sixteen independent lines randomly selected from each transgenic population were used for Southern blot analysis. As expected, lines transformed with the CaMV 35S vector were found to have low numbers of hybridizing bands varying from 1 to 5, and most of them had only a single band (Figure 3B). Most lines transformed with the *CSP1* vector had multiple bands; numbers varied from 1 to 12, with most having 2 or 3 (Figure 3C). On average, there were approximately 1.7 T-DNA copies integrated into lines transformed with the CaMV 35S vector, but 3.6 copies in those lines transformed with the *CSP1* vector. Because the T-DNA integration pattern in the regenerated plant population represented that in the resistant calli, we previously found that inadequate expression of the selectable marker driven by a weak promoter can lead to preferential survival of cells with multiple copy number to provide sufficient *HPT* expression (Zhou et al., 2013). This, therefore, suggests that the actual activity of a single copy *CSP1* promoter may likely be lower than CaMV 35S in callus, although their overall *HPT* levels were similar (Figure 2D), and that the *CSP1* promoter enables the vigorous, resistant calli growth and high expression of *HPT* is probably achieved by increased copy number.

### *CSP1* Promoter Has Low Level of Reverse Activity

**FIGURE 4:**
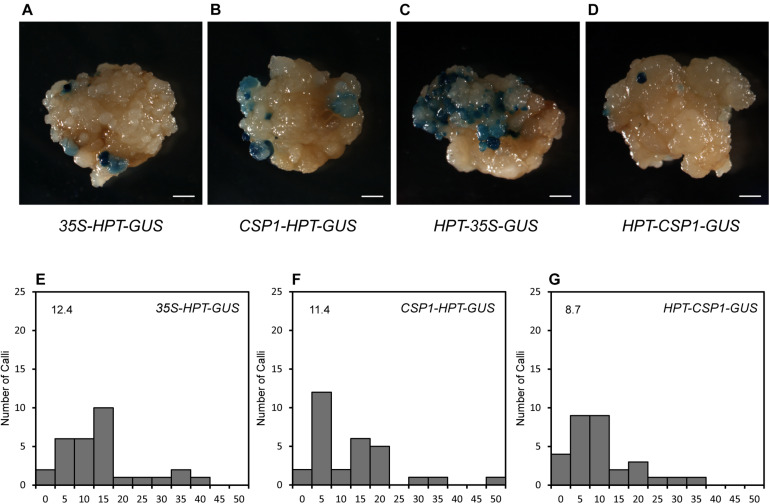
GUS leaky expression in calli transformed by *HPT-GUS* vectors with *HPT* controlled by the CaMV 35S and *CSP1* promoters in two stacking configurations. **(A)** GUS leaky expression in calli transformed by the *35S-HPT-GUS* vector with the CaMV 35S promoter near the LB. **(B)** GUS leaky expression in calli transformed by the *CSP1*-*HPT-GUS* vector with the *CSP1* promoter near the LB. **(C)** GUS leaky expression in calli transformed by the *HPT-35S-GUS* vector with the 35S promoter in reverse and near the GUS gene. **(D)** GUS leaky expression in calli transformed by the *HPT-CSP1-GUS* vector with the *CSP1* promoter in reverse and near the GUS gene. Figures are representative calli from two independent transformations by each vector. Bars: 1 mm. **(E–G)**, Histograms showing the numbers of spots of GUS leaky expression in calli transformed by *35S-HPT-GUS* **(E)**, *CSP1*-*HPT-GUS* **(F)**, and *HPT-CSP1-GUS* **(G)** vectors in two independent experiments, and one of them was shown. A total of 30 transformed calli per vector were selected for GUS staining overnight at 37°C after 2 weeks of selection. *X*-axis indicates the range of numbers of GUS spots per callus from 0 to 50 and divided into 11 intervals: 0, 1–5, 6–10, 11–15, 16–20, 21–25, 26–30, 31–35, 36–40, 41–45, and 46–50. *Y*-axis shows the number of calli that corresponds to each range. Average number of GUS spots per callus derived from each vector is shown on each histogram.

Before using the *CSP1* promoter to select vectors with the GUS gene under specific promoters, we compared the *CSP1* and CaMV 35S promoters for their effects on GUS leaking in transformed calli. Four promoterless GUS vectors in two stacking configurations were transformed for GUS staining (Figures 2A, 3A). GUS leaky expression was determined by histochemical staining of calli after 2 weeks of selection, and numbers of staining spots on the surface of each callus were counted. As reported previously, the inverted 35S promoter upstream of the GUS reporter (*HPT-35S-GUS*) activated the strongest GUS expression in massive cell clusters (Figure 4C), whereas the GUS expression spots were dramatically reduced to a low level when the inverted *CSP1* promoter was at the same position (Figures 4D,G). The low reverse activity of the *CSP1* promoter was further confirmed by its failure to drive effective *HPT* gene expression and allow the transformed calli to survive (Supplementary Figure 4B). When the selectable marker cassette was stacked upstream of the GUS reporter in head to tail orientation, the GUS leaky expression by the separated *CSP1* promoter (*CSP1-HPT-GUS*) was slightly higher than that of the 35S promoter (*35S-HPT-GUS*) in terms of the mRNA level determined by RT-PCR (Figure 2E), but the distribution pattern and average spot number were similar between the two vectors (Figures 4A,B,E,F). Interestingly, among these vectors, the inverted *CSP1* promoter seemed to have the least effect on the leaky expression of GUS, as the resultant staining spots were tiny and weak (Figures 4D,G).

### Development of Specific Promoter Reporter Vectors Using the *CSP1* Promoter to Drive Selectable Marker Expression

**FIGURE 5:**
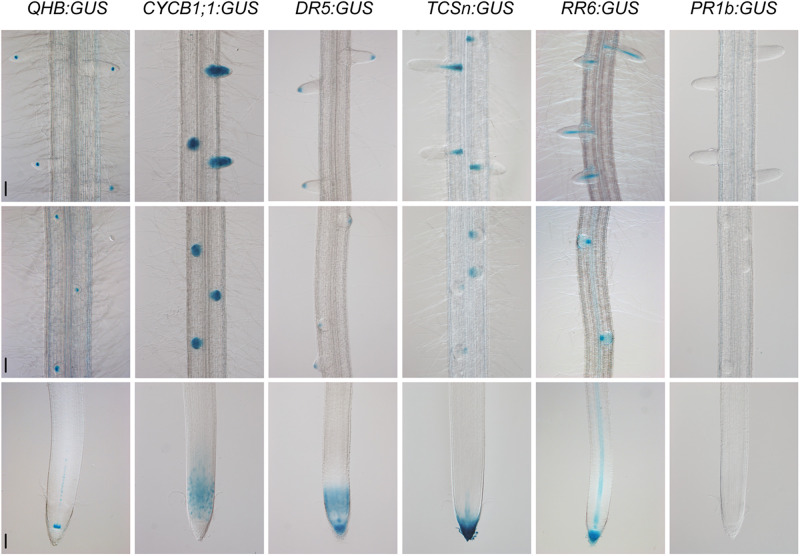
Specific GUS staining patterns in the roots of *QHB/CYCB1;1/DR5/TCSn/RR6/PR1b*:*GUS* transgenic lines selected by vectors based on *CSP1-HPT-MCS-GUS*. Primary roots of 8-day-old seedlings were subjected to GUS staining at 37°C for 22 h (*QHB, TCSn*, and *PR1b*) or 30 min (*CYCB1;1, DR5*, and *RR6*). GUS staining images of the root tips (lower panels), root segments with initiated lateral root primordia (middle panels), and root segments with elongated lateral roots (upper panels) were shown. Bars, 100 μm. Images are representative GUS expression patterns in T_1_ generation of at least three lines.

Because the *CSP1* promoter had similar or even higher potential than the 35S promoter to cause the mis-expression of non-adjacent downstream genes in callus, we investigated the interaction ability of the *CSP1* promoter in other plant tissues. We selected six specific promoters for GUS expression using the vector with *HPT* driven by the *CSP1* promoter (Figure 2A). The GUS expression patterns were first analyzed by histochemical staining of the newly developed adventitious root (AR) tips or leaf segments of the transgenic T_0_ plants. All the vectors except *QHB:GUS* had high GUS-positive staining percentages ranging from 84.6 to 92.0% (Table 1), which indicated that the transformants could be recovered efficiently by using the *CSP1* promoter to drive *HPT* gene expression. Among the 57 GUS-positive lines transformed with *QHB:GUS*, 31 of them had clear and strong GUS staining in the quiescent center (QC) of both AR and LR, similar to the pattern in the T_1_ generation (Figure 5). The remaining 23 lines had weak or invisible GUS expression in the QC of AR but still had distinct expression in the QC of LR. Only three lines had non-specific GUS expression in the region outside the QC, such as the elongation zone. In transgenic plants containing *CYCB1;1:GUS*, all 89 GUS-positive lines had similar expression profiles in the meristematic zone of AR, the whole LR primordia, and emerged LR as shown in the primary root of their T_1_ seedlings (Figure 5). In the 77 GUS-positive transgenic plants containing *DR5:GUS*, four major staining patterns were observed and classified in the T_0_ ARs (Supplementary Table 5). Three of them (types c, d, and h) also occurred when the *tCUP1* vector (a3-DR5) was used in previous experiments (Zhou et al., 2014), and 66 lines showed at least one of these three patterns. Typical *DR5:GUS* expression occurred in the root cap, QC, and protoxylem cells in the AR of T_0_ plants (Supplementary Figure 5). In addition to the same region of the primary root tip, most of the T_1_ seedlings also expressed the *DR5:GUS* in the meristematic zone (Figure 5). For the 45 GUS-positive *TCSn:GUS* transgenic lines, 43 of them had GUS expression confined to the outer layer of the root cap and the stele of AR (Supplementary Figure 6), whereas the other two lines had no expression in these tissues. In the primary roots of T_1_ seedlings, *TCSn*:*GUS* expression was strong in the outer layers of the root cap and gradually attenuated in the columella initials, QC, and stele initials in the meristematic zone (Figure 5). Strong expression was also observed in the vasculature of LR (Figure 5). As for the 75 GUS-positive *RR6:GUS* transgenic lines, 60 of them had GUS staining in the AR root cap and the stele of AR and LR as in the roots of the T_1_ generation (Figure 5). The other 15 lines had expression in the AR root cap or LR only. For the promoter of the pathogenesis-related gene *OsPR1b*, staining patterns in the leaf segments were characterized in the 46 GUS-positive T_0_ lines. Forty-four lines had the same distribution of random GUS spots on the leaf surface, as shown in Supplementary Figure 7. No GUS staining was found in the roots of T_1_ generation seedlings, which served as negative root staining controls (Figure 5). The other two lines had constitutive GUS staining on the leaf surface.

**TABLE 1:** GUS expression patterns observed in roots or leaves of T_0_ transgenic plants.

**Vector**	**GUS+**	**GUS−**	**GUS+/total (%)**	**Expected pattern**	**Unexpected pattern**
*QHB-GUS*	57	41	58.2	54	3
*CYCB1;1-GUS*	89	8	91.8	89	0
*DR5-GUS*	77	14	84.6	66	11
*TCSn-GUS*	45	5	90.0	43	2
*RR6-GUS*	75	13	85.2	72	4
*PR1b-GUS*	46	4	92.0	44	2

*Staining pattern showed by most transformed lines, or consistent to reported pattern was considered as expected pattern, otherwise was considered an unexpected pattern.*

Except for the specific GUS expression in roots, we also observed different leaf expression patterns among these promoter lines. As shown in Supplementary Figure 7, the *QHB:GUS* and *CYCB1;1:GUS* lines had no GUS staining in leaves. The *DR5*:*GUS* line had weak GUS expression in the leaf tip. The *TCSn:GUS* line had GUS staining mainly in the leaf tip and edges of the upper leaf blade, whereas the *RR6:GUS* line had weak GUS staining of the whole leaf.

### Hormone Responsiveness of *QHB*/*CYCB1;1/TCSn/RR6:GUS/DR5:GUS* Reporter Lines Generated From the *CSP1*-Based Expression Vector

**FIGURE 6:**
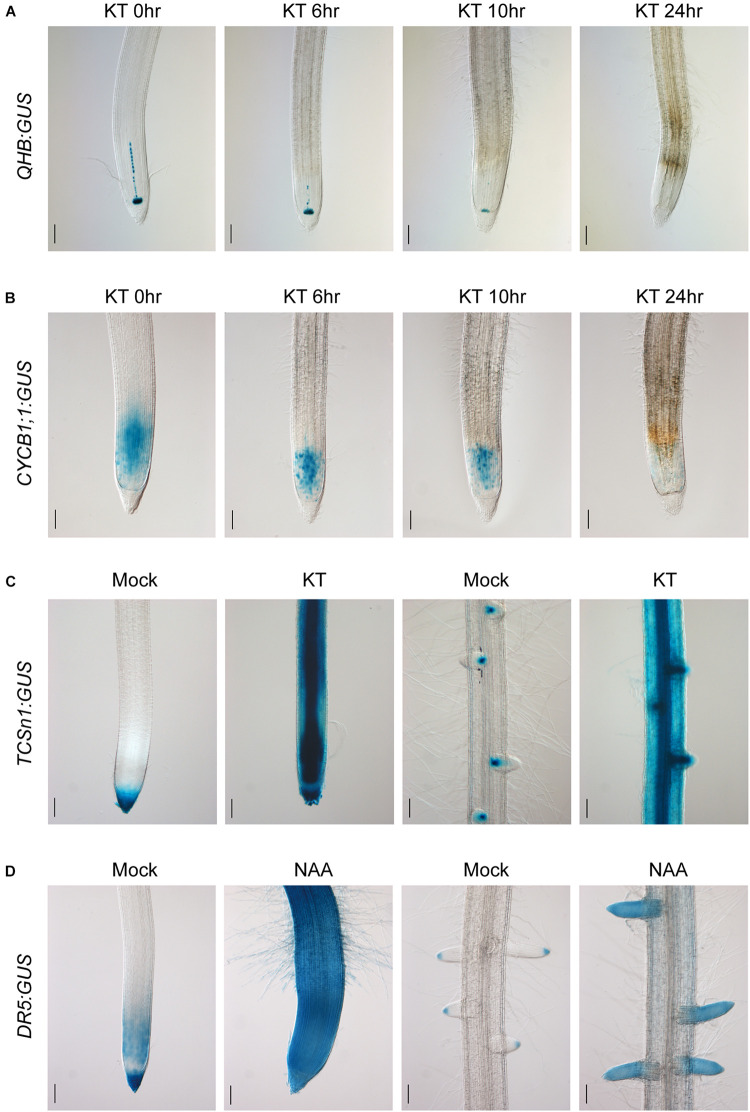
Cytokinin and auxin responsiveness in *QHB/CYCB1;1/TCSn/DR5:GUS* transgenic lines selected by vectors based on *CSP1-HPT-MCS-GUS*. **(A)** Six-day-old *QHB:GUS* transgenic seedlings (T_2_) were subjected to 100-μM KT treatment for 0, 6, 10, and 24 h. Primary root tips were collected at each time point and subjected to GUS staining for 6 h to show the progressively reduced GUS expression in response to prolonged KT treatment. **(B)** Six-day-old *CYCB1;1:GUS* transgenic seedlings (T_2_) were treated with 100-μM KT for the same periods as **(A)**. Primary root tips were collected at each time point and subjected to GUS staining for 1 h. **(C)** Six-day-old *TCSn:GUS* transgenic seedlings (T_2_) were treated with 100-μM KT in fresh culture solution for 24 h or without KT as mock treatment. Primary root tips (left two panels) and a mature root zone with lateral roots (right two panels) were dissected for GUS staining. Strongly induced GUS expressions in the entire root are clearly shown after 7-h staining. **(D)** Six-day-old *DR5:GUS* transgenic seedlings (T_2_) were treated with 1-μM NAA in fresh culture solution for 24 h or without NAA as mock treatment. Primary root tips (left two panels) and a mature root zone with lateral roots (right two panels) were dissected for GUS staining. Increased GUS expression is clearly shown after 30-min staining. Bars, 100 μm. Images are representative GUS expression patterns of at least three lines.

After characterizing the specific expression patterns of the promoters constructed in the *CSP1*-based expression vector, we then examined the hormone responsiveness of the GUS reporter lines of the *QHB*, *CYCB1;1*, *TCSn*, *RR6*, and *DR5* promoters. As shown in Figure 6A, *QHB:GUS* expression in the root stem cells progressively decreased after continued application of 100-μM cytokinin KT for 24 h. Under the same conditions, the staining area of *CYCB1;1:GUS* in the primary root meristem was significantly reduced after treatment for 8 h and was almost completely lost after 24 h (Figure 6B). These results suggested that the high level of external cytokinin treatment may inhibit the root growth by dampening the QC identity and root meristem cell activity.

To assess the sensitivity of *TCSn:GUS* to cytokinin, hydroponically cultured transgenic seedlings were treated with 100-μM KT for 24 h. Compared with the mock treatment, strong GUS expression was found in the entire primary root tip and the mature root region with the most induction in the vascular bundle and LRs (Figure 6C). A similar induction pattern was obtained with *RR6:GUS* under the same condition (Supplementary Figure 8). Meanwhile, the ability of auxin-induced *DR5:GUS* expression was also confirmed by the exogenous application of 1-μM NAA for 24 h. Compared with the restricted expression in the mock treatment, strong and constitutive expression was induced in the whole primary root tip and LRs (Figure 6D), similar to the auxin-responsive pattern of a3-DR5 reported previously. These experiments clearly demonstrated that the *CSP1* promoter used for selectable marker expression did not affect the ability of the *TCSn/RR6/DR5* promoters to respond to hormones.

## Discussion

The GENEVESTIGATOR analysis showed that the gene LOC_Os10g14020 was strongly expressed in the callus and relatively weakly in the inflorescence and embryo (Figure 1A), and it was higher and more specifically expressed in the callus than the reported *Gns9* and *CP* genes. This expression pattern was consistent with the expression profile from the RiceXpro database^2^ (Sato et al., 2011), which showed specific expression of the target gene in ovule and embryo among the tissue/organs encompassing the entire growth of the rice plant except for callus (Supplementary Figure 9A). In line with the transcriptome analysis, qRT-PCR confirmed the overwhelming expression of the target gene in the embryogenic calli, far beyond the levels in other tissues (Figure 1B), and we, therefore, designated its promoter as *CSP1*. *In silico* promoter analysis on PlantCARE shows cis-acting regulatory elements related to the responsiveness of light, drought, and cold and also elements involved in seed or meristem-specific regulation (Supplementary Figure 1 and Supplementary Excel 2), which is supported by GENEVESTIGATOR perturbation analysis, which reveals the induction of *CSP1* by light, high temperature, and dehydration (Supplementary PDF 1). Expression analysis on the RiceXpro database shows that the *CSP1* is stable in response to plant hormone treatment (Supplementary Figure 9B). Matched with the *in silico* analysis, GUS histochemical analysis indicated that the *CSP1* promoter was also expressed in the root meristem, leaf tips, lemma and palea, ovary, anther, and embryo of rice (Figures 1C,I). Although the function of the target gene in callus is not known, promoter deletion analysis would be helpful to isolate and characterize the cis-regulatory elements specific to callus and their role on *CSP1* promoter regulated expression. In addition, *CSP1* is annotated in the National Center for Biotechnology Information as a homolog of Arabidopsis TPD1 (tapetum determinant 1) protein, and so it is very likely to have similar involvement in reproductive organ development (Yang et al., 2003; Huang et al., 2016; Chen et al., 2019).

Cauliflower mosaic virus 35S is the most frequently used promoter in plant biotechnology. It can affect the expression of transgenes located either downstream or upstream (Yoo et al., 2005; Gudynaite-Savitch et al., 2009), possibly via its enhancer regions (Benfey et al., 1990a,b). As shown in this study as well as our previous report (Zhou et al., 2014), substantial GUS leaking occurred when the 35S promoter was placed either in reverse orientation (Figure 4C) or separated by the *HPT* gene (Figure 4A). In contrast to the strong bidirectional activity of the CaMV 35S promoter, the reverse *CSP1* promoter had only the slightest capacity to activate adjacent *GUS* or *HPT* gene expression (Figures 4D,G and Supplementary Figure 4), indicating that the *CSP1* promoter is under tight regulation. With its strict unidirectional expression, *CSP1* could be used as an insulator to preventing inappropriate cross-regulation of its flanking neighborhood. Because only a few plant insulators have so far been identified and shown to be functional (Hily et al., 2009; Singer et al., 2011; Yang et al., 2011), it would be interesting to assess the feasibility of using *CSP1* to block enhancer–promoter communication and to study its impact on upstream gene expression.

In this study, we examined the effect of *CSP1* promoter on its downstream GUS gene expression. First, we compared the leaky expression of the promoterless GUS gene in callus caused by the upstream *CSP1* or 35S promoters (Figure 2A). No big differences were found in terms of the spot size and average spot number per callus (Figures 4A,B,E,F), but two to sevenfold higher GUS gene transcription was caused by the *CSP1* promoter compared with the 35S (Figure 2E). This suggests that the strong activity of *CSP1* also leads to a high mis-expression of non-adjacent downstream genes in the callus. However, when we looked at the GUS expression in leaf or root after the introduction of particular promoters with distinct cellular expression, most of the transgenic plants had the GUS expression expected from the respective promoter (Table 1). Thus, it appears that the potential multi-promoter interactions may be mitigated if the promoter binding factors are not expressed at the same time and space. In the case of the *CSP1* promoter, the brisk transcriptional activity was decreased and confined to specific regions when plants were regenerated from differentiated calli (Figure 1), which greatly reduces the contact opportunity between the factors binding to the *CSP1* and other promoters in the same vector. Interestingly, even in the region where *CSP1* was expressed, e.g., in the root meristematic region (Figures 1C,E), no override of expression was shown on the target promoters (Figure 5). The lines transformed by the same promoter vector differed at most in the varied intensity of GUS expression (Supplementary Figure 6). This could be the result of multiple T-DNA integrations in plants generated by *CSP1*-based selection (Figure 3B), and the number of T-DNA copies may positively affect transgene expression, as we reported previously (Zhou et al., 2013).

Based on this reporter vector, we characterized the GUS expression profiles of six synthetic or native promoters involving hormone signaling, pathogen response, cell fate determination, and proliferation in both roots and leaves (Figure 5 and Supplementary Figure 7). The synthetic auxin-inducible promoter *DR5* is the most widely used sensor to monitor auxin response and distribution (Ulmasov et al., 1997). Previously, we have made a detailed observation of *DR5:GUS* expression in rice roots using vectors where a selectable marker gene was controlled by different promoters (Zhou et al., 2014). Typical patterns were obtained by the *a3-DR5* vector in the root cap, QC, and protoxylem cells in the meristem, quite similar to that observed by *DR5rev*-controlled 3xVenus fluorescent reporters in rice roots (Heisler et al., 2005; Li et al., 2014). For comparison, we examined the expression of *DR5* by the vector similar to a3-DR5, where the *tCUP1* was replaced by *CSP1.* Although different patterns were observed in the T_0_ ARs (Supplementary Table 5), many of them (types c, d, and h) also occurred when the *a3-DR5* vector was used. The classical *DR5:GUS* expression was also found in the AR tip of *CSP1-DR5* T_0_ plants (Supplementary Figure 4), except that GUS expression was confined to the initial xylem cells and was weaker than *a3-DR5*. Overall, these data indicated that the specificity of *DR5* could be retained by the use of the *CSP1* promoter to drive selectable marker gene expression.

Although *DR5* has been regularly used to study auxin, the synthetic two-component signaling sensor (*TCS*) has only been developed relatively recently to study cytokinin signaling output (Muller and Sheen, 2008). Because of some limitations, an improved new version *TCSn* was optimized with superior strength and sensitivity (Zurcher et al., 2013). The function of *TCSn* in correlation with cytokinin has mainly been studied in Arabidopsis involving multiple developmental contexts from embryogenesis to shoot and root formation. It was also conservatively expressed in rice by fusing to a GUS reporter (Tao et al., 2017). Here, we expressed *TCSn:GUS* in rice by the *CSP1*-based vector and analyzed the GUS expression in roots and leaves. In our experiments, *TCSn*-driven GUS was intensively expressed in the outer layers of the primary root cap and less so in the cells at the boundary between the root and root cap, including QC, root cap initials, and immature stele (Figure 5). This result was consistent with the *TCSn* expression in Arabidopsis root apex as well as that reported in rice (Zurcher et al., 2013; Tao et al., 2017) and in close agreement with the high-resolution cytokinin distribution measured in specific types of root cells (Antoniadi et al., 2015). Strong expression was also found in the steles of AR and LR (Figure 5 and Supplementary Figure 6) but not in the LR cap, as shown in the two previous reports (Zurcher et al., 2013; Tao et al., 2017). Besides the synthetic cytokinin sensor *TCSn*, we also expressed the endogenous cytokinin responsive promoter of *RR6*, an A-type response regulator in the rice two-component signaling system (Hirose et al., 2007). Maximum expression of *RR6:GUS* was also found in the root cap and stele tissues but not in the LR cap (Figure 5). Both *TCSn* and *RR6*-mediated GUS expression can be remarkably induced in roots by exogenous application of cytokinin (Figure 6B and Supplementary Figure 8), suggesting that their GUS activities are consistent with the function of cytokinin.

The root apical meristem is the important place where root radial organization begins by ordered cell division and differentiation from initial cells around the QC (Dolan et al., 1993). The QC in Arabidopsis comprises four mitotically inactive cells where the *WUCHEL-RELATED HOMEOBOX 5* (*WOX5*) is specifically expressed (Sarkar et al., 2007). In rice, similar small numbers of QC cells are characterized, but the ortholog of *WOX5*, *QUIESCENT-CENTER-SPECIFIC HOMEOBOX* (*QHB*) is expressed in a broad area including stem cells near the QC (Ni et al., 2014). Here, we showed that *QHB:GUS* constructed in the *CSP1*-based vector had a similar expression level in the center of the root meristem containing the QC cells and its surrounding stem cells (Figure 5 and Supplementary Figure 10). The stability of the QC has been studied under various conditions (Ni et al., 2014). Although root architecture was altered, the cell patterns of the QC were not affected under nutrient deficiency or treatment with auxin and cytokinin, suggesting that QC activity was largely maintained to keep continuous root growth and function in the unfavorable environment. In agreement with that report, we found that expression of *QHB:GUS* in the stem cell niche was not affected by treatment with 0.2-μM KT for 7 days (Supplementary Figure 11B), but the number of cells with high mitotic activity indicated by *CYCB1;1:GUS* (Doerner et al., 1996) was reduced after the same treatment (Supplementary Figure 11C). These results indicated that a low cytokinin level greatly inhibited root growth (Supplementary Figure 11A) by damaging root meristem activity.

After becoming aware of the strong artificial effect of the CaMV 35S promoter on specific promoters constructed in the same vector, most of the subsequent promoter analysis in Arabidopsis and rice have been done in vectors where the *Nos* promoter was used for selectable marker gene expression. The reported promoter patterns we used for comparison were mainly obtained in these vectors. The consistent-specific expression could be obtained in some cases, such as the expression of *QHB* (Kamiya et al., 2003; Ni et al., 2014) and *CYCB1;1* (Chen et al., 2013) was similar to that obtained in this study. The expression of *PR1b* previously analyzed in a *Nos* vector (Yu et al., 2014) was also similar to that expressed in the *CSP1* vector. With *TCSn*, strong expression in the whole LR and root hairs were shown in two previous reports (Zurcher et al., 2013; Tao et al., 2017), but we found no expression in the LR cap and only weak expression in part of the root hairs. Both of the vectors in those earlier reports, pCB302 and pBI101, used the *Nos* promoter to express either Basta or kanamycin-resistant genes for selection (Jefferson et al., 1987; Xiang et al., 1999). The possible interference between the *Nos* promoter and *TCSn* in these tissues needs to be analyzed by further experiments.

## Data Availability Statement

The original contributions presented in the study are included in the article/Supplementary Material, further inquiries can be directed to the corresponding author/s.

## Author Contributions

XW, JZ, and JC conceived the project, designed the experiments, and wrote the manuscript. JZ, DL, and CZ performed the experiments with assistance from EZ, YY, CYu, and YC. JZ, XW, and CYa analyzed the results. All authors contributed to the article and approved the submitted version.

## Conflict of Interest

The authors declare that the research was conducted in the absence of any commercial or financial relationships that could be construed as a potential conflict of interest.

## Footnotes

1 https://genevestigator.com/

2 http://ricexpro.dna.affrc.go.jp/

## Supplementary Material

Supplementary Figure 1 Screen of the callus-specific expressed genes in rice. Ten distinct genes were searched out using the Anatomy tool of GENEVESTIGATOR with callus as target.

Supplementary Figure 2 Tissue expression patterns of the 10 callus-specific candidate genes. Relative expression levels are shown in different tissues and compared among the selected 10 genes using the Anatomy tool of GENEVESTIGATOR.

Supplementary Figure 3 Relative *CSP1* expression levels in untransformed calli and secondary resistant calli transformed by 35S controlled *HPT-GUS* vector. Data are mean ± SD of three technical replicates in each of two independent experiments (indicated as Rep 1 and Rep 2). ^∗^ indicate a significant difference at *P* < 0.05 in a Student’s *t*-test.

Supplementary Figure 4 The reverse *CSP1* promoter did not drive the effective expression of *HPT* gene in callus for transformant selection. Growth of transformed calli 14 days after the second-round selection on fresh medium with 50 mg/L Hyg. Left panel, transformed with 35S controlled *HPT-GUS* vector; right panel, transformed with reverse *CSP1* controlled *HPT-GUS* vector. A representative dish of calli in each transformation is shown.

Supplementary Figure 5 Typical GUS expression patterns in the adventitious roots of *DR5:GUS* T_0_ transgenic lines selected by the *CSP1-HPT-MCS-GUS* vector. Adventitious root tips of three independent lines T_0_-36 (left), T_0_-41 (middle), and T_0_-62 (right) are shown. GUS was mainly expressed in the root cap, quiescent center and protoxylem cells in the root meristem. Bars: 100 μm.

Supplementary Figure 6 GUS expression patterns in the adventitious roots of *TCSn:GUS* T_0_ transgenic lines selected by the *CSP1-HPT-MCS-GUS* vector. Adventitious root tips of two independent lines are shown as representative of weak (T_0_-6) or strong (T_0_-42) GUS expression patterns of the *TCSn* promoter. Similar GUS staining was detected predominantly in the outer layers of root cap and stele. Bars: 100 μm.

Supplementary Figure 7 Specific GUS staining patterns in the leaves of *QHB/CYCB1;1/DR5/TCSn/RR6/PR1b:GUS* transgenic lines selected by vectors based on *CSP1-HPT-MCS-GUS*. GUS expressions of the first fully expended leaf of 8-day-old seedlings were analyzed. Leaf tips (upper panels) and adjacent part near the leaf tips (lower panels) are shown. Bars, 2 μm.

Supplementary Figure 8 Cytokinin responsiveness of *RR6:GUS* transgenic plant transformed by the *CSP1* controlled vector. 8-day-old *RR6:GUS* transgenic seedlings (T_1_) were treated with 100 μM KT in fresh culture solution for 24 h or without KT as mock treatment. Primary root tips (left two panels) and mature root zone with lateral roots (right two panels) were dissected for GUS staining. Strongly induced GUS expressions in the entire root is clearly shown after 7 h staining. Bars, 100 μm.

Supplementary Figure 9 Expression profile of *CSP1* analyzed on the RiceXpro platform (http://ricexpro.dna.affrc.go.jp/). **(A)** The spatial temporal expression profile. **(B)** Expression profile in response to plant hormones in root and shoot.

Supplementary Figure 10 The expression pattern of *QHB* in the root tip of rice. Primary root tip of 7-day-old *QHB:GUS* transgenic seedling (T_1_) was subjected to GUS staining for 6 h and observed under microscope with 20× objective lens after clearing. GUS expression was found in the center of the root meristem containing the QC and its surrounding stem cells. The red arrows indicate the single layer of epidermal cells originating from the stem cells near the QC. Bars: 50 μm.

Supplementary Figure 11 GUS expression activity of *QHB* and *CYCB1;1* in root tip after cytokinin treatment at low level. **(A)** Root growth inhibition of *QHB:GUS* and *CYCB1;1:GUS* transgenic seedlings (T_3_) grown on solution cultures with or without 0.2 μM KT for 7 days. Bars: 2.5 cm. **(B)** *QHB:GUS* expression in the quiescent center of the mock and KT treated roots after GUS staining for 16 h. Bars: 100 μm. **(C)** *CYCB1;1:GUS* expression in the meristematic region of the mock and KT treated roots after GUS staining for 1 h. Bars: 100 μm.

Supplementary Table 1 Primers used for plasmid construction.

Supplementary Table 2 Vectors used in experiments.

Supplementary Table 3 Primers used for quantitative real-time PCR analysis and Southern blot.

Supplementary Table 4 Callus weight at the start of the first (S1) and second (S2) rounds of selection.

Supplementary Table 5 GUS expression patterns observed in roots of *DR5:GUS* T_0_ transgenic plants.

Supplementary Sequence 1 Sequence of the *CSP1* promoter amplified (1996 bp).

Supplementary Sequence 2 Synthesized sequence of *TCSn1* (282 bp).

Supplementary Sequence 3 *Pst*I digested mini35S-TMVΩ fragment for ligation of *TCSn1* (147 bp).

Supplementary Excel 1 Vector sequence of *CSP1-HPT-T35S-MCS-GUS*.

Supplementary Excel 2 Cis-elements analysis of the *CSP1* promoter by PlantCARE.

Supplementary PDF 1 Perturbation analysis of the *CSP1* gene by GENEVESTIGATOR.
