# Supplementary material for: Targeted Transgene Expression in Rice Using a Callus Strong Promoter for Selectable Marker Gene Control
Source: Front Plant Sci. 2020 Dec 11;11:602680. doi: 10.3389/fpls.2020.602680 (PMC7759479; doi:10.3389/fpls.2020.602680)
Supplement: Supplementary Figure 1 — Screen of the callus-specific expressed genes in rice. Ten distinct genes were searched out using the Anatomy tool of GENEVESTIGATOR with callus as target. [file Data_Sheet_2.docx]

Supplementary Material





**Supplementary Figure 1.** Screen of the callus-specific expressed genes in rice. 10 distinct genes were searched out using the Anatomy tool of GENEVESTIGATOR with callus as target.





**Supplementary Figure 2.** Tissue expression patterns of the 10 callus-specific candidate genes. Relative expression levels are shown in different tissues and compared among the selected 10 genes using the Anatomy tool of GENEVESTIGATOR.


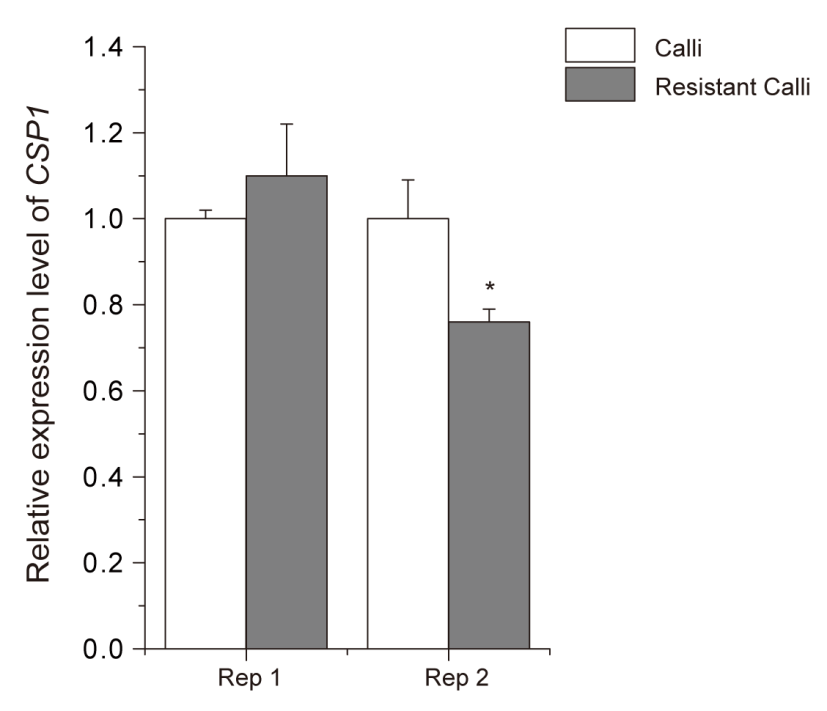


**Supplementary Figure 3.** Relative *CSP1* expression levels in untransformed calli and secondary resistant calli transformed by 35S controlled *HPT-GUS* vector. Data are mean ± SD of three technical replicates in each of two independent experiments (indicated as Rep 1 and Rep 2). * indicate a significant difference at *P<*0.05 in a Student’s *t*-test.


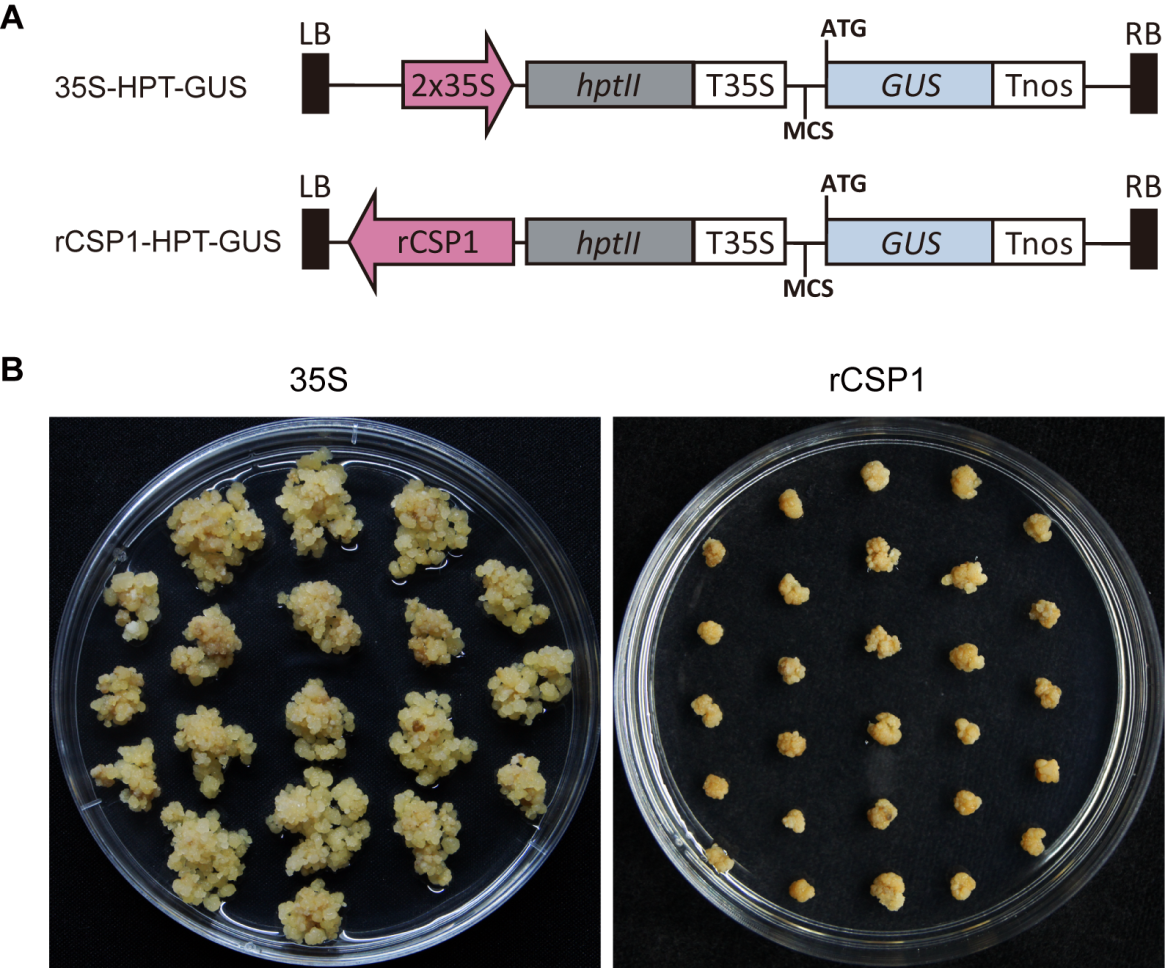


**Supplementary Figure 4.** The reverse *CSP1* promoter did not drive the effective expression of *HPT* gene in callus for transformant selection. (**A**) Schematic diagram showing the structure of the *HPT-GUS* vectors with CaMV 35S (2x35S) and reverse CSP1 (rCSP1) promoters near the left border (LB) and upstream of *hptII* gene. (**B**) Growth of transformed calli 14 days after the second-round selection on fresh medium with 50 mg/L Hyg. Left panel, transformed with 35S controlled *HPT-GUS* vector; right panel, transformed with *rCSP1* controlled *HPT-GUS* vector. A representative dish of calli from two independent transformations with each vector is shown.


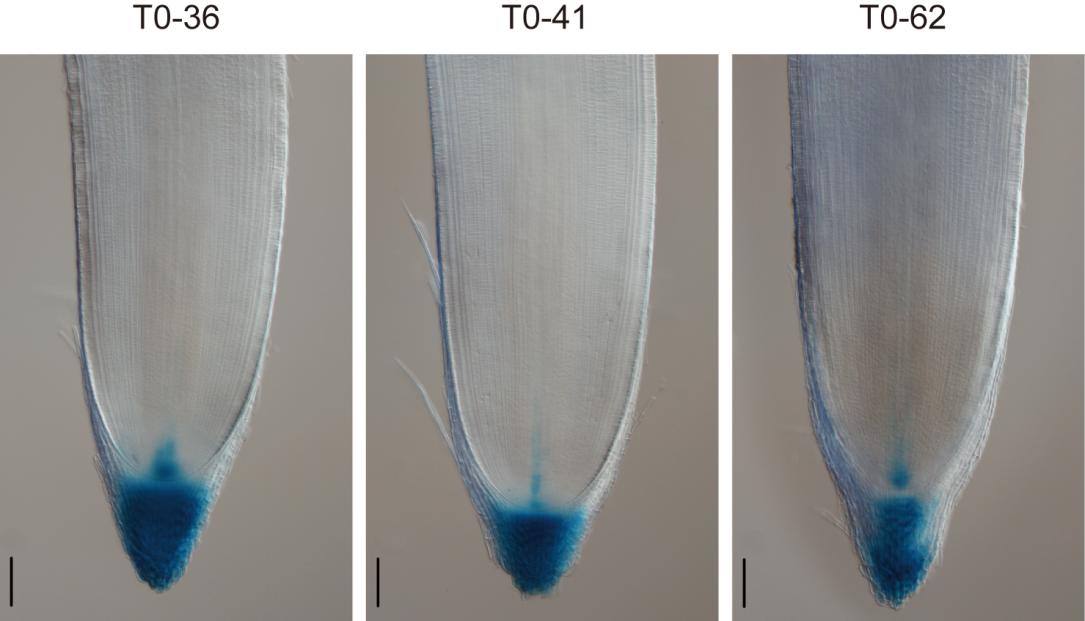


**Supplementary Figure 5.** Typical GUS expression patterns in the adventitious roots of *DR5:GUS* T_0_ transgenic lines selected by the *CSP1-HPT-MCS-GUS* vector. Adventitious root tips of three independent lines T_0_-36 (left), T_0_-41 (middle) and T_0_-62 (right) are shown. GUS was mainly expressed in the root cap, quiescent center and protoxylem cells in the root meristem. Bars: 100 μm.


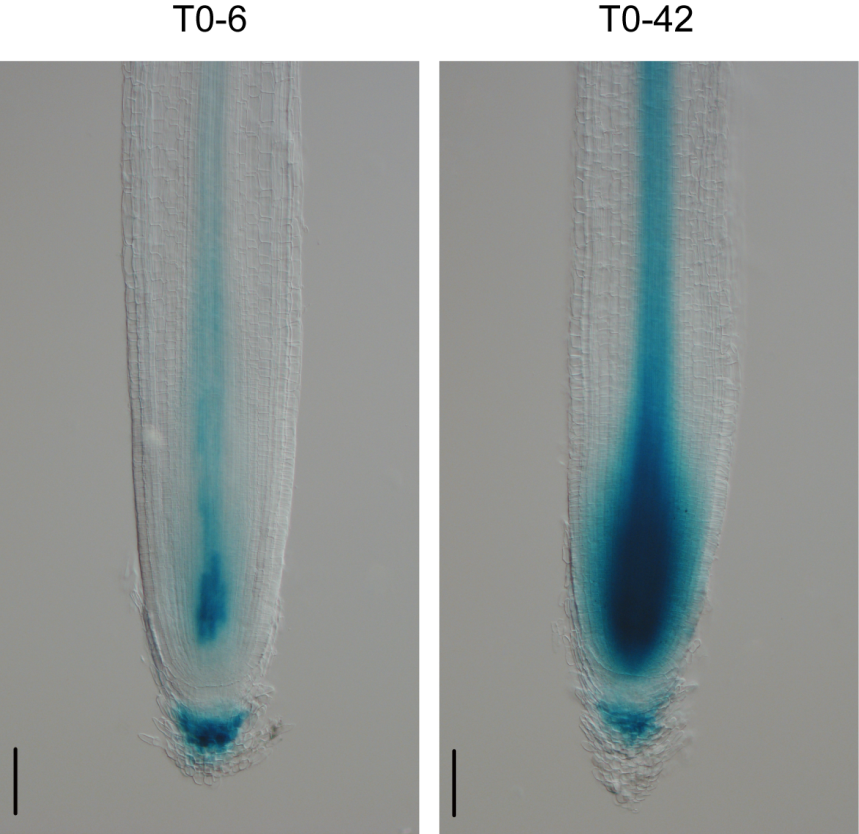


**Supplementary Figure 6.** GUS expression patterns in the adventitious roots of *TCSn:GUS* T_0_ transgenic lines selected by the *CSP1-HPT-MCS-GUS* vector. Adventitious root tips of two independent lines are shown as representative of weak (T_0_-6) or strong (T_0_-42) GUS expression patterns of at least three lines. Similar GUS staining was detected predominantly in the outer layers of root cap and stele. Bars: 100 μm.


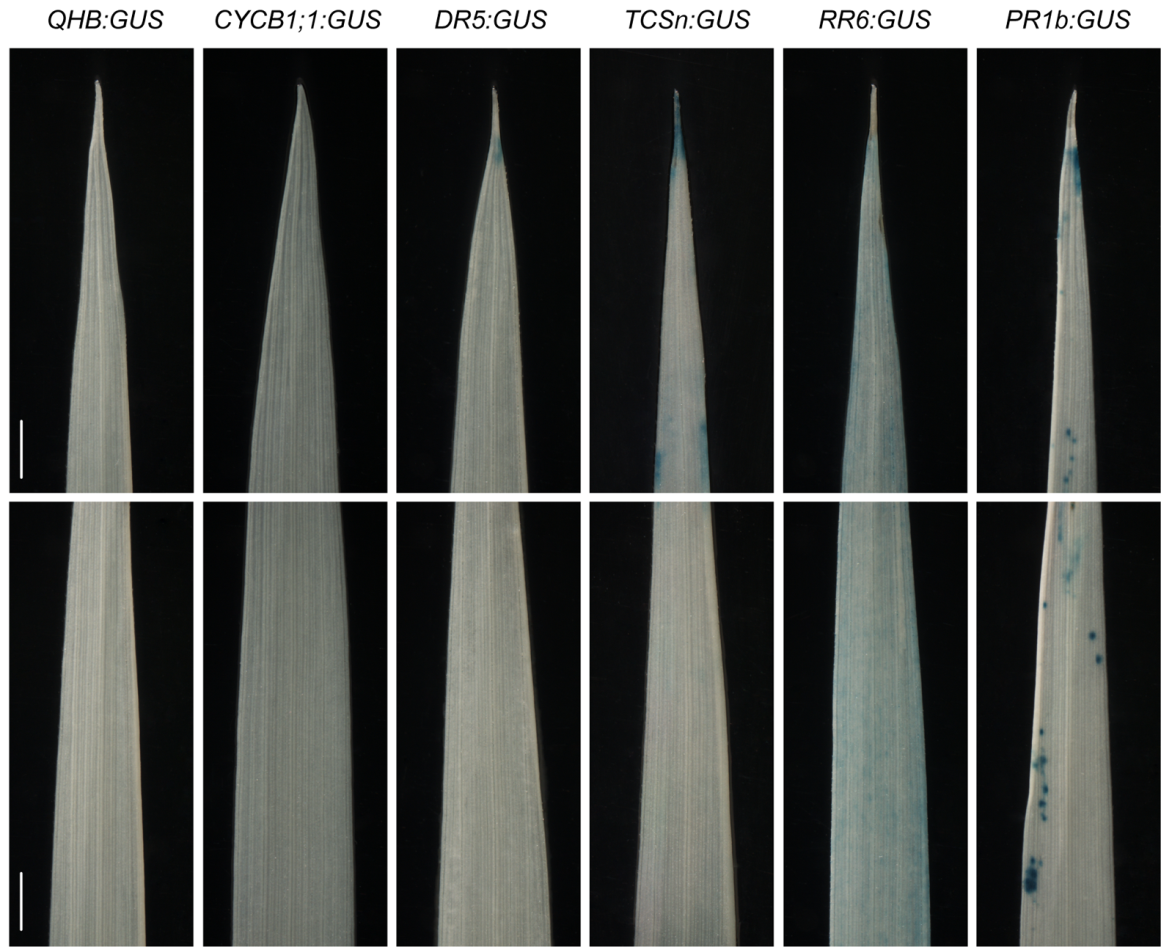


**Supplementary Figure 7.** Specific GUS staining patterns in the leaves of *QHB/CYCB1;1/DR5/TCSn/RR6/PR1b:GUS* transgenic lines selected by vectors based on *CSP1-HPT-MCS-GUS*. GUS expressions of the first fully expended leaf of 8-day-old seedlings were analysed. Leaf tips (upper panels) and adjacent part near the leaf tips (lower panels) are shown. Bars, 2 mm. Images are representative GUS expression patterns in T_1_ generation of at least three lines.


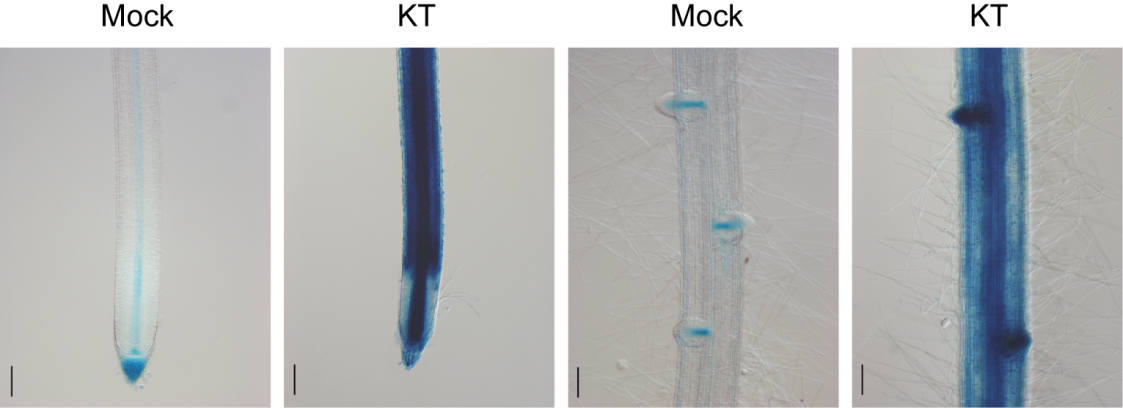


**Supplementary Figure 8.** Cytokinin responsiveness of *RR6:GUS* transgenic plant transformed by the *CSP1* controlled vector. 8-day-old *RR6:GUS* transgenic seedlings (T_1_) were treated with 100 μM KT in fresh culture solution for 24 h or without KT as mock treatment. Primary root tips (left two panels) and mature root zone with lateral roots (right two panels) were dissected for GUS staining. Strongly induced GUS expressions in the entire root is clearly shown after 7 h staining. Bars, 100 μm. Images are representative GUS expression patterns of at least three lines.


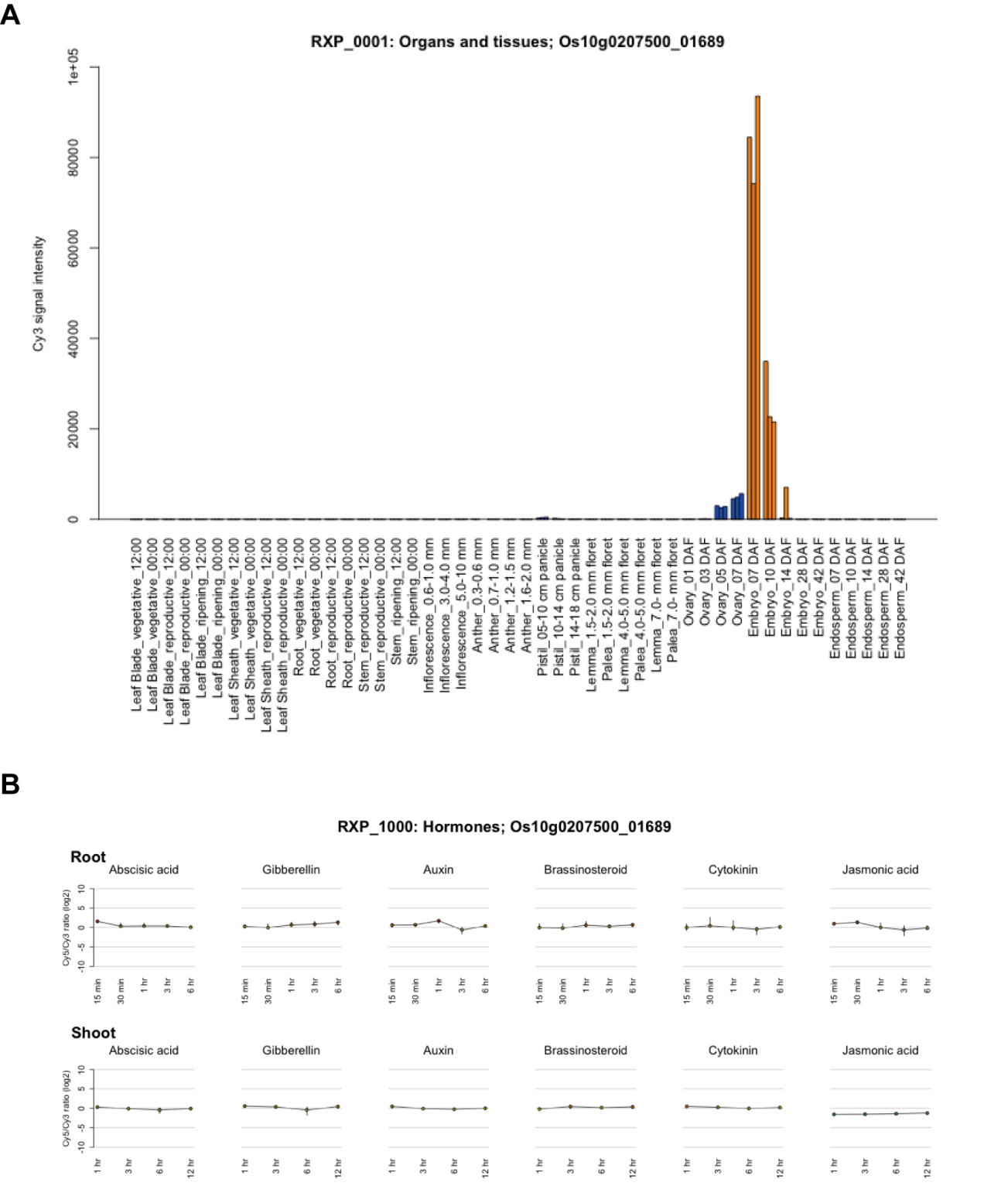


**Supplementary Figure 9.** Expression profile of *CSP1* analysed on the RiceXpro platform (http://ricexpro.dna.affrc.go.jp/). **(A)** The spatial temporal expression profile. **(B)** Expression profile in response to plant hormones in root and shoot.


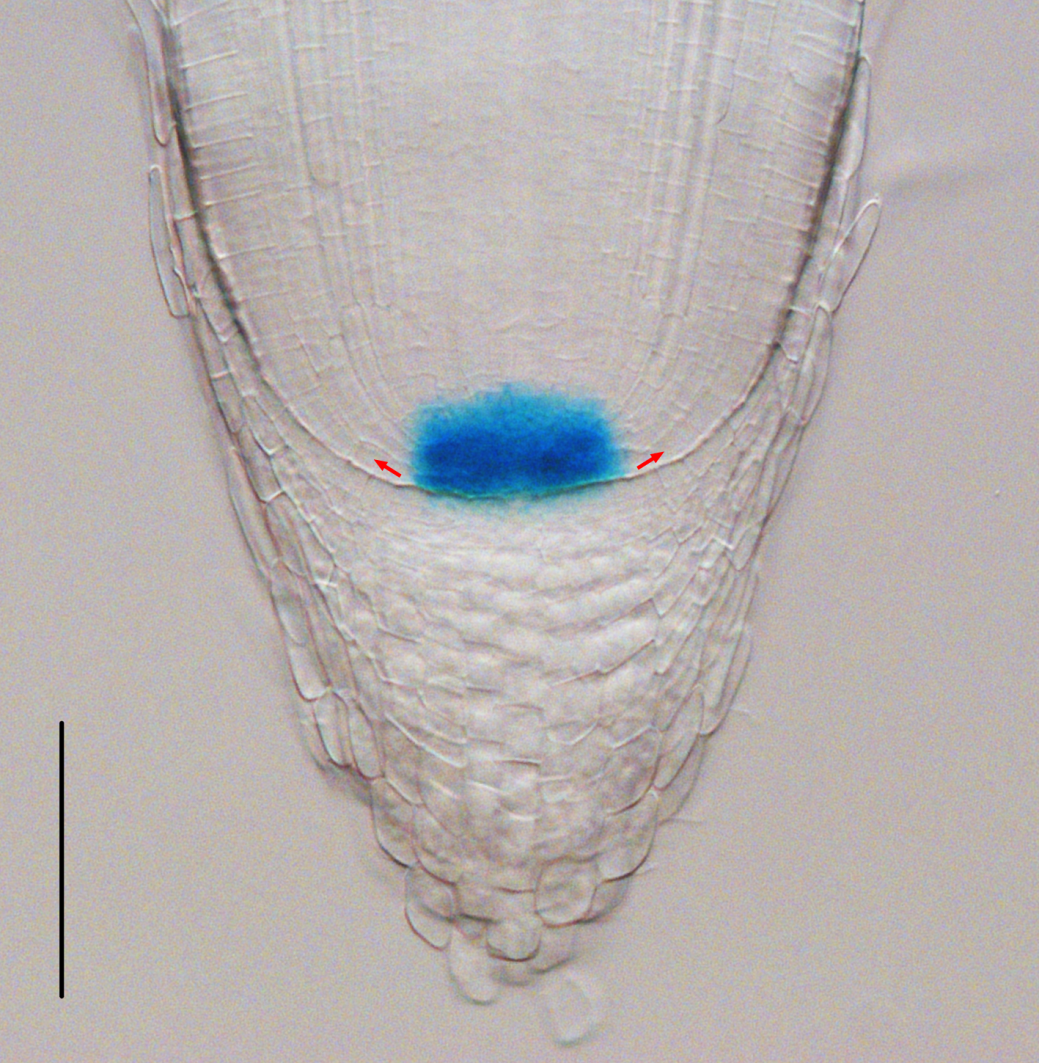


**Supplementary Figure 10.** The expression pattern of *QHB* in the root tip of rice. Primary root tip of 7-day-old *QHB:GUS* transgenic seedling (T_1_) was subjected to GUS staining for 6 h and observed under microscope with 20x objective lens after clearing. GUS expression was found in the center of the root meristem containing the QC and its surrounding stem cells. The red arrows indicate the single layer of epidermal cells originating from the stem cells near the QC. Bars: 50 μm. Images are representative GUS expression patterns of at least three lines.


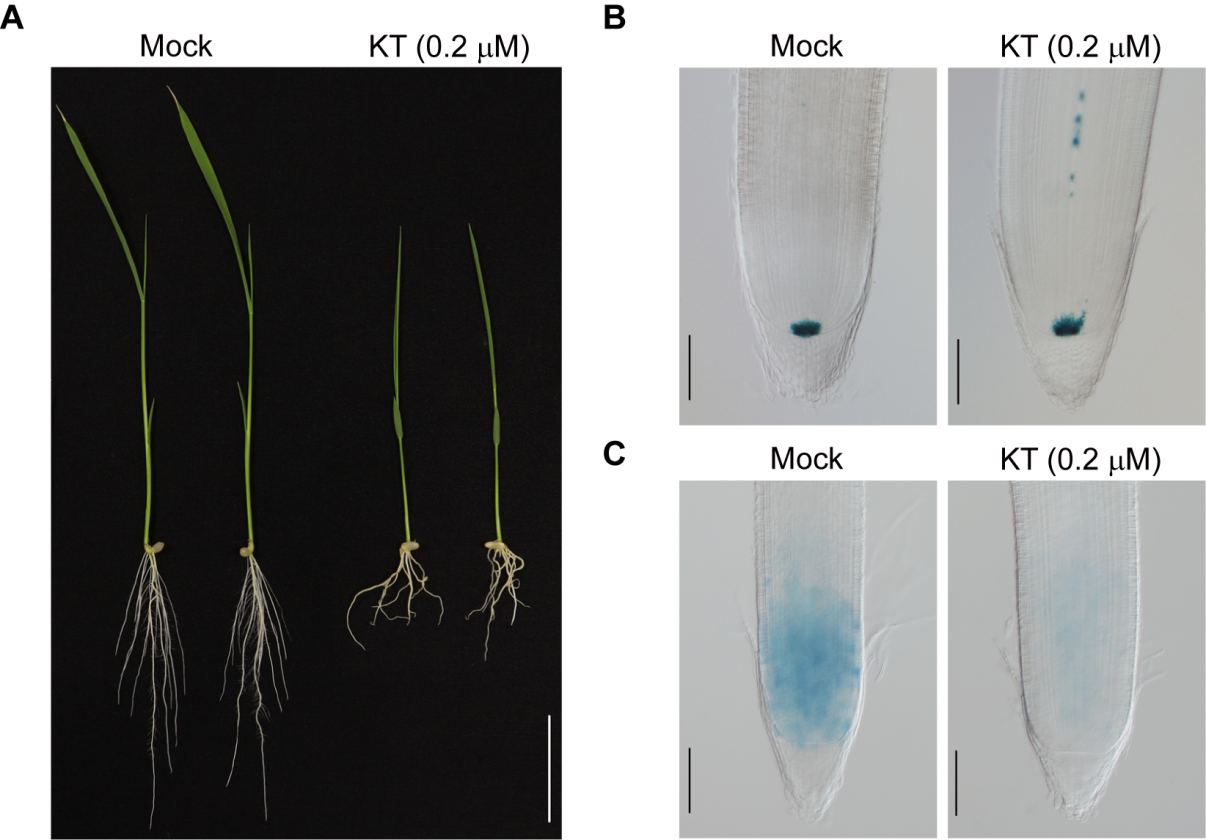


**Supplementary Figure 11.** GUS expression activity of *QHB* and *CYCB1;1* in root tip after cytokinin treatment at low level. **(A)** Root growth inhibition of *QHB:GUS* and *CYCB1;1:GUS* transgenic seedlings (T_3_) grown on solution cultures with or without 0.2 μM KT for 7 days. Bars: 2.5 cm. **(B)** *QHB:GUS* expression in the quiescent center of the mock and KT treated roots after GUS staining for 16 h. Bars: 100 μm. **(C)** *CYCB1;1:GUS* expression in the meristematic region of the mock and KT treated roots after GUS staining for 1 h. Bars: 100 μm. Images are representative GUS expression patterns of at least three lines.

**Supplementary Table 1.** Primers used for plasmid construction

**Primer Name Primer Sequence (5’-3’)**

CSP1-Pr-F1 AGAGCTCAGTCTGGACATGATTGGGTTATGAG
CSP1-Pr-R1 AGGTACCTGCCTTCTCTGTATAACCTGCAGC
CSP1-Pr-F2 AGGCGCGCCAGTCTGGACATGATTGGGTTATGAG
CSP1-Pr-R2 AGGCGCGCCTGCCTTCTCTGTATAACCTGCAGC
CSP1-Pr-FW CTTTTTCATGGCGCGCCTGCCTTCTCTGTATAACCTGC
CSP1-Pr-RV CGGGAGCTCGGCGCGCCAGTCTGGACATGATTGGGTTATG
QHB-Pr-F AGGATCCGCCTCATGCGGACTTAACGGGTTG
QHB-Pr-R AGGTACCGGGCCCCCCCTCGA
CYCB1;1-Pr-F TATAGACTAAGCCATTGAGGCGTATG
CYCB1;1-Pr-R AGAGCTGATCTCGATGACATGC
CYCB1;1-Pr-FW CCGGGGATCCTCTAGTATAGACTAAGCCATTGAGG
CYCB1;1-Pr-RV TACCGAATTCGTCGACAGAGCTGATCTCGATGACA
RR6-Pr-F ATCTAGACGCTATTAACACCTCGTTTTGC
RR6-Pr-R AGTCGACTGATCAACTCAAAATCCAACCC
PR1b-Pr-FW CCGGGGATCCTCTAGCAATTTGATTCCCTCCAGAA
PR1b-Pr-RV TACCGAATTCGTCGAAGTTAATCAAGCTCACACTTA

The underlined bases at 5’ end of primers indicate the restriction sites, the bases in gray shade indicate the overlap sequences for In-Fusion cloning.

**Supplementary Table 2.** Vectors used in experiments

**Vector Name Configuration Experiment Figure**

CSP1:GUS tCUP1-HPT-CSP1-GUS *GUS* expression Figure 1
35S-HPT-GUS 35S-HPT-MCS-GUS *HPT* and *GUS* expression Figure 2,4,S3
CSP1-HPT-GUS CSP1-HPT-MCS-GUS *HPT* and *GUS* expression Figure 2,4
rCSP1-HPT-GUS rCSP1-HPT-MCS-GUS *HPT* and *GUS* expression Figure S3
HPT-35S-GUS HPT-35S-MCS-GUS *GUS* expression, copy number Figure 3, 4
HPT-CSP1-GUS HPT-CSP1-MCS-GUS *GUS* expression, copy number Figure 3, 4
QHB:GUS CSP1-HPT-QHB-GUS *GUS* expression Figure 5, 6, S7, S10, S11
CYCB1;1-GUS CSP1-HPT-CYCB1;1-GUS *GUS* expression Figure 5, 6, S7, S11
DR5-GUS CSP1-HPT-DR5-GUS *GUS* expression Figure 5, 6, S5, S7
TCSn-GUS CSP1-HPT-TCSn-GUS *GUS* expression Figure 5, 6, S6, S7
RR6-GUS CSP1-HPT-RR6-GUS *GUS* expression Figure 5, S7, S8
PR1b-GUS CSP1-HPT-PR1b-GUS *GUS* expression Figure 5, S7

**Supplementary Table 3.** Primers used for Quantitative real-time PCR analyses and Southern Blot

**Primer Name Primer Sequence (5’-3’)**

HPT-RT-F CGCCGATGGTTTCTACAAAG
HPT-RT-R ACACATGGGGATCAGCAATC
GUS-RT-F TAGATCTGAGGAACCGACGAAC
GUS-RT-R GCCGAAGCGGAGCACGATAC
CSP1-RT-F AGTCATCTTCCTCCGCGATGCAGTC
CSP1-RT-R TCTGGTACACCACCACGTTCTGCTC
OsAct-RT-F GAGTATGATGAGTCGGGTCCAG
OsAct-RT-R ACACCAACAATCCCAAACAGAG
HPT-Probe-F ATTCCCAATACGAGGTCGCCAA
HPT-Probe-R CTTCTACACAGCCATCGGTCCA

**Supplementary Table 4.** Callus weight at the start of the first (S1) and second (S2) rounds of selection

Vector Plate S1-1 S1-2 S2-1 S2-2 Fold-1 Fold-2
 (mg) (mg) (mg) (mg) (S2-1/S1-1) (S2-2/S1-2)

 *35S-HPT-GUS* 1 0.9373 1.0206 2.2384 2.3825 2.3881 2.3344
 2 1.0355 0.9901 2.2087 2.1597 2.1330 2.1813
 3 0.8976 1.1951 2.0084 2.0324 2.2375 1.7006
 4 0.5958 0.7891 1.4561 2.1761 2.4439 2.7577
 5 0.8300 0.6931 1.8875 2.0323 2.2741 2.9322
 6 0.8300 2.0376 2.4549
 Average 0.8592 0.9197 1.9598 2.1368 2.2953 2.3935
 SD 0.1650 0.1830 0.3166 0.1372 0.1233 0.4371

 *CSP1-HPT-GUS* 1 1.0530 1.0092 2.0892 2.0972 1.9840 2.0781
 2 0.9948 1.1170 2.0799 2.2342 2.0908 2.0002
 3 0.8932 0.8522 1.9454 1.9791 2.1780 2.3223
 4 0.9751 1.1696 1.9916 2.7120 2.0425 2.3187
 5 0.4916 0.8463 1.1999 1.9970 2.4408 2.3597
 6 0.6660 0.6231 1.5329 1.7265 2.3017 2.7708
 Average 0.8456 0.9362 1.8065 2.1243 2.1730 2.3083
 SD 0.2199 0.2028 0.3608 0.3328 0.1719 0.2701

**Supplementary Table 5.** GUS expression patterns in roots of *DR5:GUS* T_0_ transgenic plants

Patterns in T_0_ adventious roots

Line c d h g Total

1 1 1 4 6
 2 1 3 4
 3 7 7
 4 7 7
 5 7 7
 6 1 5 6
 7 6 6
 8 2 4 6
 9 1 5 6
 10 1 5 6
 11 2 1 1 3
 12 2 4 6
 13 1 2 3
 15 6 6
 16 4 4
 17 4 2 6
 18 2 2
 19 3 3 6
 20 7 7
 21 2 5 7
 22 1 5 6
 23 2 3 5
 24 3 3
 25 1 1 2
 26 6 6
 28 5 5
 29 6 6
 30 2 4 6
 31 1 4 5
 32 2 2 4
 33 1 1 2
 34 2 2 4
 35 2 1 3
 36 2 2 4
 37 1 4 1 6
 38 1 2 3
 39 4 4
 40 3 2 5
 41 3 2 5
 43 3 3 6
 47 3 3
 48 5 5
 49 3 2 5
 50 3 3

Continue

Patterns in T_0_ adventious roots

Line c d h g Total

51 4 4
 52 3 3
 53 2 1 3
 54 2 4 6
 55 2 1 2 5
 57 2 1 3
 58 1 1
 59 2 1 4 7
 60 3 1 4
 62 5 5
 63 1 4 5
 64 1 4 5
 65 1 1
 67 3 1 1 5
 68 4 4
 70 3 1 4
 71 1 2 3
 72 2 2
 74 1 4 5
 75 3 3
 76 5 5
 78 1 3 4
 79 3 1 4
 80 1 4 5
 81 5 5
 82 1 4 5
 83 8 8
 85 5 5
 86 3 2 5
 87 4 4
 88 2 2 4
 89 1 5 6
 91 1 1 2
 Total 95 32 177 54 357
 Percent 26.61% 8.96% 49.58% 15.13%

The *DR5:GUS* staining patterns were described same as that in Zhou *et al*., 2014.

Patern c: in root cap, quiescent center and protoxylem

Patern d: in root cap and basal meristematic zone

Patern h: in root cap and entire meristematic zone

Patern g: in basal meristematic and elongation zones, absent in root cap and promeristem

**Supplementary Sequence 1.** Sequence of the *CSP1* promoter amplified (1996bp)

+ GTCTGGACAT GATTGGGTTA TGAGAACATT AGCCAATAGA ACTGCTTGTT ACAACATGTT TAGGATGCAC

- CAGACCTGTA CTAACCCAAT ACTCTTGTAA TCGGTTATCT TGACGAACAA TGTTGTACAA ATCCTACGTG

+ AGGCCAGTTT TTGAGAGGTT ACATTCTGTG CTTGTGGAAT CATATGAGTT GAAGTCCACA AACAAAATAG

- TCCGGTCAAA AACTCTCCAA TGTAAGACAC GAACACCTTA GTATACTCAA CTTCAGGTGT TTGTTTTATC

+ ATTCCATGGA GTGTTTGGGT CTTTTTTTTG TGGATAGTAG GTGCTCCCCA ATCAGTTAGG CAAGCCCAAG

- TAAGGTACCT CACAAACCCA GAAAAAAAAC ACCTATCATC CACGAGGGGT TAGTCAATCC GTTCGGGTTC

+ ACCGATTTGT TAGGTCTCTT AGAACAGTAC ATAGTAAGTT CAAAGCAGTT TTGACCGCTC TACTGAAACT

- TGGCTAAACA ATCCAGAGAA TCTTGTCATG TATCATTCAA GTTTCGTCAA AACTGGCGAG ATGACTTTGA

+ TGCAAAAGAT ATCATTAGGC CAAAAGATCC CTTATTCACA ACAGTCCATA AGAAATTGCT TTCTCCACAG

- ACGTTTTCTA TAGTAATCCG GTTTTCTAGG GAATAAGTGT TGTCAGGTAT TCTTTAACGA AAGAGGTGTC

+ TACACACCAT ATTTGGATAA TTGCATTGGA GCAATAGATG GTACTCACAT CCAAGTTGTG GTGCCAAATT

- ATGTGTGGTA TAAACCTATT AACGTAACCT CGTTATCTAC CATGAGTGTA GGTTCAACAC CACGGTTTAA

+ CAGCTGCTGT TCAACATAGG AATAGACATA AGGAGAAGAG TCAGAATGTT ATGTTTGTCT GTGACTTTGA

- GTCGACGACA AGTTGTATCC TTATCTGTAT TCCTCTTCTC AGTCTTACAA TACAAACAGA CACTGAAACT

+ TATGAGATTT ACTTTCGTGC TTGCTGGCTG GCCTGGTTCG GTTCATGACA TGAGGGTATT CAATGATGCA

- ATACTCTAAA TGAAAGCACG AACGACCGAC CGGACCAAGC CAAGTACTGT ACTCCCATAA GTTACTACGT

+ CAAACTAGAT TTAGTGCCAA GTTTCCAAAG CCACCTCCAG GAAAGTTTTA TCTCGTAGAC TCGGGATACC

- GTTTGATCTA AATCACGGTT CAAAGGTTTC GGTGGAGGTC CTTTCAAAAT AGAGCATCTG AGCCCTATGG

+ CAAATAGGCT GGGTTATCTA GCACCATACA AGGGTATAAC ATATCATTCC AAGAGTACAA CGAAAGCACA

- GTTTATCCGA CCCAATAGAT CGTGGTATGT TCCCATATTG TATAGTAAGG TTCTCATGTT GCTTTCGTGT

+ TTGCCAAGGG GAAGAAGGGA GCACTTTAAT TACTGCCATT CTTCGTGTCG GAATGTCATA GAGAGGTCAT

- AACGGTTCCC CTTCTTCCCT CGTGAAATTA ATGACGGTAA GAAGCACAGC CTTACAGTAT CTCTCCAGTA

+ TTGGGGTCTT GAAGAACAAG TGGAGGATTT TGTTTAGTTT ACCTAGCTAC TCGCAGGAAA AACAAAGCAG

- AACCCCAGAA CTTCTTGTTC ACCTCCTAAA ACAAATCAAA TGGATCGATG AGCGTCCTTT TTGTTTCGTC

+ AATTATCCAT GCATGCATAG CACTTCATAA TTTCATTAGA GACAGTCAAA TGGCTAATAC AGAGTTCGAC

- TTAATAGGTA CGTACGTATC GTGAAGTATT AAAGTAATCT CTGTCAGTTT ACCGATTATG TCTCAAGCTG

+ AATTGTGACC ATGATGAAAA TTATGATCCA TTGGGTGGAA CCTCTGCTCC ATCTAGTGAA CCAACAAACG

- TTAACACTGG TACTACTTTT AATACTAGGT AACCCACCTT GGAGACGAGG TAGATCACTT GGTTGTTTGC

+ ATTTGGACTC GCGTGTCATG AACCAATTTC GGGACTGGGT TGCCGATGGA TTGTGGTCTT TGAAAGGAAT

- TAAACCTGAG CGCACAGTAC TTGGTTAAAG CCCTGACCCA ACGGCTACCT AACACCAGAA ACTTTCCTTA

+ GTAATGAATG GTTCAAATTT ATTGTGAATT GTATTTGGTT TAACTTGTCA ATTGAACCAT GAGTGCTTGT

- CATTACTTAC CAAGTTTAAA TAACACTTAA CATAAACCAA ATTGAACAGT TAACTTGGTA CTCACGAACA

+ AATTTGTAAC ATTGAAACTT CTGTGATGTA TTTCAACAAC TTGAGATGTG TATGAGCTTC TTTGAATTTC

- TTAAACATTG TAACTTTGAA GACACTACAT AAAGTTGTTG AACTCTACAC ATACTCGAAG AAACTTAAAG

+ TGTGATTAGG CTGTGATATT GATGATGAAA ATGATGTGGG GGTTATTGCT TGTGATTCGG CTTGCTGAAG

- ACACTAATCC GACACTATAA CTACTACTTT TACTACACCC CCAATAACGA ACACTAAGCC GAACGACTTC

+ GTGTGTATTT GTCCAAATTA TGATGGATTA AAACATGTGA TGTTGGGGTG AAACATTGAT TTGGCTTGCA

- CACACATAAA CAGGTTTAAT ACTACCTAAT TTTGTACACT ACAACCCCAC TTTGTAACTA AACCGAACGT

+ TTTGGTTGTG CTGTCATGCA GCAACAATGG TTTTGCTTGT AAAGTGCTAG CAGCGGCAGC AAACAGCATG

- AAACCAACAC GACAGTACGT CGTTGTTACC AAAACGAACA TTTCACGATC GTCGCCGTCG TTTGTCGTAC

+ CTGCAGCAAG GGGCATGGTG CAGTCCACTT TGTATGGTTT TAGGGGGTAA TATGGTCATT GTGCAGCGCA

- GACGTCGTTC CCCGTACCAC GTCAGGTGAA ACATACCAAA ATCCCCCATT ATACCAGTAA CACGTCGCGT

+ TTTAATAAAC TTTAGTCCCT TTTAGTCCCT ATAACCAAAC AGTTATGGAC TAAAATGATT AGTCCTAAGA

- AAATTATTTG AAATCAGGGA AAATCAGGGA TATTGGTTTG TCAATACCTG ATTTTACTAA TCAGGATTCT

+ CTAAAATAAG TCCCTAGGAC TTATGGATCC AAACACCACC ATAGTAGAAG CTATGGGCGG GGGCGGCAGT

- GATTTTATTC AGGGATCCTG AATACCTAGG TTTGTGGTGG TATCATCTTC GATACCCGCC CCCGCCGTCA

+ CCGGGTGGCA GCATCTTCGA TAGAGTGGCA GCCGTCCACC GGCCGCTTCA TCGCGCGTGG CGGCAGTCCA

- GGCCCACCGT CGTAGAAGCT ATCTCACCGT CGGCAGGTGG CCGGCGAAGT AGCGCGCACC GCCGTCAGGT

+ CCCAATATGC CGTCGTGGGT AACCAATCGG CATCGAGACC TATCCGCGGG ACGAAGCCAA CCGTTCGAAT

- GGGTTATACG GCAGCACCCA TTGGTTAGCC GTAGCTCTGG ATAGGCGCCC TGCTTCGGTT GGCAAGCTTA

+ GGTGTGCGCG CGTGCATGCA TGCGCGCGGT CTCAGTTAAT GCATTGCCTC TCCCGAGTCA AGTGGCGTGC

- CCACACGCGC GCACGTACGT ACGCGCGCCA GAGTCAATTA CGTAACGGAG AGGGCTCAGT TCACCGCACG

+ TATGTTTGCA GAGGCAAACA TGCATGAGCA GATGGCCTGG TGAACTTATA TACTCCCGGC AGGCAAGTGA

- ATACAAACGT CTCCGTTTGT ACGTACTCGT CTACCGGACC ACTTGAATAT ATGAGGGCCG TCCGTTCACT

+ AGTGCATGCC TGTATAGCTT GAGCAAGTAT TTGGCAGTTT GCTGCCAACT GCTACCCTAT TTCAAATCTA

- TCACGTACGG ACATATCGAA CTCGTTCATA AACCGTCAAA CGACGGTTGA CGATGGGATA AAGTTTAGAT

+ GGTAAGATCT AGCTGCAGGT TATACAGAGA AGGC

- CCATTCTAGA TCGACGTCCA ATATGTCTCT TCCG

Cis elements related to responsiveness of light, drought, cold, anaerobic induction and involed in seed or meristem specific regulation were indicated: I-box: CCAATAGAT, ACA motif: AAT TACTGCCATT, RY-element: CAT GCATG, LTR: AAAG CC, ARE: ACCAA A, Sp1: GGGCGG, GC-motif: GCC CCC, CAT-box: TCACCG, G-box: CAGCAC, MBS: CAACT G, TCT-motif: CATTCT. The function of these cis-elements were listed in **Supplementary Excel 2**.

**Supplementary Sequence 2.** Synthesized sequence of *TCSn1* (282bp)

(SalI)GTCGACcaaagatctttaaaagattttgaaagatctctccaaaatcctttcaaagatctttaaaagatttataaaaatctttgcaaaatccaaccaaagattttgtaaagattttgcaagatccgatcaaaatctttagctagtcaaagatctttaaaagattttgaaagatctctccaaaatcctttcaaagatctttaaaagatttataaaaatctttgcaaaatccaaccaaagattttgtaaagattttgcaagatccgatcaaaatctttaCTGCAG(PstI)

**Supplementary Sequence 3.** PstI digested mini35S-TMVΩ fragment for ligation of *TCSn1* (147bp)

(PstI)ctgcagccccgcaagacccttcctctatataaggaagttcatttcatttggagaggtatttttacaacaattaccaacaacaacaaacaacaaacaacattacaattactatttacattacagggaggtaccatcgtcgacctgcag(KpnI-PstI)
