# Supplementary material for: Targeted Transgene Expression in Rice Using a Callus Strong Promoter for Selectable Marker Gene Control
Source: Front Plant Sci. 2020 Dec 11;11:602680. doi: 10.3389/fpls.2020.602680 (PMC7759479; doi:10.3389/fpls.2020.602680)
Supplement: Supplementary PDF 1 — Perturbation analysis of the CSP1 gene by GENEVESTIGATOR. [file Data_Sheet_1.PDF]

Dataset: 1064 perturbations from data selection: OS_AFFY_RICE-1

Showing 1 measure(s) of 1 gene(s) on selection: OS-0

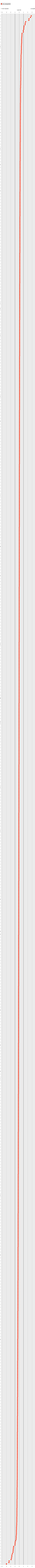

1064 of 1064 perturbations fulfilled the filter criteria

Filter values for selected measure(s)

Oryza sativa (1064)	Pi-score	Log2-ratio (no filter)	Fold-Change	p-value (no filter)
OS-00037 (27) temperature (Zhenshan 97; 31°C; 15dai) / untreated calli samples (Z...	3.54	4.63	10.26	0.171
OS-00037 (46) Zhenshan 97 / MH63 [temperature (Zhenshan 97; 31°C; 15dai) / tem...	5.32	2.81	6.91	0.013
OS-00048 (12) diurnal (8:00pm; day 2; leaf) / diurnal (8:00am; day 2; leaf)	3.67	2.65	6.68	0.041
OS-00037 (1) callus growth (MH63; screening) / callus growth (MH63; 26°C; 15das)	5.51	2.30	4.91	0.004
OS-00037 (28) Zhenshan 97 / MH63 [callus growth (Zhenshan 97; 26°C; 15das) / cal...	2.67	2.25	5.16	0.065
OS-00048 (18) diurnal (0:00am; day 2; leaf) / diurnal (8:00am; day 1; leaf)	2.58	1.52	2.91	0.020
OS-00035 (15) caryopsis development (bottom 1/4; 4dap) / caryopsis development (...	2.09	1.42	2.91	0.034
OS-00081 (1) CSSL50-1 / Asominori	2.14	1.38	2.72	0.028
OS-00035 (13) caryopsis development (bottom 1/4; 2dap) / caryopsis development (...	2.79	1.14	2.19	0.003
OS-00079 (1) M. graminicola (2dpi) / untreated root samples	1.38	1.09	2.04	0.055
OS-00048 (16) diurnal (12:00am; day 2; leaf) / diurnal (8:00am; day 2; leaf)	1.25	1.08	2.13	0.070
OS-00225 (3) dehydration (IR20; 3h) / untreated seedling samples (IR20)	2.04	0.96	1.94	0.007
OS-00079 (2) M. graminicola (4dpi) / untreated root samples	1.15	0.96	1.83	0.064
OS-00035 (14) caryopsis development (bottom 1/4; 3dap) / caryopsis development (...	1.01	0.67	1.59	0.031
OS-00225 (4) dehydration (IR20; 6h) / untreated seedling samples (IR20)	1.08	0.65	1.56	0.021
OS-00284 (5) HFL1 / 35S-15	0.29	0.61	1.72	0.331
OS-00061 (33) IR24 / Nipponbare [mock treated leaf samples (IR24) / mock treated l...	1.11	0.60	1.51	0.014
OS-00126 (2) temperature (NIL(Hwi1); shift 20°C to 30°C; 3d) / temperature (NIL(Hwi...	0.49	0.58	1.55	0.141
OS-00037 (2) callus growth (Zhenshan 97; screening) / callus growth (Zhenshan 97;...	0.21	0.58	1.37	0.438
OS-00116 (1) seed deterioration (40°C) / embryo samples from mock treated dry se...	1.13	0.58	1.48	0.011
OS-00225 (2) dehydration (Dhagaddeshi; 6h) / untreated seedling samples (Dhaga...	0.77	0.56	1.48	0.042
OS-00037 (29) Zhenshan 97 / MH63 [callus growth (Zhenshan 97; screening) / callu...	0.72	0.53	1.44	0.045
OS-00105 (7) IRBB21 / IR24 [X. oryzae pv. oryzae (IRBB21; 24hpi) / X. oryzae pv. oryza	0.87	0.52	1.42	0.021
OS-00234 (1) optimal fertilization (93-11; leaf) / nutrient deficiency (93-11; leaf)	0.50	0.51	1.46	0.107
OS-00009 (1) anoxia / aerobic germinated coleoptile samples	0.99	0.51	1.42	0.011
OS-00239 (8) Dular / Pusa Basmati 1 [phosphorus (Dular; 320µM Pi; shoot) / phosp...	0.48	0.49	1.41	0.106
OS-00239 (7) Dular / Pusa Basmati 1 [phosphorus (Dular; 320µM Pi; root) / phospho...	0.80	0.49	1.40	0.023
OS-00061 (42) IR24 / Nipponbare [X. oryzae pv. oryzae (IR24; T7174) / X. oryzae pv. or.	1.16	0.46	1.38	0.003
OS-00225 (1) dehydration (Dhagaddeshi; 3h) / untreated seedling samples (Dhaga...	0.71	0.43	1.35	0.023
OS-00035 (7) anther development (zygotene-pachytene) / anther development (leptot...	0.77	0.43	1.35	0.016
OS-00061 (41) IR24 / Nipponbare [X. oryzae pv. oryzae (IR24; PXO99AME7) / X. oryza	0.67	0.43	1.35	0.027
OS-00035 (6) anther development (tricellular pollen) / anther development (bicellular...	0.39	0.42	1.32	0.116
OS-00221 (3) salt (IR64; ABA; shoot) / salt (IR64; shoot)	0.40	0.41	1.33	0.109
OS-00234 (15) PA64s / 93-11 [nutrient deficiency (PA64s; leaf) / nutrient deficiency (9...	0.75	0.40	1.32	0.014
OS-00037 (33) Zhenshan 97 / MH63 [endosperm development (Zhenshan 97; 7dap)...	0.31	0.40	1.31	0.166
OS-00037 (37) Zhenshan 97 / MH63 [germination (Zhenshan 97) / germination (MH6...	0.25	0.40	1.32	0.233
OS-00037 (18) panicle development (MH63; 3-5mm) / panicle development (MH63; ...	0.72	0.40	1.31	0.016
OS-00093 (4) short day (pistil and stamen primordia formation) / short day (glume pr...	0.26	0.39	1.33	0.219
OS-00068 (3) drought (IRAT109; leaf RWC 83-88%) / untreated flag leaf samples (IR...	0.41	0.39	1.31	0.087
OS-00051 (7) anoxia (N2 for 27h) / imbibition (aerobic; 27h)	0.45	0.38	1.30	0.064
OS-00102 (7) cold (K354; 6h) / untreated shoot samples (K354)	0.47	0.37	1.29	0.054
OS-00126 (5) temperature (NIL(hwi1); shift 20°C to 30°C; 15d) / temperature (NIL(hw...	0.44	0.37	1.29	0.062
OS-00037 (25) panicle development (Zhenshan 97; heading) / panicle development ...	0.40	0.36	1.29	0.078
OS-00238 (10) collar development (3rd leaf; S6) / collar development (3rd leaf; S2)	0.23	0.36	1.27	0.234
OS-00283 (1) IAA (crl1; 2h) / untreated crown samples (crl1)	0.66	0.36	1.29	0.015
OS-00234 (7) LYP9 / 93-11 [nutrient deficiency (LYP9; leaf) / nutrient deficiency (93-1...	0.68	0.36	1.28	0.013
OS-00037 (22) panicle development (Zhenshan 97; 3-5mm) / panicle development (...	0.75	0.35	1.28	0.007
OS-00235 (13) Nagina 22 / Moroberekan [drought; heat (Nagina 22; pistil; 1x6h) / dro...	0.40	0.34	1.27	0.066
OS-00068 (2) drought (IRAT109; leaf RWC 74-78%) / untreated flag leaf samples (IR...	0.28	0.34	1.26	0.151
OS-00221 (4) salt (IR64; ABA; shoot) / untreated shoot samples (IR64)	0.27	0.33	1.26	0.149
OS-00238 (9) collar development (3rd leaf; S5) / collar development (3rd leaf; S2)	0.18	0.32	1.24	0.278
OS-00041 (4) drought (panicle; booting) / untreated panicle samples (booting)	0.37	0.31	1.24	0.062
OS-00121 (5) ammonium (Nipponbare; 2mM (NH4)2SO4; root) / ammonium (Nippo...	0.32	0.31	1.24	0.090
OS-00022 (2) Nip.100Gy / Nipponbare	0.31	0.31	1.24	0.097
OS-00288 (2) simulated drought (Nagina 22) / untreated leaf samples (Nagina 22)	0.17	0.30	1.23	0.268
OS-00063 (3) anther development (tricellular pollen) / anther development (microspo...	0.28	0.30	1.24	0.118
OS-00100 (4) CJF1 / Jamaica [root]	0.15	0.30	1.22	0.307
OS-00108 (10) imbibition (24h; endosperm) / endosperm samples from dry seeds	0.45	0.30	1.23	0.030
OS-00134 (1) OX-Ghd7HJ19 / Hejiang 19 [leaf]	0.12	0.30	1.23	0.390
OS-00126 (4) temperature (NIL(Hwi1); shift 20°C to 30°C; 7d) / temperature (NIL(Hwi...	0.19	0.30	1.24	0.234
OS-00103 (11) cold (LTH; 4°C for 48h; 29°C for 24h) / cold (LTH; 4°C for 48h)	0.23	0.29	1.22	0.162
OS-00277 (2) X. oryzae pv. oryzae (BXO1002) / mock treated leaf central vein samples	0.32	0.29	1.22	0.077
OS-00128 (12) MRP 5401 / PR 319 [roots]	0.34	0.29	1.22	0.066
OS-00126 (1) temperature (NIL(hwi1); shift 20°C to 30°C; 3d) / temperature (NIL(hwi...	0.16	0.28	1.23	0.261
OS-00108 (8) imbibition (16h; endosperm) / endosperm samples from dry seeds	0.59	0.28	1.21	0.008
OS-00004 (2) trans-zeatin (30min; root) / mock treated root samples (30min)	0.36	0.28	1.22	0.051
OS-00032 (1) salt (salt sensitive RILs) / untreated seedling samples (salt sensitive ...	0.13	0.28	1.22	0.337
OS-00051 (9) anoxia (N2 for 30h) / imbibition (aerobic; 30h)	0.45	0.28	1.21	0.023
OS-00047 (2) pollen development (tricellular immature) / pollen development (bicell...	0.50	0.28	1.21	0.015
OS-00037 (12) endosperm development (Zhenshan 97; 21dap) / endosperm develo...	0.10	0.27	1.22	0.427
OS-00234 (16) PA64s / 93-11 [nutrient deficiency (PA64s; root tip) / nutrient deficienc...	0.48	0.27	1.21	0.018
OS-00061 (34) IR24 / Nipponbare [untreated leaf samples (IR24) / untreated leaf sa...	0.28	0.27	1.21	0.092
OS-00126 (6) temperature (NIL(Hwi1); shift 20°C to 30°C; 15d) / temperature (NIL(H...	0.14	0.27	1.22	0.299
OS-00128 (11) MRP 5401 / PR 319 [leaf]	0.10	0.27	1.22	0.416
OS-00239 (5) Dular / Pusa Basmati 1 [phosphorus (Dular; 1µM Pi; root) / phosphoru...	0.38	0.26	1.20	0.035
OS-00064 (2) pollen development (tricellular) / pollen development (bicellular)	0.27	0.26	1.20	0.090
OS-00037 (10) endosperm development (MH63; 21dap) / endosperm development (...	0.28	0.26	1.20	0.081
OS-00052 (2) imbibition (aerobic; 3h) / embryo samples from dry seeds	0.14	0.26	1.22	0.286
OS-00225 (7) Dhagaddeshi / IR20 [untreated seedling samples (Dhagaddeshi) / unt...	0.13	0.26	1.19	0.306
OS-00119 (1) ABA (3h) / mock treated seedling samples (3h)	0.14	0.26	1.19	0.276
OS-00068 (4) drought (Zhenshan 97; leaf RWC 65-69%) / untreated flag leaf sample...	0.09	0.25	1.20	0.428
OS-00061 (35) IR24 / Nipponbare [X. oryzae pv. oryzae (IR24; PXO86) / X. oryzae pv. o	0.14	0.25	1.19	0.288
OS-00055 (24) IRBB7 / IRBB5 [X. oryzae pv. oryzae (IRBB7; 6hpi) / X. oryzae pv. oryza	0.21	0.25	1.19	0.144
OS-00121 (20) OsAnt1::AlaAT (1/7) / Nipponbare [ammonium (OsAnt1::AlaAT (1/7); 0....	0.08	0.25	1.22	0.479
OS-00126 (8) NIL(hwi1) / NIL(hwi1) [temperature (NIL(Hwi1); shift 20°C to 30°C; 3d) ...	0.07	0.25	1.22	0.511
OS-00037 (36) Zhenshan 97 / MH63 [GA3 (Zhenshan 97) / GA3 (MH63)]	0.47	0.25	1.19	0.013
OS-00121 (10) ammonium (OsAnt1::AlaAT (1/7); 0.5mM (NH4)2SO4; shoot) / ammo...	0.10	0.25	1.21	0.378
OS-00062 (118) RIL154 / Zhenshan 97	0.10	0.25	1.22	0.406
OS-00061 (40) IR24 / Nipponbare [X. oryzae pv. oryzae (IR24; PXO99AME5) / X. oryza	0.15	0.25	1.20	0.254
OS-00128 (7) MRP 5401 / PMS 80 [leaf]	0.09	0.24	1.21	0.412
OS-00126 (3) temperature (NIL(hwi1); shift 20°C to 30°C; 7d) / temperature (NIL(hwi...	0.15	0.24	1.19	0.252
OS-00102 (11) K354 / C418 [cold (K354; 2h) / cold (C418; 2h)]	0.15	0.24	1.18	0.227
OS-00070 (7) X. oryzae pv. oryzae (Huahui 1; JXOI) / untreated leaf samples (Huahui 1)	0.17	0.24	1.19	0.190
OS-00235 (16) Nagina 22 / Moroberekan [untreated pistil samples (Nagina 22) / untr...	0.23	0.24	1.18	0.103
OS-00111 (1) osbzip58-1 / Dongjin	0.02	0.23	1.23	0.848
OS-00222 (2) nitrogen (KNO3; NH4NO3; shoot) / nitrogen (KCl; shoot)	0.12	0.23	1.17	0.321
OS-00235 (15) Nagina 22 / Moroberekan [untreated anther samples (Nagina 22) / un...	0.09	0.23	1.21	0.401
OS-00127 (1) MH63 / Zhenshan 97	0.15	0.23	1.18	0.235
OS-00103 (6) cold (IR29; 4°C for 48h; 29°C for 24h) / untreated leaf samples (IR29)	0.08	0.23	1.20	0.423
OS-00091 (1) phosphorus (low Pi for 6h) / untreated seedling samples (sufficient Pi)	0.23	0.23	1.17	0.098
OS-00084 (4) M. oryzae (FR13) / mock treated leaf samples	0.12	0.23	1.18	0.300
OS-00221 (18) PL177 / IR64 [untreated shoot samples (PL177) / untreated shoot sa...	0.11	0.22	1.18	0.334
OS-00004 (4) trans-zeatin (120min; root) / mock treated root samples (120min)	0.13	0.22	1.17	0.251
OS-00061 (43) IR24 / Nipponbare [X. oryzae pv. oryzicola (IR24) / X. oryzae pv. oryzicol.	0.23	0.22	1.17	0.097
OS-00233 (1) drought (Douradoagulha) / untreated panicle samples (Douradoagulha)	0.17	0.22	1.17	0.175
OS-00277 (4) X. oryzae pv. oryzae (BXO1003) / mock treated leaf central vein samples	0.21	0.22	1.16	0.116
OS-00277 (3) X. oryzae pv. oryzae (BXO1002) / X. oryzae pv. oryzae (BXO4	0.17	0.22	1.16	0.176
OS-00032 (4) salt tolerant RILs / salt sensitive RILs [untreated seedling samples (s...	0.14	0.22	1.16	0.227
OS-00270 (5) Moroberekan / IR64 [drought (Moroberekan; FTSW 0.2) / drought (IR64;...	0.21	0.22	1.16	0.112
OS-00022 (1) Nip.GM / Nipponbare	0.27	0.21	1.16	0.057
OS-00130 (3) K83 / Jijing88 [alkali (K83) / alkali (Jijing88)]	0.32	0.21	1.16	0.032
OS-00061 (39) IR24 / Nipponbare [X. oryzae pv. oryzae (IR24; PXO99AME2) / X. oryza	0.25	0.21	1.16	0.068
OS-00238 (12) collar development (flag leaf; S2) / collar development (3rd leaf; S2)	0.08	0.21	1.15	0.437
OS-00068 (9) IRAT109 / Zhenshan 97 [drought (IRAT109; leaf RWC 83-88%) / droug...	0.14	0.21	1.15	0.213
OS-00123 (2) dehydration (Dongjin) / untreated root samples (Dongjin)	0.08	0.21	1.16	0.421
OS-00039 (2) drought (Bala) / untreated Bala leaf samples	0.21	0.21	1.16	0.102
OS-00128 (8) MRP 5401 / PMS 80 [roots]	0.26	0.21	1.16	0.056
OS-00278 (3) gs1;2 / Nipponbare [tiller development (gs1;2; V3) / tiller development (...	0.17	0.21	1.15	0.159
OS-00279 (2) fst (cyn) / Junambyeo [untreated panicle samples (fst (cyn); before flow...	0.11	0.21	1.15	0.296
OS-00036 (6) iron; phosphorus (no Fe and P for 10d; shoot) / untreated shoot sampl...	0.10	0.21	1.15	0.317
OS-00072 (3) TP-Pi54-15 / Taipei 309 [M. grisea (TP-Pi54-15) / M. grisea (Taipei 309)]	0.33	0.21	1.16	0.026
OS-00061 (9) X. oryzae pv. oryzae (IR24; PXO99AME2) / X. oryzae pv. oryzae (IR24; P:	0.16	0.21	1.15	0.169
OS-00103 (5) cold (IR29; 4°C for 48h; 29°C for 24h) / cold (IR29; 4°C for 48h)	0.07	0.20	1.18	0.446
OS-00278 (2) tiller development (Nipponbare; V4) / tiller development (Nipponbare; V...	0.17	0.20	1.15	0.151
OS-00237 (6) X. oryzae pv. oryzae (Rb49; 24hpi) / mock treated leaf samples (Rb49; ...	0.05	0.20	1.14	0.572
OS-00091 (2) phosphorus (low Pi for 24h) / untreated seedling samples (sufficient Pi)	0.10	0.20	1.15	0.306
OS-00102 (12) K354 / C418 [cold (K354; 6h) / cold (C418; 6h)]	0.13	0.20	1.15	0.213
OS-00068 (1) drought (IRAT109; leaf RWC 65-69%) / untreated flag leaf samples (IR...	0.09	0.20	1.15	0.367
OS-00233 (19) Huhan3 / Ematayin [drought (Huhan3) / drought (Ematayin)]	0.19	0.20	1.15	0.108
OS-00235 (12) Nagina 22 / Moroberekan [drought; heat (Nagina 22; anther; 3x6h) / dr...	0.22	0.19	1.14	0.070
OS-00098 (6) OsAnt1::AlaAT (3/8) / Nipponbare [shoot]	0.22	0.19	1.14	0.069
OS-00121 (12) ammonium (OsAnt1::AlaAT (1/7); 2mM (NH4)2SO4; shoot) / ammoni...	0.16	0.19	1.14	0.137
OS-00221 (17) PL177 / IR64 [untreated root samples (PL177) / untreated root sampl...	0.07	0.19	1.16	0.404
OS-00028 (3) slr1 (k1001) / Taichung 65	0.14	0.19	1.14	0.175
OS-00017 (2) S58S / Zhonghua11	0.08	0.19	1.13	0.395
OS-00233 (16) Huhan3 / Nipponbare [untreated panicle samples (Huhan3) / untreat...	0.17	0.19	1.14	0.118
OS-00037 (5) dark (MH63; radicle) / light grown radicle samples (MH63)	0.04	0.19	1.16	0.640
OS-00017 (1) S59R / Zhonghua11	0.07	0.18	1.13	0.436
OS-00109 (6) Pusa 1121 / Pusa 1342	0.20	0.18	1.13	0.077
OS-00100 (5) Jamaica / C Bao [leaf]	0.06	0.18	1.14	0.478
OS-00128 (5) MRP 5401 / PMS 80 [flag leaf]	0.11	0.18	1.13	0.241
OS-00232 (1) N. lugens (Rathu Heenati; 6hpi) / untreated shoot samples (Rathu He...	0.05	0.18	1.13	0.505
OS-00059 (1) chr729 / Hwayoung	0.25	0.18	1.13	0.036
OS-00008 (2) dehydration (3h) / untreated seedling samples (3h)	0.11	0.17	1.13	0.232
OS-00121 (26) OsAnt1::AlaAT (3/8) / Nipponbare [ammonium (OsAnt1::AlaAT (3/8); 0....	0.14	0.17	1.13	0.167
OS-00062 (117) RIL154 / MH63	0.04	0.17	1.15	0.602
OS-00046 (4) Nagina 22 / IR64 [untreated plant samples (Nagina 22) / untreated pla...	0.09	0.17	1.12	0.311
OS-00280 (4) TGMS-Co27 / Hejiang 19 [temperature (TGMS-Co27; 28°C) / temperat...	0.10	0.17	1.13	0.255
OS-00020 (4) KT95-418 / WuYun3 [RSV (KT95-418) / RSV (WuYun3)]	0.07	0.17	1.13	0.390
OS-00225 (6) Dhagaddeshi / IR20 [dehydration (Dhagaddeshi; 6h) / dehydration (IR...	0.09	0.17	1.13	0.309
OS-00280 (3) TGMS-Co27 / Hejiang 19 [temperature (TGMS-Co27; 20°C) / temperat...	0.11	0.17	1.12	0.237
OS-00025 (2) salt (Pokkali) / untreated root samples (Pokkali)	0.13	0.17	1.12	0.170
OS-00093 (1) long day (pistil and stamen primordia formation) / long day (glume pri...	0.15	0.17	1.12	0.120
OS-00102 (6) cold (K354; 2h) / untreated shoot samples (K354)	0.09	0.17	1.12	0.300
OS-00232 (13) Rathu Heenati / Taichung Native-1 [N. lugens (Rathu Heenati; 6hpi) / ...	0.06	0.17	1.14	0.425
OS-00045 (2) M. oryzae (4dpi) / mock treated root samples (4dpi)	0.03	0.17	1.10	0.646
OS-00022 (3) ScFv / Bengal	0.11	0.17	1.12	0.213
OS-00052 (3) imbibition (aerobic; 3h) / imbibition (aerobic; 1h)	0.06	0.17	1.14	0.457
OS-00233 (17) Huhan3 / Milt1444 [drought (Huhan3) / drought (Milt1444)]	0.09	0.17	1.12	0.276
OS-00014 (3) Bala / Azucena [root impedance (Bala; contact) / root impedance (Azuc...	0.15	0.16	1.12	0.117
OS-00037 (55) Zhenshan 97 / MH63 [untreated seedling samples (Zhenshan 97) / u...	0.07	0.16	1.12	0.384
OS-00239 (4) phosphorus (Pusa Basmati 1; 1µM Pi; shoot) / phosphorus (Pusa Bas...	0.07	0.16	1.11	0.394
OS-00108 (2) imbibition (4h; endosperm) / endosperm samples from dry seeds	0.12	0.16	1.11	0.191
OS-00070 (11) X. oryzae pv. oryzicola (Huahui 1) / untreated leaf samples (Huahui 1)	0.13	0.16	1.12	0.156
OS-00024 (1) heat (3h) / untreated seedling samples (3h)	0.06	0.16	1.13	0.410
OS-00042 (3) M202(Sub1) / M202 [submergence (M202(Sub1)) / submergence (M20...	0.13	0.16	1.12	0.164
OS-00234 (14) LYP9 / PA64s [optimal fertilization (LYP9; root tip) / optimal fertilization...	0.08	0.16	1.11	0.298
OS-00046 (1) drought (IR64) / untreated plant samples (IR64)	0.07	0.16	1.11	0.337
OS-00070 (8) X. oryzae pv. oryzae (Huahui 1; PXO99) / untreated leaf samples (Huah...	0.12	0.16	1.11	0.177
OS-00277 (5) X. oryzae pv. oryzae (BXO1003) / X. oryzae pv. oryzae (BXO4	0.08	0.15	1.10	0.285
OS-00036 (4) iron (no Fe for 10d; shoot) / untreated shoot samples (sufficient Fe an...	0.04	0.15	1.11	0.509
OS-00035 (28) pollination (5min; stigma) / untreated stigma samples (before pollina...	0.10	0.15	1.11	0.220
OS-00238 (8) collar development (3rd leaf; S4) / collar development (3rd leaf; S2)	0.02	0.15	1.11	0.691
OS-00237 (8) Rb49 / Mudanjiang 8 [mock treated leaf samples (Rb49; 4hpi) / mock t...	0.04	0.15	1.10	0.529
OS-00232 (12) wounding (Taichung Native-1; 24h) / untreated shoot samples (Taich...	0.45	0.15	1.11	<0.001
OS-00070 (12) X. oryzae pv. oryzicola (MH63) / untreated leaf samples (MH63)	0.04	0.15	1.13	0.550
OS-00047 (1) pollen development (bicellular) / pollen development (microspore)	0.12	0.15	1.11	0.148
OS-00119 (3) JA (3h) / mock treated seedling samples (3h)	0.06	0.15	1.10	0.402
OS-00091 (3) phosphorus (low Pi for 48h) / untreated seedling samples (sufficient Pi)	0.10	0.15	1.11	0.211
OS-00035 (1) anther development (bicellular pollen) / anther development (microspo...	0.05	0.14	1.12	0.471
OS-00055 (27) IRBB7 / IRBB5 [X. oryzae pv. oryzae (IRBB7; 0hpi) / X. oryzae pv. oryza	0.03	0.14	1.11	0.607
OS-00061 (36) IR24 / Nipponbare [X. oryzae pv. oryzae (IR24; PXO99A) / X. oryzae pv.	0.04	0.14	1.09	0.538
OS-00105 (3) X. oryzae pv. oryzae (IRBB21; 24hpi) / mock treated leaf samples (IRBB...	0.09	0.14	1.10	0.241
OS-00109 (3) aromatic RILs / Pusa 1342	0.13	0.14	1.10	0.114
OS-00102 (2) cold (C418; 6h) / untreated shoot samples (C418)	0.05	0.14	1.10	0.478
OS-00037 (20) panicle development (MH63; 40-50mm) / panicle development (MH6...	0.07	0.14	1.10	0.296
OS-00221 (7) salt (PL177; ABA; root) / salt (PL177; root)	0.07	0.14	1.11	0.288
OS-00238 (1) collar development (2nd leaf; S2) / collar development (3rd leaf; S2)	0.03	0.14	1.09	0.628
OS-00232 (2) N. lugens (Rathu Heenati; 6hpi) / wounding (Rathu Heenati; 6h)	0.03	0.14	1.10	0.610
OS-00098 (5) OsAnt1::AlaAT (3/8) / Nipponbare [roots]	0.09	0.14	1.10	0.202
OS-00237 (13) Rb49 / Mudanjiang 8 [X. oryzae pv. oryzae (Rb49; 24hpi) / X. oryzae pv..	0.04	0.13	1.10	0.513
OS-00235 (6) drought; heat (Nagina 22; anther; 3x6h) / heat (Nagina 22; anther; 3x6h)	0.08	0.13	1.09	0.269
OS-00068 (5) drought (Zhenshan 97; leaf RWC 74-78%) / untreated flag leaf samples ...	0.05	0.13	1.09	0.494
OS-00238 (2) collar development (2nd leaf; S4) / collar development (2nd leaf; S2)	0.05	0.13	1.09	0.419
OS-00239 (3) phosphorus (Pusa Basmati 1; 1µM Pi; root) / phosphorus (Pusa Basm...	0.07	0.13	1.09	0.268
OS-00232 (10) wounding (Rathu Heenati; 24h) / untreated shoot samples (Rathu He...	0.08	0.13	1.09	0.252
OS-00130 (4) K83 / Jijing88 [untreated shoot samples (K83) / untreated shoot sampl...	0.03	0.13	1.11	0.570
OS-00036 (7) phosphorus (no P for 10d; root) / iron; phosphorus (no Fe and P for 10...	0.10	0.13	1.09	0.169
OS-00061 (18) X. oryzae pv. oryzae (Nipponbare; PXO86) / X. oryzae pv. oryzae (Nippo.	0.04	0.13	1.09	0.505
OS-00055 (23) IRBB7 / IRBB5 [X. oryzae pv. oryzae (IRBB7; 3hpi) / X. oryzae pv. oryza	0.05	0.13	1.10	0.436
OS-00076 (1) KMD / Xiushui 11	0.04	0.12	1.10	0.464
OS-00238 (21) collar development (flag leaf; S6) / collar development (flag leaf; S2)	0.04	0.12	1.09	0.503
OS-00061 (13) X. oryzae pv. oryzae (IR24; PXO99AME7) / X. oryzae pv. oryzae (IR24; F	0.04	0.12	1.09	0.496
OS-00287 (1) salt (1h) / untreated shoot samples (1h)	0.09	0.12	1.09	0.184
OS-00096 (3) 35S::PSTOL1 (20) / IR64-null [phosphorus (35S::PSTOL1 (20); 60kg/h...	0.05	0.12	1.08	0.393
OS-00023 (4) FL478 / IR29 [untreated root samples (FL478) / untreated root sample...	0.05	0.12	1.09	0.356
OS-00102 (14) K354 / C418 [cold (K354; 24h) / cold (C418; 24h)]	0.03	0.12	1.11	0.581
OS-00048 (8) diurnal (8:00am; day 2; leaf) / diurnal (8:00am; day 1; leaf)	0.03	0.12	1.09	0.542
OS-00103 (18) Li-Jiang-Xin-Tuan-Hei-Gu / IR29 [untreated leaf samples (LTH) / untr...	0.04	0.12	1.09	0.455
OS-00058 (1) lemmata / Gui-630 x Taiwanjing	0.04	0.12	1.08	0.499
OS-00238 (3) collar development (2nd leaf; S4) / collar development (3rd leaf; S4)	0.02	0.12	1.07	0.686
OS-00288 (1) simulated drought (ewst1) / untreated leaf samples (ewst1)	0.07	0.12	1.09	0.279
OS-00061 (37) IR24 / Nipponbare [X. oryzae pv. oryzae (IR24; PXO99A) / X. oryzae pv.	0.04	0.12	1.09	0.461
OS-00233 (20) Huhan3 / Ematayin [untreated panicle samples (Huhan3) / untreated ...	0.06	0.12	1.08	0.299
OS-00241 (3) S. meliloti (alive; 5dpi) / S. meliloti (dead; 5dpi)	0.05	0.11	1.08	0.348
OS-00101 (3) potassium (no K for 6h) / untreated root samples (sufficient K; 6h)	0.04	0.11	1.08	0.457
OS-00068 (8) IRAT109 / Zhenshan 97 [drought (IRAT109; leaf RWC 74-78%) / droug...	0.05	0.11	1.08	0.325
OS-00233 (8) Milt1444 / Nipponbare [untreated panicle samples (Milt1444) / untreat...	0.05	0.11	1.08	0.336
OS-00267 (3) osmotic (DR5) / untreated seedling samples (DR5)	0.03	0.11	1.08	0.483
OS-00278 (4) gs1;2 / Nipponbare [tiller development (gs1;2; V4) / tiller development (...	0.03	0.11	1.09	0.515
OS-00061 (24) X. oryzae pv. oryzae (Nipponbare; PXO99AME2) / X. oryzae pv. oryzae (	0.04	0.11	1.08	0.389
OS-00070 (5) X. campestris pv. vesicatoria (Huahui 1) / untreated leaf samples (Hua...	0.05	0.11	1.08	0.356
OS-00128 (1) PR 319 / PMS 80 [flag leaf]	0.04	0.11	1.07	0.467
OS-00121 (36) OsAnt1::AlaAT (3/8) / OsAnt1::AlaAT (1/7) [ammonium (OsAnt1::AlaAT ...	0.05	0.11	1.07	0.326
OS-00238 (19) collar development (flag leaf; S6) / collar development (2nd leaf; S6)	0.02	0.10	1.08	0.579
OS-00037 (6) dark (MH63; shoot) / light grown seedling shoot samples (MH63)	0.02	0.10	1.09	0.664
OS-00234 (10) LYP9 / 93-11 [optimal fertilization (LYP9; root tip) / optimal fertilization ...	0.01	0.10	1.03	0.746
OS-00221 (13) PL177 / IR64 [salt (PL177; ABA; root) / salt (IR64; ABA; root)]	0.02	0.10	1.07	0.573
OS-00278 (1) tiller development (gs1;2; V4) / tiller development (gs1;2; V3)	0.03	0.10	1.08	0.550
OS-00018 (11) Nipponbare / IAC 165 [S. hermonthica (Nipponbare; 4dpi) / S. hermo...	0.03	0.10	1.08	0.537
OS-00235 (8) drought; heat (Nagina 22; pistil; 1x6h) / untreated pistil samples (Nagi...	0.04	0.10	1.07	0.428
OS-00030 (1) CODA-4 / Pusa Basmati 1	0.04	0.10	1.08	0.395
OS-00039 (1) drought (Azucena) / untreated Azucena leaf samples	0.03	0.10	1.07	0.446
OS-00281 (3) osrbohA / Dongjin [drought (osrbohA) / drought (Dongjin)]	0.03	0.10	1.08	0.552
OS-00037 (26) temperature (MH63; 31°C; 15dai) / untreated calli samples (MH63; 2...	0.00	0.10	1.00	0.899
OS-00238 (6) collar development (2nd leaf; S6) / collar development (2nd leaf; S2)	0.02	0.10	1.07	0.558
OS-00096 (4) 35S::PSTOL1 (20) / IR64-null [phosphorus (35S::PSTOL1 (20); no P fe...	0.02	0.09	1.06	0.634
OS-00233 (15) Huhan3 / Nipponbare [drought (Huhan3) / drought (Nipponbare)]	0.04	0.09	1.07	0.404
OS-00052 (1) imbibition (aerobic; 1h) / embryo samples from dry seeds	0.04	0.09	1.06	0.342
OS-00061 (15) X. oryzae pv. oryzae (IR24; T7174) / X. oryzae pv. oryzae (IR24; PXO99	0.03	0.09	1.06	0.490
OS-00035 (3) anther development (leptotene) / anther development (S-G2)	0.03	0.09	1.06	0.464
OS-00061 (38) IR24 / Nipponbare [X. oryzae pv. oryzae (IR24; PXO99AME1) / X. oryza	0.02	0.09	1.06	0.597
OS-00119 (4) salicylic acid (3h) / mock treated seedling samples (3h)	0.02	0.09	1.06	0.602
OS-00028 (1) gid1-3 / Taichung 65	0.07	0.09	1.06	0.172
OS-00070 (6) X. campestris pv. vesicatoria (MH63) / untreated leaf samples (MH63)	0.03	0.09	1.07	0.472
OS-00284 (1) GR-14 / Wuxianjing 9	0.00	0.09	-1.11	0.898
OS-00072 (2) M. grisea (TP-Pi54-15) / mock treated leaf samples (TP-Pi54-15)	0.04	0.09	1.06	0.322
OS-00040 (3) Cr(VI) (24h) / untreated root samples	0.02	0.09	1.07	0.645
OS-00117 (1) serf1 / Nipponbare	0.01	0.09	1.07	0.678
OS-00037 (8) dark (Zhenshan 97; shoot) / light grown seedling shoot samples (Zhe...	0.07	0.09	1.06	0.137
OS-00239 (6) Dular / Pusa Basmati 1 [phosphorus (Dular; 1µM Pi; shoot) / phosphor...	0.03	0.08	1.06	0.410
OS-00241 (2) S. meliloti (alive; 2dpi) / S. meliloti (dead; 2dpi)	0.02	0.08	1.06	0.587
OS-00068 (6) drought (Zhenshan 97; leaf RWC 83-88%) / untreated flag leaf sample...	0.01	0.08	1.06	0.709
OS-00121 (2) ammonium (Nipponbare; 0.5mM (NH4)2SO4; root) / ammonium (Nipp...	0.06	0.08	1.06	0.179
OS-00040 (4) Pb (24h) / untreated root samples	0.03	0.08	1.06	0.410
OS-00224 (2) drought (seedling stage) / untreated leaf samples (seedling stage)	0.01	0.08	1.07	0.816
OS-00109 (1) aromatic RILs / non-aromatic RILs	0.05	0.08	1.06	0.221
OS-00041 (6) drought (root; tillering) / untreated root samples (tillering)	0.02	0.08	1.06	0.650
OS-00073 (2) N. lugens (Rathu Heenati; 24hpi) / untreated stem samples (Rathu He...	0.02	0.08	1.06	0.532
OS-00031 (1) Pusa 1266 / Pusa Basmati 1	0.03	0.08	1.06	0.389
OS-00283 (2) IAA (crl1; 4h) / untreated crown samples (crl1)	0.03	0.08	1.06	0.400
OS-00028 (2) gid2-1 / Taichung 65	0.04	0.08	1.06	0.347
OS-00055 (22) IRBB7 / IR24 [X. oryzae pv. oryzae (IRBB7; 0hpi) / X. oryzae pv. oryzae (	0.01	0.08	1.06	0.730
OS-00096 (2) phosphorus (IR64-null; no P fertilizer) / phosphorus (IR64-null; 60kg/h...	0.01	0.08	1.06	0.737
OS-00106 (5) cold (24h) / untreated leaf samples	0.02	0.08	1.06	0.529
OS-00221 (16) PL177 / IR64 [salt (PL177; shoot) / salt (IR64; shoot)]	0.02	0.08	1.05	0.617
OS-00034 (4) X. oryzae pv. oryzae (24hpi) / mock treated shoot samples (24hpi)	0.01	0.08	1.05	0.665
OS-00233 (18) Huhan3 / Milt1444 [untreated panicle samples (Huhan3) / untreated ...	0.02	0.08	1.05	0.540
OS-00285 (2) M. oryzae (24hpi) / mock treated leaf samples (24hpi)	0.02	0.08	1.05	0.606
OS-00062 (187) MH63 / Zhenshan 97	0.02	0.08	1.06	0.617
OS-00011 (2) M. grisea (4dpi) / mock treated leaf samples (4dpi)	0.05	0.08	1.05	0.219
OS-00238 (11) collar development (flag leaf; S2) / collar development (2nd leaf; S2)	0.02	0.08	1.05	0.620
OS-00001 (1) salt (FL478) / untreated growing point samples (FL478)	0.03	0.07	1.06	0.394
OS-00062 (16) RIL014 / Zhenshan 97	0.01	0.07	1.07	0.766
OS-00128 (10) MRP 5401 / PR 319 [flag leaf]	0.02	0.07	1.05	0.538
OS-00221 (9) salt (PL177; ABA; shoot) / salt (PL177; shoot)	0.01	0.07	1.06	0.646
OS-00060 (1) Senia-afp / Senia	0.04	0.07	1.05	0.287
OS-00275 (1) N. lugens (KMD2) / untreated shoot samples (KMD2)	0.01	0.07	1.06	0.629
OS-00093 (3) short day (pistil and stamen primordia formation) / long day (pistil and ...	0.01	0.07	1.07	0.776
OS-00061 (22) X. oryzae pv. oryzae (Nipponbare; PXO99AME1) / X. oryzae pv. oryzae (	0.01	0.07	1.05	0.668
OS-00233 (10) Ematayin / Nipponbare [untreated panicle samples (Ematayin) / untre...	0.03	0.07	1.05	0.422
OS-00234 (8) LYP9 / 93-11 [nutrient deficiency (LYP9; root tip) / nutrient deficiency (9...	0.04	0.07	1.05	0.275
OS-00084 (2) M. oryzae (BR32) / mock treated leaf samples	0.01	0.07	1.06	0.744
OS-00063 (1) anther development (meiosis) / anther development (prior meiosis)	0.03	0.07	1.05	0.411
OS-00277 (1) X. oryzae pv. oryzae (BXO43) / mock treated leaf central vein samples	0.01	0.07	1.05	0.694
OS-00236 (4) arsenate (Nayanmoni) / untreated root samples (Nayanmoni)	0.01	0.07	1.05	0.611
OS-00037 (52) Zhenshan 97 / MH63 [untreated leaf sheath samples (Zhenshan 97) ...	0.01	0.07	1.04	0.752
OS-00098 (1) OsAnt1::AlaAT (1/7) / Nipponbare [roots]	0.03	0.07	1.05	0.329
OS-00280 (2) temperature (TGMS-Co27; 28°C) / temperature (TGMS-Co27; 20°C)	0.01	0.07	1.05	0.651
OS-00287 (3) salt (24h) / untreated shoot samples (24h)	0.01	0.07	1.03	0.755
OS-00061 (23) X. oryzae pv. oryzae (Nipponbare; PXO99AME2) / mock treated leaf sa...	0.01	0.07	1.04	0.600
OS-00280 (1) temperature (Hejiang 19; 28°C) / temperature (Hejiang 19; 20°C)	0.01	0.07	1.04	0.590
OS-00124 (1) OXJMJ705 / Zhonghua11	0.01	0.06	1.05	0.739
OS-00026 (1) RK16331-13 / FOX3	0.01	0.06	1.04	0.731
OS-00084 (1) M. grisea (BR29) / mock treated leaf samples	0.01	0.06	1.04	0.634
OS-00233 (6) drought (Nipponbare) / untreated panicle samples (Nipponbare)	0.02	0.06	1.04	0.415
OS-00121 (16) ammonium (OsAnt1::AlaAT (3/8); 0.5mM (NH4)2SO4; shoot) / ammo...	0.03	0.06	1.05	0.325
OS-00119 (2) ACC (3h) / mock treated seedling samples (3h)	0.01	0.06	1.04	0.708
OS-00020 (3) KT95-418 / WuYun3 [non-infected aeral tissue samples (KT95-418) / n...	0.01	0.06	1.05	0.701
OS-00098 (2) OsAnt1::AlaAT (1/7) / Nipponbare [shoot]	0.01	0.06	1.04	0.596
OS-00025 (4) IR63731 / Pokkali [untreated root samples (IR63731) / untreated root s...	0.02	0.06	1.04	0.549
OS-00121 (9) ammonium (OsAnt1::AlaAT (1/7); 0.5mM (NH4)2SO4; shoot) / ammoni...	0.01	0.06	1.07	0.818
OS-00235 (9) heat (Moroberekan; anther; 3x6h) / untreated anther samples (Morober...	0.01	0.06	1.04	0.615
OS-00266 (2) LipA (120min) / mock treated leaf samples (120min)	0.01	0.06	1.04	0.591
OS-00109 (5) non-aromatic RILs / Pusa 1342	0.02	0.06	1.04	0.477
OS-00108 (4) imbibition (8h; endosperm) / endosperm samples from dry seeds	0.02	0.06	1.04	0.444
OS-00103 (12) cold (LTH; 4°C for 48h; 29°C for 24h) / untreated leaf samples (LTH)	0.01	0.06	1.04	0.735
OS-00285 (4) M. oryzae (72hpi) / mock treated leaf samples (72hpi)	0.01	0.06	1.04	0.705
OS-00070 (10) X. oryzae pv. oryzae (MH63; PXO99) / untreated leaf samples (MH63)	0.03	0.06	1.04	0.331
OS-00070 (20) Huahui 1 / MH63 [X. oryzae pv. oryzae (Huahui 1; JXOI) / X. oryzae pv.	0.01	0.05	1.05	0.756
OS-00236 (1) arsenate (BRG 12) / untreated root samples (BRG 12)	0.01	0.05	1.03	0.735
OS-00042 (4) M202(Sub1) / M202 [untreated shoot samples (M202(Sub1)) / untreate...	0.01	0.05	1.03	0.756
OS-00037 (43) Zhenshan 97 / MH63 [panicle development (Zhenshan 97; 10-15mm...	0.00	0.05	1.15	0.917
OS-00108 (6) imbibition (12h; endosperm) / endosperm samples from dry seeds	0.01	0.05	1.04	0.626
OS-00096 (1) phosphorus (35S::PSTOL1 (20); no P fertilizer) / phosphorus (35S::PS...	0.04	0.05	1.04	0.176
OS-00034 (2) X. oryzae pv. oryzae (4hpi) / mock treated shoot samples (4hpi)	0.01	0.05	1.05	0.745
OS-00121 (29) OsAnt1::AlaAT (3/8) / Nipponbare [ammonium (OsAnt1::AlaAT (3/8); 5...	0.01	0.05	1.04	0.559
OS-00037 (57) Zhenshan 97 / MH63 [untreated root samples (Zhenshan 97) / untr...	0.00	0.05	1.08	0.917
OS-00267 (1) osmotic (DR0) / untreated seedling samples (DR0)	0.00	0.05	1.03	0.812
OS-00129 (1) osmads6-1 / 9522	0.01	0.05	1.03	0.712
OS-00082 (2) O. oryzae (GMB4M) / untreated stem samples	0.01	0.05	1.03	0.530
OS-00102 (15) K354 / C418 [cold (K354; 48h) / cold (C418; 48h)]	0.01	0.05	1.03	0.736
OS-00035 (27) pollination (5min; ovary) / untreated ovary samples (before pollination)	0.00	0.05	1.02	0.841
OS-00123 (4) arid / Dongjin [untreated root samples (arid) / untreated root samples (...	0.01	0.05	1.03	0.743
OS-00073 (6) Rathu Heenati / Taichung Native-1 [N. lugens (Rathu Heenati; 24hpi) / ...	0.00	0.05	1.03	0.842
OS-00128 (9) MRP 5401 / PMS 80 [roots]	0.00	0.05	1.03	0.832
OS-00034 (15) X. oryzae pv. oryzicola (96hpi) / X. oryzae pv. oryzae (96hp	0.01	0.05	1.03	0.689
OS-00048 (11) diurnal (8:00pm; day 2; flag leaf) / diurnal (8:00am; day 2; flag leaf)	0.01	0.05	1.03	0.526
OS-00107 (1) red (Nipponbare) / dark (Nipponbare)	0.01	0.05	1.03	0.645
OS-00037 (40) Zhenshan 97 / MH63 [NAA (Zhenshan 97) / NAA (MH63)]	0.00	0.05	1.05	0.865
OS-00061 (7) X. oryzae pv. oryzae (IR24; PXO99AME1) / X. oryzae pv. oryzae (IR24; P:	0.01	0.05	1.03	0.773
OS-00023 (2) FL478 / IR29 [salt (FL478) / salt (IR29)]	0.01	0.05	1.04	0.708
OS-00121 (23) OsAnt1::AlaAT (1/7) / Nipponbare [ammonium (OsAnt1::AlaAT (1/7); 5...	0.00	0.04	1.04	0.840
OS-00232 (9) wounding (Rathu Heenati; 6h) / untreated shoot samples (Rathu Heen...	0.00	0.04	1.03	0.869
OS-00039 (3) Bala / Azucena [drought (Bala) / drought (Azucena)]	0.01	0.04	1.03	0.697
OS-00062 (142) RIL178 / Zhenshan 97	0.00	0.04	1.05	0.874
OS-00073 (5) Rathu Heenati / Taichung Native-1 [N. lugens (Rathu Heenati; 8hpi) / N...	0.01	0.04	1.03	0.459
OS-00037 (23) panicle development (Zhenshan 97; 10-15mm) / panicle developme...	0.00	0.04	1.14	0.924
OS-00128 (4) PR 319 / PMS 80 [roots]	0.00	0.04	1.03	0.853
OS-00033 (1) 93-11 / Nipponbare	0.01	0.04	1.03	0.563
OS-00238 (4) collar development (2nd leaf; S5) / collar development (2nd leaf; S2)	0.00	0.04	1.02	0.769
OS-00121 (18) ammonium (OsAnt1::AlaAT (3/8); 2mM (NH4)2SO4; shoot) / ammoni...	0.00	0.04	1.03	0.767
OS-00276 (1) LipA / mock treated leaf samples	0.01	0.04	1.03	0.641
OS-00106 (3) cold (4h) / untreated leaf samples	0.00	0.04	1.03	0.698
OS-00237 (3) X. oryzae pv. oryzae (Mudanjiang 8; 24hpi) / mock treated leaf samples ...	0.00	0.04	1.02	0.879
OS-00073 (4) N. lugens (Taichung Native-1; 24hpi) / untreated stem samples (Taich...	0.00	0.04	1.03	0.863
OS-00128 (6) MRP 5401 / PR 319 [leaf]	0.01	0.04	1.02	0.700
OS-00041 (1) drought (leaf; booting) / untreated leaf samples (booting)	0.00	0.03	1.03	0.854
OS-00121 (17) ammonium (OsAnt1::AlaAT (3/8); 2mM (NH4)2SO4; root) / ammoniu...	0.01	0.03	1.02	0.703
OS-00061 (19) X. oryzae pv. oryzae (Nipponbare; PXO99A) / mock treated leaf sampl...	0.00	0.03	1.04	0.873
OS-00099 (1) OsRPL1-OE / Ishikari-shiroge	0.00	0.03	1.01	0.872
OS-00001 (3) FL478 / IR29	0.01	0.03	1.02	0.599
OS-00065 (1) gravistimulation (0.5h; lower part of shoot base) / gravistimulation (0.5...	0.00	0.03	1.02	0.738
OS-00040 (2) Cd (24h) / untreated root samples	0.00	0.03	1.02	0.729
OS-00226 (5) NIL-BPH9 / 93-11 [N. lugens (NIL-BPH9; 3hpi) / N. lugens (93-11; 3hpi)]	0.00	0.03	1.02	0.838
OS-00098 (4) OsAnt1::AlaAT (1/8) / Nipponbare [shoot]	0.00	0.03	1.02	0.787
OS-00036 (1) iron (no Fe for 10d; root) / iron; phosphorus (no Fe and P for 10d; root)	0.00	0.03	1.02	0.830
OS-00061 (21) X. oryzae pv. oryzae (Nipponbare; PXO99AME1) / mock treated leaf sa...	0.00	0.03	1.02	0.865
OS-00121 (11) ammonium (OsAnt1::AlaAT (1/7); 2mM (NH4)2SO4; root) / ammoniu...	0.00	0.03	1.01	0.870
OS-00236 (2) arsenate (BRG 15) / untreated root samples (BRG 15)	0.00	0.03	1.01	0.886
OS-00063 (2) anther development (microspores) / anther development (meiosis)	0.00	0.03	1.02	0.757
OS-00103 (13) Li-Jiang-Xin-Tuan-Hei-Gu / IR29 [cold (LTH; 4°C for 2h) / cold (IR29; 4...	0.00	0.03	1.03	0.863
OS-00062 (70) RIL089 / Zhenshan 97	0.00	0.03	1.02	0.755
OS-00034 (14) X. oryzae pv. oryzicola (96hpi) / mock treated shoot samples (96hpi)	0.00	0.03	1.01	0.787
OS-00001 (2) salt (IR29) / untreated growing point samples (IR29)	0.00	0.03	1.02	0.836
OS-00105 (8) IRBB21 / IR24 [X. oryzae pv. oryzae (IRBB21; 96hpi) / X. oryzae pv. oryza	0.00	0.03	1.02	0.751
OS-00121 (15) ammonium (OsAnt1::AlaAT (3/8); 0.5mM (NH4)2SO4; shoot) / ammo...	0.00	0.02	1.01	0.849
OS-00084 (3) M. oryzae (CL3.6.7) / mock treated leaf samples	0.00	0.02	1.01	0.842
OS-00281 (2) drought (osrbohA) / untreated leaf samples (osrbohA)	0.00	0.02	1.03	0.866
OS-00048 (4) diurnal (4:00pm; day 1; leaf) / diurnal (8:00am; day 1; leaf)	0.00	0.02	1.03	0.925
OS-00014 (1) root impedance (Azucena; buckled) / above layer root tip samples (Azu...	0.00	0.02	1.01	0.847
OS-00018 (5) S. hermonthica (Nipponbare; 4dpi) / mock treated root samples (Nipp...	0.00	0.02	1.02	0.878
OS-00103 (4) cold (IR29; 4°C for 48h) / untreated leaf samples (IR29)	0.00	0.02	1.01	0.853
OS-00041 (5) drought (root; panicle elongation) / untreated root samples (panicle el...	0.00	0.02	1.01	0.806
OS-00283 (5) crl1 / Taichung 65 [IAA (crl1; 2h) / IAA (Taichung 65; 2h)]	0.00	0.02	1.02	0.700
OS-00037 (38) Zhenshan 97 / MH63 [light grown radicle samples (Zhenshan 97) / li...	0.00	0.02	1.02	0.938
OS-00057 (1) X. oryzae pv. oryzae (BAI3) / mock treated leaf samples	0.00	0.02	1.01	0.838
OS-00226 (2) N. lugens (93-11; 6hpi) / untreated leaf sheath samples (93-11)	0.00	0.02	1.01	0.918
OS-00047 (3) pollen development (tricellular mature) / pollen development (tricellula...	0.00	0.02	1.01	0.750
OS-00104 (1) M. oryzae (IRBL18) / mock treated IRBL18 leaf samples	0.00	0.02	1.01	0.633
OS-00034 (12) X. oryzae pv. oryzicola (24hpi) / mock treated shoot samples (24hpi)	0.00	0.02	1.02	0.924
OS-00267 (11) DR5 / DR0 [osmotic (DR5) / osmotic (DR0)]	0.00	0.02	1.02	0.894
OS-00034 (1) X. oryzae pv. oryzae (2hpi) / mock treated shoot samples (2hpi)	0.00	0.02	1.01	0.911
OS-00034 (8) X. oryzae pv. oryzicola (4hpi) / mock treated shoot samples (4hpi)	0.00	0.02	1.02	0.896
OS-00034 (11) X. oryzae pv. oryzicola (8hpi) / X. oryzae pv. oryzae (8hp	0.00	0.02	1.01	0.896
OS-00095 (2) X. oryzae pv. oryzae (Nipponbare; BAI3 hrcC-) / X. oryzae pv. oryzae (Nip	0.00	0.01	1.00	0.941
OS-00124 (2) jmj705 / Hwayoung	0.00	0.01	1.01	0.895
OS-00037 (45) Zhenshan 97 / MH63 [panicle development (Zhenshan 97; heading) /...	0.00	0.01	1.01	0.944
OS-00062 (100) RIL134 / Zhenshan 97	0.00	0.01	1.01	0.922
OS-00121 (33) OsAnt1::AlaAT (3/8) / OsAnt1::AlaAT (1/7) [ammonium (OsAnt1::AlaAT ...	0.00	0.01	1.01	0.647
OS-00051 (8) anoxia (N2 for 30h) / anoxia (N2 for 1h)	0.00	0.01	1.01	0.927
OS-00120 (1) Huahui 1 / MH63	0.00	0.01	1.01	0.895
OS-00235 (11) Nagina 22 / Moroberekan [drought; heat (Nagina 22; anther; 1x6h) / dr...	0.00	0.01	1.01	0.925
OS-00267 (8) OX1 / OX0 [untreated seedling samples (OX1) / untreated seedling sa...	0.00	0.01	1.03	0.972
OS-00069 (3) drought (Kitaake) / untreated flag leaf samples (Kitaake)	0.00	0.01	1.01	0.934
OS-00121 (6) ammonium (Nipponbare; 2mM (NH4)2SO4; shoot) / ammonium (Nipp...	0.00	0.01	1.03	0.970
OS-00288 (4) ewst1 / Nagina 22 [untreated leaf samples (ewst1) / untreated leaf sa...	0.00	0.01	1.00	0.946
OS-00061 (2) X. oryzae pv. oryzae (IR24; PXO86) / X. oryzae pv. oryzae (IR24; PXO99	0.00	0.01	1.01	0.963
OS-00270 (7) Moroberekan / IR64 [untreated leaf elongation zone samples (Morober...	0.00	0.01	1.01	0.849
OS-00238 (16) collar development (flag leaf; S5) / collar development (2nd leaf; S5)	0.00	0.01	1.01	0.893
OS-00128 (13) MRP 5401 / PR 319 [roots]	0.00	0.01	1.01	0.970
OS-00121 (35) OsAnt1::AlaAT (3/8) / OsAnt1::AlaAT (1/7) [ammonium (OsAnt1::AlaAT ...	0.00	0.01	-1.01	0.972
OS-00126 (9) NIL(Hwi1) / NIL(hwi1) [temperature (NIL(Hwi1); shift 20°C to 30°C; 7d) ...	0.00	0.01	1.01	0.981
OS-00105 (5) IRBB21 / IR24 [untreated leaf samples (IRBB21; 24hpi) / mock trea...	0.00	0.00	-1.01	0.988
OS-00075 (1) IL50-7 / KMR3 [flag leaf]	0.00	0.00	-1.00	0.976
OS-00062 (140) RIL173 / Zhenshan 97	0.00	0.00	1.00	0.983
OS-00236 (3) arsenate (CN1646-5) / untreated root samples (CN1646-5)	0.00	0.00	-1.00	0.987
OS-00023 (2) salt (IR29) / untreated root samples (IR29)	0.00	0.00	1.00	0.964
OS-00042 (1) submergence (M202(Sub1)) / untreated shoot samples (M202(Sub1))	0.00	0.00	1.00	0.959
OS-00065 (2) gravistimulation (0.5h; lower part of shoot base) / untreated shoot bas...	0.00	0.00	-1.00	0.990
OS-00073 (7) Rathu Heenati / Taichung Native-1 [untreated stem samples (Rathu H...	0.00	0.00	-1.00	0.990
OS-00027 (1) miR156 OX / Zhonghua11 [untreated 3rd leaf samples (miR156 OX) / ...	0.00	0.00	1.00	0.987
OS-00070 (14) Huahui 1 / MH63 [heat (Huahui 1) / heat (MH63)]	0.00	0.00	1.00	1.000
OS-00121 (8) ammonium (OsAnt1::AlaAT (1/7); 0.5mM (NH4)2SO4; root) / ammoniu...	0.00	0.00	-1.00	0.997
OS-00075 (2) IL50-7 / KMR3 [panicle]	0.00	0.00	-1.00	0.979
OS-00062 (15) RIL014 / MH63	0.00	0.00	1.01	0.995
OS-00040 (1) arsenate (24h) / untreated root samples	0.00	0.00	-1.00	0.973
OS-00226 (7) NIL-BPH9 / 93-11 [untreated leaf sheath samples (NIL-BPH9) / untreat...	0.00	0.00	-1.01	0.985
OS-00048 (10) diurnal (8:00pm; day 1; leaf) / diurnal (8:00am; day 1; leaf)	0.00	0.00	1.00	0.984
OS-00235 (4) drought; heat (Moroberekan; pistil; 1x6h) / untreated pistil samples (Mo...	0.00	0.00	-1.00	0.977
OS-00232 (16) Rathu Heenati / Taichung Native-1 [untreated shoot samples (Rathu ...	0.00	0.00	-1.00	0.953
OS-00018 (9) Nipponbare / IAC 165 [mock treated root samples (Nipponbare; 11dpi)...	0.00	0.00	-1.00	0.928
OS-00062 (92) RIL125 / Zhenshan 97	0.00	-0.01	-1.00	0.954
OS-00035 (5) anther development (S-G2) / anther development (HAC)	0.00	-0.01	-1.01	0.970
OS-00051 (6) anoxia (N2 for 27h) / anoxia (N2 for 1h)	0.00	-0.01	-1.00	0.966
OS-00070 (9) X. oryzae pv. oryzae (MH63; JXOI) / untreated leaf samples (MH63)	0.00	-0.01	-1.00	0.929
OS-00065 (6) gravistimulation (6h; upper part of shoot base) / untreated shoot base ...	0.00	-0.01	-1.01	0.935
OS-00275 (3) KMD2 / Xiushui 11 [N. lugens (KMD2) / N. lugens (Xiushui 11)]	0.00	-0.01	1.00	0.947
OS-00037 (30) Zhenshan 97 / MH63 [callus regeneration (Zhenshan 97; 5d) / callus ...	0.00	-0.01	-1.02	0.971
OS-00267 (2) osmotic (DR3) / untreated seedling samples (DR3)	0.00	-0.01	-1.02	0.978
OS-00226 (4) N. lugens (NIL-BPH9; 6hpi) / untreated leaf sheath samples (NIL-BPH9)	0.00	-0.01	-1.00	0.947
OS-00077 (1) coi1-13 / Nipponbare	0.00	-0.01	-1.01	0.822
OS-00061 (11) X. oryzae pv. oryzae (IR24; PXO99AME5) / X. oryzae pv. oryzae (IR24; F	0.00	-0.01	-1.00	0.950
OS-00121 (31) OsAnt1::AlaAT (3/8) / OsAnt1::AlaAT (1/7) [ammonium (OsAnt1::AlaAT ...	0.00	-0.01	-1.02	0.948
OS-00074 (3) A. tumefaciens (Nipponbare; 12h) / untreated calli samples (Nipponba...	0.00	-0.02	-1.01	0.818
OS-00062 (110) RIL147 / Zhenshan 97	0.00	-0.02	-1.01	0.889
OS-00101 (1) potassium (no K for 3d) / untreated root samples (sufficient K; 3d)	0.00	-0.02	-1.02	0.828
OS-00064 (1) pollen development (bicellular) / pollen development (microspore)	0.00	-0.02	-1.01	0.751
OS-00057 (2) X. oryzae pv. oryzae (BAI3Talc) / mock treated leaf samples	0.00	-0.02	-1.02	0.803
OS-00056 (1) Fukei 71 / Fujiminori [parenchyma]	0.00	-0.02	-1.01	0.847
OS-00104 (4) IRBL18 / Li-Jiang-Xin-Tuan-Hei-Gu [M. oryzae (IRBL18) / M. oryzae (LT...	0.00	-0.02	-1.01	0.712
OS-00055 (8) X. oryzae pv. oryzae (IRBB5; 24hpi) / X. oryzae pv. oryzae (IRBB5; 0hp	0.00	-0.02	-1.02	0.902
OS-00106 (1) cold (0.5h) / untreated leaf samples	0.00	-0.02	-1.01	0.699
OS-00232 (11) wounding (Taichung Native-1; 6h) / untreated shoot samples (Taichu...	0.00	-0.02	-1.05	0.948
OS-00034 (5) X. oryzae pv. oryzae (96hpi) / mock treated shoot samples (96hpi)	0.00	-0.02	-1.01	0.887
OS-00121 (14) ammonium (OsAnt1::AlaAT (3/8); 0.5mM (NH4)2SO4; root) / ammoni...	0.00	-0.02	-1.01	0.854
OS-00267 (7) OX1 / OX0 [osmotic (OX1) / osmotic (OX0)]	0.00	-0.02	-1.02	0.917
OS-00035 (29) pollination (40-50min; pistil) / pollination (15-25min; pistil)	0.00	-0.02	-1.02	0.868
OS-00128 (2) PR 319 / PMS 80 [leaf]	0.00	-0.02	-1.02	0.925
OS-00232 (18) Rathu Heenati / Taichung Native-1 [wounding (Rathu Heenati; 24h) / ...	0.00	-0.02	-1.01	0.769
OS-00004 (3) trans-zeatin (120min; leaf) / mock treated leaf samples (120min)	0.00	-0.02	-1.02	0.859
OS-00241 (4) S. meliloti (alive; 8dpi) / S. meliloti (dead; 8dpi)	0.01	-0.02	-1.02	0.390
OS-00285 (1) M. oryzae (12hpi) / mock treated leaf samples (12hpi)	0.00	-0.02	-1.02	0.832
OS-00267 (10) DR3 / DR0 [untreated seedling samples (DR3) / untreated seedling ...	0.00	-0.02	-1.00	0.950
OS-00091 (4) phosphorus (low Pi for 72h) / untreated seedling samples (sufficient Pi)	0.00	-0.02	-1.02	0.846
OS-00027 (2) miR156 OX / Zhonghua11 [untreated 6th leaf samples (miR156 OX) / ...	0.00	-0.03	-1.02	0.836
OS-00238 (20) collar development (flag leaf; S6) / collar development (3rd leaf; S6)	0.00	-0.03	-1.01	0.878
OS-00004 (1) trans-zeatin (30min; leaf) / mock treated leaf samples (30min)	0.00	-0.03	-1.02	0.798
OS-00013 (1) gif1 / Zhonghua11	0.00	-0.03	-1.04	0.938
OS-00234 (13) LYP9 / PA64s [optimal fertilization (LYP9; leaf) / optimal fertilization (P...	0.00	-0.03	-1.02	0.887
OS-00008 (3) salt (3h) / untreated seedling samples (3h)	0.00	-0.03	-1.02	0.748
OS-00106 (4) cold (8h) / untreated leaf samples	0.01	-0.03	-1.02	0.553
OS-00121 (7) ammonium (OsAnt1::AlaAT (1/7); 0.5mM (NH4)2SO4; root) / ammoniu...	0.00	-0.03	-1.01	0.858
OS-00090 (1) ila1 / Zhonghua11	0.00	-0.03	-1.01	0.941
OS-00238 (18) collar development (flag leaf; S5) / collar development (flag leaf; S2)	0.00	-0.03	-1.02	0.721
OS-00018 (1) S. hermonthica (IAC 165; 2dpi) / mock treated root samples (IAC 165; ...	0.00	-0.03	-1.02	0.759
OS-00233 (3) drought (Huhan3) / untreated panicle samples (Huhan3)	0.00	-0.03	-1.02	0.812
OS-00065 (3) gravistimulation (0.5h; upper part of shoot base) / untreated shoot bas...	0.00	-0.03	-1.02	0.819
OS-00237 (9) Rb49 / Mudanjiang 8 [mock treated leaf samples (Rb49; 24hpi) / mock...	0.00	-0.03	-1.02	0.933
OS-00095 (4) IR64 / Nipponbare [X. oryzae pv. oryzae (IR64; BAI3) / X. oryzae pv. oryza	0.00	-0.03	-1.03	0.878
OS-00062 (26) RIL019 / Zhenshan 97	0.00	-0.03	-1.01	0.880
OS-00236 (16) Nayanmoni / CN1646-5 [arsenate (Nayanmoni) / arsenate (CN1646-...	0.00	-0.03	-1.02	0.833
OS-00037 (39) Zhenshan 97 / MH63 [light grown seedling shoot samples (Zhensha...	0.03	-0.03	-1.02	0.138
OS-00102 (16) K354 / C418 [untreated shoot samples (K354) / untreated shoot sam...	0.00	-0.03	-1.03	0.867
OS-00062 (141) RIL178 / MH63	0.00	-0.03	-1.01	0.918
OS-00055 (1) X. oryzae pv. oryzae (IR24; 3hpi) / X. oryzae pv. oryzae (IR24; 0hp	0.01	-0.03	-1.02	0.679
OS-00233 (11) Ematayin / Milt1444 [drought (Ematayin) / drought (Milt1444)]	0.00	-0.03	-1.03	0.758
OS-00232 (7) N. lugens (Taichung Native-1; 24hpi) / untreated shoot samples (Taich...	0.00	-0.03	-1.01	0.862
OS-00226 (6) NIL-BPH9 / 93-11 [N. lugens (NIL-BPH9; 6hpi) / N. lugens (93-11; 6hpi)]	0.00	-0.03	-1.03	0.856
OS-00034 (9) X. oryzae pv. oryzicola (4hpi) / X. oryzae pv. oryzae (4hp	0.00	-0.03	-1.03	0.864
OS-00052 (6) imbibition (aerobic; 24h) / embryo samples from dry seeds	0.01	-0.04	-1.03	0.712
OS-00104 (7) IRBL22 / Li-Jiang-Xin-Tuan-Hei-Gu [mock treated IRBL22 leaf sample...	0.00	-0.04	-1.03	0.855
OS-00233 (13) Douradoagulha / Khaumeo [drought (Douradoagulha) / drought (Kha...	0.00	-0.04	-1.04	0.892
OS-00224 (3) drought (V4) / untreated leaf samples (V4)	0.01	-0.04	-1.02	0.410
OS-00035 (23) caryopsis development (top 3/4; 4dap) / caryopsis development (top ...	0.00	-0.04	-1.02	0.820
OS-00054 (4) 9804-Rxo1 / 9804 [X. oryzae pv. oryzicola (9804-Rxo1) / X. oryzae pv. ory	0.00	-0.04	-1.03	0.817
OS-00055 (16) IRBB5 / IR24 [X. oryzae pv. oryzae (IRBB5; 24hpi) / X. oryzae pv. oryzae	0.00	-0.04	-1.03	0.769
OS-00233 (12) Ematayin / Milt1444 [untreated panicle samples (Ematayin) / untreate...	0.01	-0.04	-1.03	0.725
OS-00057 (3) X. oryzae pv. oryzae (BAI3Talc) / X. oryzae pv. oryzae (BA	0.01	-0.04	-1.03	0.595
OS-00267 (12) DR5 / DR0 [untreated seedling samples (DR5) / untreated seedling ...	0.00	-0.04	-1.04	0.853
OS-00121 (19) OsAnt1::AlaAT (1/7) / Nipponbare [ammonium (OsAnt1::AlaAT (1/7); 0....	0.00	-0.04	-1.02	0.850
OS-00037 (14) GA3 (Zhenshan 97) / untreated seedling samples (Zhenshan 97)	0.00	-0.04	-1.05	0.873
OS-00234 (11) LYP9 / PA64s [nutrient deficiency (LYP9; leaf) / nutrient deficiency (PA...	0.01	-0.04	-1.03	0.641
OS-00109 (2) aromatic RILs / Pusa 1121	0.01	-0.04	-1.03	0.577
OS-00061 (20) X. oryzae pv. oryzae (Nipponbare; PXO99A) / mock treated leaf sampl...	0.00	-0.04	-1.03	0.767
OS-00105 (4) X. oryzae pv. oryzae (IRBB21; 96hpi) / mock treated leaf samples (IRBB...	0.01	-0.04	-1.03	0.695
OS-00062 (34) RIL027 / Zhenshan 97	0.01	-0.04	-1.03	0.597
OS-00048 (14) diurnal (12:00am; day 1; leaf) / diurnal (8:00am; day 1; leaf)	0.00	-0.04	-1.03	0.732
OS-00121 (34) OsAnt1::AlaAT (3/8) / OsAnt1::AlaAT (1/7) [ammonium (OsAnt1::AlaAT ...	0.01	-0.04	-1.03	0.741
OS-00234 (4) optimal fertilization (LYP9; root tip) / nutrient deficiency (LYP9; root tip)	0.01	-0.04	-1.03	0.585
OS-00030 (2) CODA-2 / Pusa Basmati 1	0.06	-0.05	-1.03	0.061
OS-00275 (2) N. lugens (Xiushui 11) / untreated shoot samples (Xiushui 11)	0.02	-0.05	-1.03	0.413
OS-00055 (4) X. oryzae pv. oryzae (IR24; 24hpi) / X. oryzae pv. oryzae (IR24; 0hp	0.01	-0.05	-1.05	0.720
OS-00126 (7) NIL(Hwi1) / NIL(hwi1) [temperature (NIL(Hwi1); 20°C) / temperature (N...	0.00	-0.05	-1.03	0.783
OS-00104 (6) IRBL22 / Li-Jiang-Xin-Tuan-Hei-Gu [M. oryzae (IRBL22) / M. oryzae (LT...	0.02	-0.05	-1.03	0.336
OS-00062 (69) RIL089 / MH63	0.01	-0.05	-1.04	0.789
OS-00034 (10) X. oryzae pv. oryzicola (8hpi) / mock treated shoot samples (8hpi)	0.01	-0.05	-1.04	0.607
OS-00125 (1) M. oryzae elicitors (2h) / mock treated leaf samples (2h)	0.01	-0.05	-1.03	0.669
OS-00092 (1) Epi-df (986083D) / 986083	0.01	-0.05	-1.03	0.518
OS-00037 (32) Zhenshan 97 / MH63 [dark (Zhenshan 97; shoot) / dark (MH63; shoot)]	0.00	-0.05	-1.05	0.833
OS-00082 (1) O. oryzae (GMB1) / untreated stem samples	0.01	-0.05	-1.04	0.591
OS-00062 (178) RIL215 / Zhenshan 97	0.01	-0.05	-1.03	0.677
OS-00037 (35) Zhenshan 97 / MH63 [endosperm development (Zhenshan 97; 21da...	0.00	-0.05	-1.03	0.849
OS-00282 (1) phr1 / Nipponbare	0.01	-0.05	-1.03	0.783
OS-00103 (17) Li-Jiang-Xin-Tuan-Hei-Gu / IR29 [cold (LTH; 4°C for 48h; 29°C for 24h...	0.00	-0.05	-1.06	0.850
OS-00062 (134) RIL164 / Zhenshan 97	0.01	-0.05	-1.04	0.585
OS-00234 (18) PA64s / 93-11 [optimal fertilization (PA64s; root tip) / optimal fertilizati...	0.00	-0.05	-1.08	0.870
OS-00121 (25) OsAnt1::AlaAT (3/8) / Nipponbare [ammonium (OsAnt1::AlaAT (3/8); 0....	0.01	-0.05	-1.04	0.666
OS-00062 (184) RIL236 / Zhenshan 97	0.01	-0.06	-1.04	0.577
OS-00121 (13) ammonium (OsAnt1::AlaAT (3/8); 0.5mM (NH4)2SO4; root) / ammoni...	0.01	-0.06	-1.04	0.649
OS-00006 (2) OsWRKY13-ox (D11UM7-2) / Mudanjiang 8	0.01	-0.06	-1.04	0.605
OS-00036 (3) iron (no Fe for 10d; shoot) / iron; phosphorus (no Fe and P for 10d; sh...	0.01	-0.06	-1.04	0.697
OS-00100 (2) CJF1 / C Bao [root]	0.02	-0.06	-1.04	0.457
OS-00037 (7) dark (Zhenshan 97; radicle) / light grown radicle samples (Zhenshan ...	0.01	-0.06	-1.05	0.791
OS-00034 (13) X. oryzae pv. oryzicola (24hpi) / X. oryzae pv. oryzae (24hp	0.01	-0.06	-1.03	0.804
OS-00070 (3) heat (Huahui 1) / untreated leaf samples (Huahui 1)	0.01	-0.06	-1.05	0.817
OS-00098 (3) OsAnt1::AlaAT (1/8) / Nipponbare [roots]	0.03	-0.06	-1.04	0.274
OS-00036 (8) phosphorus (no P for 10d; root) / untreated root samples (sufficient Fe ...	0.01	-0.06	-1.05	0.713
OS-00062 (99) RIL134 / MH63	0.01	-0.06	-1.05	0.776
OS-00055 (25) IRBB7 / IRBB5 [X. oryzae pv. oryzae (IRBB7; 12hpi) / X. oryzae pv. oryz	0.03	-0.06	-1.04	0.325
OS-00055 (17) IRBB5 / IR24 [X. oryzae pv. oryzae (IRBB5; 0hpi) / X. oryzae pv. oryzae (	0.01	-0.06	-1.03	0.713
OS-00238 (14) collar development (flag leaf; S4) / collar development (3rd leaf; S4)	0.01	-0.06	-1.06	0.822
OS-00237 (12) Rb49 / Mudanjiang 8 [X. oryzae pv. oryzae (Rb49; 4hpi) / X. oryzae pv. ...	0.01	-0.06	-1.04	0.761
OS-00034 (3) X. oryzae pv. oryzae (8hpi) / mock treated shoot samples (8hpi)	0.02	-0.07	-1.04	0.565
OS-00282 (2) phr3 / Nipponbare	0.02	-0.07	-1.05	0.567
OS-00070 (18) Huahui 1 / MH63 [untreated leaf samples (Huahui 1) / untreated leaf ...	0.01	-0.07	-1.04	0.688
OS-00065 (4) gravistimulation (6h; lower part of shoot base) / gravistimulation (6h; u...	0.03	-0.07	-1.05	0.390
OS-00037 (34) Zhenshan 97 / MH63 [endosperm development (Zhenshan 97; 14da...	0.01	-0.07	-1.04	0.706
OS-00095 (6) IRBB4 / IR64 [X. oryzae pv. oryzae (IRBB4; BAI3) / X. oryzae pv. oryzae (	0.01	-0.07	-1.05	0.626
OS-00277 (6) X. oryzae pv. oryzae (BXO1003) / X. oryzae pv. oryzae (BXO100	0.03	-0.07	-1.05	0.341
OS-00130 (2) alkali (K83) / untreated shoot samples (K83)	0.01	-0.07	-1.07	0.748
OS-00039 (4) Bala / Azucena [untreated Bala leaf samples / untreated Azucena leaf s...	0.02	-0.07	-1.05	0.582
OS-00061 (16) X. oryzae pv. oryzae (Nipponbare; PXO86) / mock treated leaf sample...	0.01	-0.07	-1.04	0.735
OS-00036 (10) phosphorus (no P for 10d; shoot) / untreated shoot samples (sufficie...	0.01	-0.07	-1.06	0.691
OS-00048 (15) diurnal (12:00am; day 2; flag leaf) / diurnal (8:00am; day 2; flag leaf)	0.03	-0.07	-1.05	0.434
OS-00233 (7) Milt1444 / Nipponbare [drought (Milt1444) / drought (Nipponbare)]	0.02	-0.07	-1.05	0.534
OS-00062 (139) RIL173 / MH63	0.01	-0.07	-1.06	0.719
OS-00023 (1) salt (FL478) / untreated root samples (FL478)	0.02	-0.07	-1.05	0.619
OS-00008 (1) cold (3h) / untreated seedling samples (3h)	0.04	-0.08	-1.06	0.277
OS-00025 (1) salt (IR63731) / untreated root samples (IR63731)	0.03	-0.08	-1.06	0.357
OS-00105 (6) IRBB21 / IR24 [mock treated leaf samples (IRBB21; 96hpi) / mock trea...	0.01	-0.08	-1.06	0.685
OS-00065 (5) gravistimulation (6h; lower part of shoot base) / untreated shoot base ...	0.03	-0.08	-1.06	0.467
OS-00045 (1) M. oryzae (2dpi) / mock treated root samples (2dpi)	0.01	-0.08	-1.06	0.751
OS-00052 (4) imbibition (aerobic; 12h) / embryo samples from dry seeds	0.02	-0.08	-1.06	0.583
OS-00128 (3) PR 319 / PMS 80 [roots]	0.15	-0.08	-1.06	0.013
OS-00267 (9) OX2 / OX0 [osmotic (OX2) / osmotic (OX0)]	0.01	-0.08	-1.06	0.718
OS-00121 (32) OsAnt1::AlaAT (3/8) / OsAnt1::AlaAT (1/7) [ammonium (OsAnt1::AlaAT ...	0.01	-0.08	-1.08	0.758
OS-00234 (2) optimal fertilization (93-11; root tip) / nutrient deficiency (93-11; root tip)	0.01	-0.08	-1.01	0.800
OS-00237 (2) X. oryzae pv. oryzae (Mudanjiang 8; 4hpi) / mock treated leaf samples (...	0.01	-0.08	-1.06	0.701
OS-00055 (26) IRBB7 / IRBB5 [X. oryzae pv. oryzae (IRBB7; 24hpi) / X. oryzae pv. oryz	0.03	-0.08	-1.06	0.387
OS-00055 (19) IRBB7 / IR24 [X. oryzae pv. oryzae (IRBB7; 6hpi) / X. oryzae pv. oryzae (	0.02	-0.08	-1.05	0.514
OS-00074 (4) A. tumefaciens (Nipponbare; 24h) / untreated calli samples (Nipponba...	0.03	-0.08	-1.06	0.384
OS-00102 (3) cold (C418; 12h) / untreated shoot samples (C418)	0.01	-0.08	-1.06	0.674
OS-00062 (91) RIL125 / MH63	0.01	-0.08	-1.06	0.678
OS-00233 (4) drought (Khaumeo) / untreated panicle samples (Khaumeo)	0.01	-0.08	-1.04	0.752
OS-00072 (4) TP-Pi54-15 / Taipei 309 [mock treated leaf samples (TP-Pi54-15) / mo...	0.03	-0.08	-1.06	0.448
OS-00267 (5) OX2 / OX0 [untreated seedling samples (OX2) / untreated seedling s...	0.17	-0.08	-1.06	0.009
OS-00061 (25) X. oryzae pv. oryzae (Nipponbare; PXO99AME5) / mock treated leaf sa...	0.02	-0.08	-1.06	0.530
OS-00061 (3) X. oryzae pv. oryzae (IR24; PXO86) / X. oryzae pv. oryzae (IR24; T717	0.02	-0.08	-1.06	0.566
OS-00048 (5) diurnal (4:00pm; day 2; flag leaf) / diurnal (8:00am; day 2; flag leaf)	0.04	-0.08	-1.06	0.307
OS-00062 (156) RIL191 / Zhenshan 97	0.02	-0.08	-1.06	0.560
OS-00267 (13) DR3 / DR0 [osmotic (DR3) / osmotic (DR0)]	0.02	-0.09	-1.06	0.645
OS-00106 (2) cold (2h) / untreated leaf samples	0.02	-0.09	-1.06	0.548
OS-00221 (6) salt (IR64; shoot) / untreated shoot samples (IR64)	0.02	-0.09	-1.06	0.651
OS-00062 (14) RIL013 / Zhenshan 97	0.03	-0.09	-1.06	0.514
OS-00226 (3) N. lugens (NIL-BPH9; 3hpi) / untreated leaf sheath samples (NIL-BPH9)	0.03	-0.09	-1.06	0.493
OS-00073 (1) N. lugens (Rathu Heenati; 8hpi) / untreated stem samples (Rathu Hee...	0.08	-0.09	-1.06	0.113
OS-00037 (48) Zhenshan 97 / MH63 [untreated caryopsis samples (Zhenshan 97; 3...	0.00	-0.09	-1.15	0.963
OS-00286 (2) CMP (24h) / mock treated cell culture samples (24h)	0.05	-0.09	-1.07	0.283
OS-00237 (4) X. oryzae pv. oryzae (Rb49; 2hpi) / mock treated leaf samples (Rb49; 2...	0.02	-0.09	-1.06	0.639
OS-00037 (21) panicle development (MH63; heading) / panicle development (MH63; ...	0.02	-0.09	-1.06	0.613
OS-00037 (49) Zhenshan 97 / MH63 [untreated flag leaf samples (Zhenshan 97; 14d...	0.01	-0.09	-1.09	0.822
OS-00070 (21) Huahui 1 / MH63 [X. oryzae pv. oryzae (Huahui 1; PXO99) / X. oryzae p.	0.05	-0.09	-1.06	0.277
OS-00103 (3) cold (IR29; 4°C for 24h) / untreated leaf samples (IR29)	0.02	-0.09	-1.05	0.677
OS-00062 (109) RIL147 / MH63	0.02	-0.09	-1.07	0.650
OS-00104 (3) M. oryzae (LTH) / mock treated LTH leaf samples	0.02	-0.09	-1.08	0.594
OS-00068 (10) IRAT109 / Zhenshan 97 [untreated flag leaf samples (IRAT109; leaf R...	0.02	-0.09	-1.07	0.686
OS-00062 (90) RIL118 / Zhenshan 97	0.04	-0.09	-1.07	0.384
OS-00270 (2) drought (IR64; FTSW 0.5) / untreated leaf elongation zone samples (IR...	0.04	-0.09	-1.07	0.357
OS-00005 (1) OsRR6-ox / Nipponbare/ev	0.06	-0.09	-1.07	0.256
OS-00095 (3) X. oryzae pv. oryzae (Nipponbare; MAI1) / X. oryzae pv. oryzae (Nipponb	0.02	-0.10	-1.08	0.594
OS-00070 (17) Huahui 1 / MH63 [untreated leaf samples (Huahui 1) / untreated leaf ...	0.05	-0.10	-1.07	0.304
OS-00108 (7) imbibition (16h; embryo) / embryo samples from dry seeds	0.02	-0.10	-1.07	0.587
OS-00034 (6) X. oryzae pv. oryzicola (2hpi) / mock treated shoot samples (2hpi)	0.04	-0.10	-1.07	0.383
OS-00015 (1) BAP (1+3h) / mock treated seedling samples (1+3h)	0.04	-0.10	-1.07	0.392
OS-00037 (3) callus regeneration (MH63; 5d) / callus growth (MH63; screening)	0.01	-0.10	-1.06	0.746
OS-00236 (5) Nayanmoni / CN1646-5 [untreated root samples (Nayanmoni) / untre...	0.03	-0.10	-1.08	0.517
OS-00236 (10) BRG 15 / BRG 12 [untreated root samples (BRG 15) / untreated root ...	0.01	-0.10	-1.08	0.723
OS-00221 (1) salt (IR64; root) / untreated root samples (IR64)	0.03	-0.10	-1.06	0.488
OS-00239 (1) phosphorus (Dular; 1µM Pi; root) / phosphorus (Dular; 320µM Pi; root)	0.03	-0.10	-1.07	0.469
OS-00032 (2) salt (salt tolerant RILs) / untreated seedling samples (salt tolerant RILs)	0.02	-0.10	-1.07	0.596
OS-00237 (11) Rb49 / Mudanjiang 8 [X. oryzae pv. oryzae (Rb49; 2hpi) / X. oryzae pv. .	0.02	-0.10	-1.07	0.621
OS-00095 (7) IRBB4 / Nipponbare [X. oryzae pv. oryzae (IRBB4; BAI3) / X. oryzae pv. o	0.02	-0.10	-1.08	0.611
OS-00103 (14) Li-Jiang-Xin-Tuan-Hei-Gu / IR29 [cold (LTH; 4°C for 8h) / cold (IR29; 4...	0.03	-0.10	-1.07	0.454
OS-00100 (1) CJF1 / C Bao [leaf]	0.04	-0.10	-1.08	0.430
OS-00037 (51) Zhenshan 97 / MH63 [untreated leaf blade samples (Zhenshan 97) / ...	0.02	-0.10	-1.06	0.680
OS-00108 (1) imbibition (4h; embryo) / embryo samples from dry seeds	0.02	-0.10	-1.07	0.575
OS-00061 (17) X. oryzae pv. oryzae (Nipponbare; PXO86) / X. oryzae pv. oryzae (Nippo.	0.02	-0.10	-1.08	0.690
OS-00104 (2) M. oryzae (IRBL22) / mock treated IRBL22 leaf samples	0.05	-0.10	-1.08	0.344
OS-00123 (1) dehydration (arid) / untreated root samples (arid)	0.03	-0.10	-1.07	0.542
OS-00233 (9) Ematayin / Nipponbare [drought (Ematayin) / drought (Nipponbare)]	0.09	-0.10	-1.08	0.136
OS-00102 (1) cold (C418; 2h) / untreated shoot samples (C418)	0.02	-0.11	-1.08	0.633
OS-00042 (2) submergence (M202) / untreated shoot samples (M202)	0.02	-0.11	-1.08	0.584
OS-00270 (3) drought (Moroberekan; FTSW 0.2) / untreated leaf elongation zone sa...	0.04	-0.11	-1.08	0.400
OS-00267 (6) osmotic (OX2) / untreated seedling samples (OX2)	0.04	-0.11	-1.07	0.384
OS-00062 (25) RIL019 / MH63	0.02	-0.11	-1.07	0.689
OS-00062 (30) RIL022 / Zhenshan 97	0.04	-0.11	-1.07	0.444
OS-00102 (13) K354 / C418 [cold (K354; 12h) / cold (C418; 12h)]	0.03	-0.11	-1.08	0.510
OS-00062 (52) RIL068 / Zhenshan 97	0.06	-0.11	-1.08	0.262
OS-00103 (1) cold (IR29; 4°C for 2h) / untreated leaf samples (IR29)	0.05	-0.11	-1.09	0.350
OS-00281 (4) osrbohA / Dongjin [untreated leaf samples (osrbohA) / untreated leaf s...	0.05	-0.11	-1.08	0.328
OS-00018 (7) Nipponbare / IAC 165 [mock treated root samples (Nipponbare; 2dpi) /...	0.02	-0.11	-1.06	0.723
OS-00233 (2) drought (Ematayin) / untreated panicle samples (Ematayin)	0.09	-0.11	-1.08	0.173
OS-00267 (4) osmotic (OX0) / untreated seedling samples (OX0)	0.03	-0.11	-1.07	0.558
OS-00034 (7) X. oryzae pv. oryzicola (2hpi) / X. oryzae pv. oryzae (2hp	0.05	-0.11	-1.09	0.387
OS-00006 (1) OsWRKY13-ox (D11UM1-1) / Mudanjiang 8	0.05	-0.11	-1.09	0.349
OS-00236 (6) BRG 12 / Nayanmoni [untreated root samples (BRG 12) / untreated ro...	0.03	-0.12	-1.08	0.569
OS-00011 (1) M. grisea (3dpi) / mock treated leaf samples (3dpi)	0.01	-0.12	-1.08	0.773
OS-00062 (154) RIL187 / Zhenshan 97	0.03	-0.12	-1.07	0.589
OS-00055 (21) IRBB7 / IR24 [X. oryzae pv. oryzae (IRBB7; 24hpi) / X. oryzae pv. oryzae	0.05	-0.12	-1.09	0.390
OS-00233 (5) drought (Milt1444) / untreated panicle samples (Milt1444)	0.05	-0.12	-1.08	0.407
OS-00061 (26) X. oryzae pv. oryzae (Nipponbare; PXO99AME5) / X. oryzae pv. oryzae (	0.03	-0.12	-1.10	0.571
OS-00062 (33) RIL027 / MH63	0.03	-0.12	-1.09	0.521
OS-00037 (59) Zhenshan 97 / MH63 [untreated stem samples (Zhenshan 97; bootin...	0.07	-0.12	-1.09	0.278
OS-00132 (1) sd37 / Zhongxian 3037	0.02	-0.12	-1.11	0.621
OS-00014 (2) root impedance (Azucena; contact) / above layer root tip samples (Azuc...	0.07	-0.12	-1.09	0.286
OS-00010 (1) OsSRT1RNAi / MH63	0.03	-0.12	-1.10	0.571
OS-00226 (1) N. lugens (93-11; 3hpi) / untreated leaf sheath samples (93-11)	0.03	-0.12	-1.09	0.518
OS-00015 (2) IAA (1+3h) / mock treated seedling samples (1+3h)	0.05	-0.12	-1.09	0.417
OS-00284 (3) HFL1 / Wuxiangjing 9	0.01	-0.12	-1.14	0.888
OS-00236 (9) BRG 15 / BRG 12 [arsenate (BRG 15) / arsenate (BRG 12)]	0.11	-0.12	-1.09	0.140
OS-00109 (4) non-aromatic RILs / Pusa 1121	0.11	-0.13	-1.09	0.134
OS-00232 (14) Rathu Heenati / Taichung Native-1 [N. lugens (Rathu Heenati; 24hpi) ...	0.02	-0.13	-1.09	0.651
OS-00055 (2) X. oryzae pv. oryzae (IR24; 6hpi) / X. oryzae pv. oryzae (IR24; 0hp	0.11	-0.13	-1.09	0.126
OS-00037 (13) GA3 (MH63) / untreated seedling samples (MH63)	0.03	-0.13	-1.11	0.579
OS-00062 (177) RIL215 / MH63	0.03	-0.13	-1.10	0.541
OS-00275 (4) KMD2 / Xiushui 11 [untreated shoot samples (KMD2) / untreated shoot...	0.13	-0.13	-1.09	0.091
OS-00238 (15) collar development (flag leaf; S4) / collar development (flag leaf; S2)	0.08	-0.13	-1.09	0.247
OS-00283 (3) IAA (Taichung 65; 2h) / untreated crown samples (Taichung 65)	0.06	-0.13	-1.09	0.337
OS-00052 (7) imbibition (aerobic; 24h) / imbibition (aerobic; 1h)	0.12	-0.13	-1.09	0.122
OS-00061 (27) X. oryzae pv. oryzae (Nipponbare; PXO99AME7) / mock treated leaf sa..	0.07	-0.13	-1.10	0.300
OS-00035 (26) pollination (5-7h; pistil) / pollination (40-50min; pistil)	0.05	-0.13	-1.09	0.416
OS-00062 (133) RIL164 / MH63	0.04	-0.13	-1.10	0.505
OS-00048 (1) diurnal (4:00am; day 2; flag leaf) / diurnal (8:00am; day 1; flag leaf)	0.06	-0.13	-1.10	0.370
OS-00238 (7) collar development (2nd leaf; S6) / collar development (3rd leaf; S6)	0.08	-0.13	-1.09	0.252
OS-00062 (183) RIL236 / MH63	0.04	-0.13	-1.10	0.502
OS-00073 (3) N. lugens (Taichung Native-1; 8hpi) / untreated stem samples (Taichu...	0.09	-0.13	-1.10	0.222
OS-00221 (5) salt (IR64; ABA; root) / salt (IR64; root)	0.04	-0.13	-1.09	0.500
OS-00236 (5) BRG 12 / Nayanmoni [arsenate (BRG 12) / arsenate (Nayanmoni)]	0.07	-0.13	-1.10	0.298
OS-00041 (2) drought (leaf; panicle elongation) / untreated leaf samples (panicle elo...	0.04	-0.13	-1.10	0.464
OS-00104 (5) IRBL18 / Li-Jiang-Xin-Tuan-Hei-Gu [mock treated IRBL18 leaf sample...	0.05	-0.13	-1.11	0.444
OS-00224 (1) drought (R4) / untreated panicle samples (R4)	0.07	-0.14	-1.10	0.288
OS-00102 (10) cold (K354; 48h) / untreated shoot samples (K354)	0.06	-0.14	-1.10	0.359
OS-00101 (2) potassium (no K for 5d) / untreated root samples (sufficient K; 5d)	0.07	-0.14	-1.10	0.286
OS-00285 (3) M. oryzae (48hpi) / mock treated leaf samples (48hpi)	0.15	-0.14	-1.10	0.090
OS-00062 (68) RIL087 / Zhenshan 97	0.11	-0.14	-1.10	0.160
OS-00238 (17) collar development (flag leaf; S5) / collar development (3rd leaf; S5)	0.11	-0.14	-1.10	0.166
OS-00103 (16) Li-Jiang-Xin-Tuan-Hei-Gu / IR29 [cold (LTH; 4°C for 48h) / cold (IR29; ...	0.06	-0.14	-1.10	0.370
OS-00016 (1) ClsA (12h) / mock treated leaf samples (12h)	0.07	-0.14	-1.11	0.319
OS-00061 (32) X. oryzae pv. oryzicola (Nipponbare) / mock treated leaf samples (Nip...	0.07	-0.14	-1.11	0.317
OS-00037 (41) Zhenshan 97 / MH63 [panicle development (Zhenshan 97; 1mm) / pa...	0.14	-0.14	-1.11	0.107
OS-00126 (10) NIL(Hwi1) / NIL(hwi1) [temperature (NIL(Hwi1); shift 20°C to 30°C; 15...	0.04	-0.14	-1.09	0.541
OS-00062 (166) RIL199 / Zhenshan 97	0.05	-0.15	-1.11	0.469
OS-00267 (5) osmotic (OX1) / untreated seedling samples (OX1)	0.03	-0.15	-1.12	0.667
OS-00235 (1) drought; heat (Moroberekan; anther; 1x6h) / untreated anther samples ...	0.12	-0.15	-1.11	0.145
OS-00105 (2) X. oryzae pv. oryzae (IR24; 96hpi) / mock treated leaf samples (IR24; 9...	0.06	-0.15	-1.12	0.414
OS-00103 (2) cold (IR29; 4°C for 8h) / untreated leaf samples (IR29)	0.06	-0.15	-1.11	0.370
OS-00238 (5) collar development (2nd leaf; S5) / collar development (3rd leaf; S5)	0.13	-0.15	-1.11	0.127
OS-00020 (1) RSV (KT95-418) / non-infected aeral tissue samples (KT95-418)	0.05	-0.15	-1.11	0.481
OS-00045 (3) M. oryzae (6dpi) / mock treated root samples (6dpi)	0.02	-0.15	-1.09	0.689
OS-00134 (2) OX-Ghd7HJ19 / Hejiang 19 [panicle]	0.17	-0.15	-1.11	0.072
OS-00070 (1) cold (Huahui 1) / untreated leaf samples (Huahui 1)	0.02	-0.15	-1.08	0.708
OS-00053 (1) 93-11 / Nipponbare	0.12	-0.15	-1.10	0.158
OS-00055 (18) IRBB7 / IR24 [X. oryzae pv. oryzae (IRBB7; 3hpi) / X. oryzae pv. oryzae (	0.06	-0.15	-1.10	0.378
OS-00047 (4) pollen germination / pollen development (tricellular mature)	0.06	-0.15	-1.10	0.380
OS-00068 (7) IRAT109 / Zhenshan 97 [drought (IRAT109; leaf RWC 65-69%) / droug...	0.03	-0.15	-1.12	0.594
OS-00232 (17) Rathu Heenati / Taichung Native-1 [wounding (Rathu Heenati; 6h) / w...	0.05	-0.15	-1.10	0.492
OS-00234 (3) optimal fertilization (LYP9; leaf) / nutrient deficiency (LYP9; leaf)	0.06	-0.15	-1.10	0.399
OS-00035 (25) pollination (5-7h; pistil) / pollination (15-25min; pistil)	0.07	-0.15	-1.11	0.332
OS-00037 (54) Zhenshan 97 / MH63 [untreated root samples (Zhenshan 97) / untrea...	0.06	-0.15	-1.12	0.432
OS-00118 (4) stamen development (stage 6) / stamen development (stage 2)	0.03	-0.15	-1.14	0.593
OS-00093 (2) short day (glume primordium differentiation) / long day (glume primord...	0.09	-0.15	-1.11	0.272
OS-00130 (1) alkali (Jijing88) / untreated shoot samples (Jijing88)	0.12	-0.15	-1.11	0.163
OS-00133 (2) spl5 / Zhefu802 [untreated leaf samples (spl5) / untreated leaf sample...	0.03	-0.15	-1.12	0.638
OS-00062 (42) RIL048 / Zhenshan 97	0.04	-0.15	-1.09	0.586
OS-00062 (66) RIL086 / Zhenshan 97	0.05	-0.15	-1.10	0.478
OS-00103 (15) Li-Jiang-Xin-Tuan-Hei-Gu / IR29 [cold (LTH; 4°C for 24h) / cold (IR29; ...	0.05	-0.16	-1.13	0.484
OS-00022 (4) Estrela mut. / Estrela A	0.05	-0.16	-1.11	0.506
OS-00232 (3) N. lugens (Rathu Heenati; 24hpi) / untreated shoot samples (Rathu H...	0.05	-0.16	-1.10	0.467
OS-00048 (6) diurnal (4:00pm; day 2; leaf) / diurnal (8:00am; day 2; leaf)	0.04	-0.16	-1.11	0.594
OS-00037 (50) Zhenshan 97 / MH63 [untreated flag leaf samples (Zhenshan 97; boo...	0.04	-0.16	-1.13	0.547
OS-00032 (3) salt tolerant RILs / salt sensitive RILs [salt (salt tolerant RILs) / salt (sa...	0.04	-0.16	-1.12	0.581
OS-00102 (8) cold (K354; 12h) / untreated shoot samples (K354)	0.07	-0.16	-1.11	0.364
OS-00036 (2) iron (no Fe for 10d; root) / untreated root samples (sufficient Fe and P)	0.05	-0.16	-1.12	0.458
OS-00062 (155) RIL191 / MH63	0.05	-0.16	-1.12	0.474
OS-00221 (10) salt (PL177; ABA; shoot) / untreated shoot samples (PL177)	0.05	-0.16	-1.12	0.510
OS-00283 (4) IAA (Taichung 65; 4h) / untreated crown samples (Taichung 65)	0.10	-0.16	-1.12	0.257
OS-00061 (28) X. oryzae pv. oryzae (Nipponbare; PXO99AME7) / X. oryzae pv. oryzae (	0.06	-0.16	-1.14	0.425
OS-00062 (13) RIL013 / MH63	0.06	-0.16	-1.12	0.452
OS-00236 (7) BRG 12 / CN1646-5 [arsenate (BRG 12) / arsenate (CN1646-5)]	0.13	-0.16	-1.12	0.169
OS-00234 (5) optimal fertilization (PA64s; leaf) / nutrient deficiency (PA64s; leaf)	0.11	-0.16	-1.12	0.214
OS-00062 (78) RIL103 / Zhenshan 97	0.13	-0.16	-1.12	0.166
OS-00221 (15) PL177 / IR64 [salt (PL177; root) / salt (IR64; root)]	0.10	-0.17	-1.13	0.259
OS-00071 (1) abl1 / Zhonghua11 [ABA (abl1) / ABA (Zhonghua11)]	0.07	-0.17	-1.12	0.375
OS-00035 (19) caryopsis development (top 3/4; 2dap) / caryopsis development (top ...	0.06	-0.17	-1.12	0.428
OS-00266 (1) LipA (30min) / mock treated leaf samples (30min)	0.06	-0.17	-1.13	0.449
OS-00097 (1) dao / Nipponbare	0.11	-0.17	-1.13	0.217
OS-00062 (186) RIL241 / Zhenshan 97	0.16	-0.17	-1.13	0.107
OS-00062 (4) RIL005 / Zhenshan 97	0.05	-0.17	-1.10	0.514
OS-00019 (1) dst / Zhonghua11	0.44	-0.17	-1.13	0.003
OS-00046 (3) Nagina 22 / IR64 [drought (Nagina 22) / drought (IR64)]	0.12	-0.17	-1.13	0.199
OS-00062 (89) RIL118 / MH63	0.07	-0.17	-1.13	0.401
OS-00052 (5) imbibition (aerobic; 12h) / imbibition (aerobic; 1h)	0.11	-0.17	-1.12	0.215
OS-00102 (9) cold (K354; 24h) / untreated shoot samples (K354)	0.05	-0.17	-1.11	0.487
OS-00118 (1) stamen development (stage 3) / stamen development (stage 2)	0.05	-0.17	-1.15	0.540
OS-00055 (15) IRBB5 / IR24 [X. oryzae pv. oryzae (IRBB5; 12hpi) / X. oryzae pv. oryzae	0.08	-0.17	-1.14	0.366
OS-00062 (80) RIL105 / Zhenshan 97	0.12	-0.17	-1.13	0.210
OS-00066 (1) lc1 / Zhonghua11	0.10	-0.17	-1.13	0.248
OS-00070 (19) Huahui 1 / MH63 [X. campestris pv. vesicatoria (Huahui 1) / X. campe...	0.10	-0.17	-1.13	0.276
OS-00062 (72) RIL090 / Zhenshan 97	0.17	-0.18	-1.13	0.104
OS-00070 (22) Huahui 1 / MH63 [X. oryzae pv. oryzicola (Huahui 1) / X. oryzae pv. oryzi	0.05	-0.18	-1.15	0.497
OS-00288 (3) ewst1 / Nagina 22 [simulated drought (ewst1) / simulated drought (Na...	0.09	-0.18	-1.13	0.322
OS-00070 (13) Huahui 1 / MH63 [cold (Huahui 1) / cold (MH63)]	0.04	-0.18	-1.09	0.607
OS-00232 (6) N. lugens (Taichung Native-1; 6hpi) / wounding (Taichung Native-1; 6h)	0.13	-0.18	-1.13	0.187
OS-00125 (2) M. oryzae elicitors (30min) / mock treated leaf samples (30min)	0.12	-0.18	-1.14	0.207
OS-00121 (22) OsAnt1::AlaAT (1/7) / Nipponbare [ammonium (OsAnt1::AlaAT (1/7); 2...	0.06	-0.18	-1.16	0.482
OS-00282 (5) phr1/2/3 / phr3	0.19	-0.18	-1.13	0.090
OS-00232 (8) N. lugens (Taichung Native-1; 24hpi) / wounding (Taichung Native-1; 2...	0.08	-0.18	-1.12	0.375
OS-00238 (13) collar development (flag leaf; S4) / collar development (2nd leaf; S4)	0.15	-0.18	-1.13	0.157
OS-00286 (3) fenclorim (4h) / mock treated cell culture samples (4h)	0.20	-0.18	-1.13	0.077
OS-00003 (2) arsenate (Bala) / untreated root samples (Bala)	0.19	-0.18	-1.14	0.096
OS-00046 (2) drought (Nagina 22) / untreated plant samples (Nagina 22)	0.13	-0.18	-1.14	0.184
OS-00062 (12) RIL012 / Zhenshan 97	0.17	-0.18	-1.14	0.123
OS-00062 (29) RIL022 / MH63	0.07	-0.18	-1.14	0.409
OS-00025 (3) IR63731 / Pokkali [salt (IR63731) / salt (Pokkali)]	0.19	-0.18	-1.14	0.093
OS-00281 (1) drought (Dongjin) / untreated leaf samples (Dongjin)	0.13	-0.18	-1.14	0.194
OS-00062 (51) RIL068 / MH63	0.09	-0.18	-1.15	0.348
OS-00036 (5) iron; phosphorus (no Fe and P for 10d; root) / untreated root samples (...	0.09	-0.19	-1.14	0.319
OS-00062 (124) RIL159 / Zhenshan 97	0.19	-0.19	-1.14	0.100
OS-00062 (120) RIL155 / Zhenshan 97	0.15	-0.19	-1.14	0.156
OS-00062 (22) RIL017 / Zhenshan 97	0.18	-0.19	-1.14	0.106
OS-00113 (1) cea62 / Nipponbare	0.12	-0.19	-1.14	0.226
OS-00062 (136) RIL165 / Zhenshan 97	0.15	-0.19	-1.14	0.166
OS-00055 (3) X. oryzae pv. oryzae (IR24; 12hpi) / X. oryzae pv. oryzae (IR24; 0hp	0.09	-0.19	-1.13	0.334
OS-00070 (15) Huahui 1 / MH63 [untreated leaf samples (Huahui 1) / untreated leaf ...	0.26	-0.19	-1.14	0.043
OS-00062 (153) RIL187 / MH63	0.06	-0.19	-1.14	0.487
OS-00035 (24) ovary development (top 3/4; 0dap) / ovary development (bottom 1/4; 0...	0.02	-0.19	-1.33	0.786
OS-00282 (4) phr1/2/3 / phr1	0.10	-0.19	-1.15	0.304
OS-00037 (42) Zhenshan 97 / MH63 [panicle development (Zhenshan 97; 3-5mm) / ...	0.13	-0.20	-1.14	0.223
OS-00061 (29) X. oryzae pv. oryzae (Nipponbare; T7174) / mock treated leaf samples...	0.15	-0.20	-1.15	0.162
OS-00018 (12) Nipponbare / IAC 165 [S. hermonthica (Nipponbare; 11dpi) / S. herm...	0.12	-0.20	-1.15	0.248
OS-00035 (12) caryopsis development (bottom 1/4; 1dap) / ovary development (botto...	0.02	-0.20	-1.37	0.768
OS-00070 (2) cold (MH63) / untreated leaf samples (MH63)	0.11	-0.20	-1.15	0.287
OS-00232 (5) N. lugens (Taichung Native-1; 6hpi) / untreated shoot samples (Taichu...	0.05	-0.20	-1.19	0.540
OS-00103 (7) cold (LTH; 4°C for 2h) / untreated leaf samples (LTH)	0.10	-0.20	-1.14	0.314
OS-00234 (12) LYP9 / PA64s [nutrient deficiency (LYP9; root tip) / nutrient deficiency (...	0.26	-0.20	-1.15	0.056
OS-00072 (1) M. grisea (Taipei 309) / mock treated leaf samples (Taipei 309)	0.21	-0.20	-1.15	0.093
OS-00048 (2) diurnal (4:00am; day 2; leaf) / diurnal (8:00am; day 1; leaf)	0.21	-0.20	-1.15	0.092
OS-00037 (56) Zhenshan 97 / MH63 [untreated seedling samples (Zhenshan 97) / u...	0.09	-0.21	-1.19	0.348
OS-00037 (19) panicle development (MH63; 10-15mm) / panicle development (MH6...	0.16	-0.21	-1.15	0.171
OS-00054 (2) X. oryzae pv. oryzicola (9804-Rxo1) / untreated flag leaf samples (9804...	0.25	-0.21	-1.15	0.061
OS-00108 (5) imbibition (12h; embryo) / embryo samples from dry seeds	0.20	-0.21	-1.16	0.113
OS-00062 (162) RIL197 / Zhenshan 97	0.14	-0.21	-1.15	0.209
OS-00241 (1) S. meliloti (alive; 1dpi) / S. meliloti (dead; 1dpi)	0.08	-0.21	-1.14	0.412
OS-00237 (1) X. oryzae pv. oryzae (Mudanjiang 8; 2hpi) / mock treated leaf samples (...	0.13	-0.21	-1.16	0.233
OS-00222 (1) nitrogen (KNO3; NH4NO3; root) / nitrogen (KCl; root)	0.36	-0.21	-1.16	0.019
OS-00284 (4) HFL1 / GR-14	0.03	-0.21	-1.03	0.723
OS-00270 (6) Moroberekan / IR64 [drought (Moroberekan; FTSW 0.5) / drought (IR64;...	0.17	-0.21	-1.16	0.157
OS-00232 (15) Rathu Heenati / Taichung Native-1 [untreated shoot samples (Rathu ...	0.05	-0.21	-1.19	0.563
OS-00236 (8) BRG 12 / CN1646-5 [untreated root samples (BRG 12) / untreated root...	0.10	-0.21	-1.16	0.330
OS-00236 (12) BRG 15 / Nayanmoni [untreated root samples (BRG 15) / untreated r...	0.10	-0.22	-1.15	0.333
OS-00062 (67) RIL087 / MH63	0.12	-0.22	-1.17	0.284
OS-00048 (3) diurnal (4:00pm; day 1; flag leaf) / diurnal (8:00am; day 1; flag leaf)	0.11	-0.22	-1.16	0.301
OS-00102 (5) cold (C418; 48h) / untreated shoot samples (C418)	0.11	-0.22	-1.17	0.303
OS-00062 (32) RIL026 / Zhenshan 97	0.17	-0.22	-1.16	0.170
OS-00037 (16) NAA (MH63) / untreated seedling samples (MH63)	0.10	-0.22	-1.18	0.355
OS-00237 (7) Rb49 / Mudanjiang 8 [mock treated leaf samples (Rb49; 2hpi) / mock t...	0.15	-0.22	-1.17	0.196
OS-00062 (46) RIL054 / Zhenshan 97	0.25	-0.22	-1.17	0.071
OS-00062 (165) RIL199 / MH63	0.09	-0.22	-1.16	0.407
OS-00121 (27) OsAnt1::AlaAT (3/8) / Nipponbare [ammonium (OsAnt1::AlaAT (3/8); 2...	0.17	-0.22	-1.17	0.179
OS-00062 (180) RIL219 / Zhenshan 97	0.14	-0.22	-1.16	0.225
OS-00121 (1) ammonium (Nipponbare; 0.5mM (NH4)2SO4; root) / ammonium (Nipp...	0.17	-0.22	-1.17	0.181
OS-00037 (31) Zhenshan 97 / MH63 [dark (Zhenshan 97; radicle) / dark (MH63; radic...	0.06	-0.22	-1.19	0.547
OS-00035 (2) anther development (diplotene-tetrad) / anther development (zygotene-...	0.22	-0.22	-1.17	0.100
OS-00121 (28) OsAnt1::AlaAT (3/8) / Nipponbare [ammonium (OsAnt1::AlaAT (3/8); 2...	0.08	-0.22	-1.19	0.427
OS-00283 (6) crl1 / Taichung 65 [IAA (crl1; 4h) / IAA (Taichung 65; 4h)]	0.22	-0.23	-1.17	0.104
OS-00133 (1) spl5 / Zhefu802 [untreated leaf samples (spl5) / untreated leaf sample...	0.08	-0.23	-1.19	0.440
OS-00062 (41) RIL048 / MH63	0.07	-0.23	-1.15	0.490
OS-00061 (30) X. oryzae pv. oryzae (Nipponbare; T7174) / X. oryzae pv. oryzae (Nippo.	0.12	-0.23	-1.19	0.286
OS-00062 (158) RIL193 / Zhenshan 97	0.11	-0.23	-1.16	0.347
OS-00062 (65) RIL086 / MH63	0.09	-0.23	-1.17	0.411
OS-00221 (2) salt (IR64; ABA; root) / untreated root samples (IR64)	0.17	-0.23	-1.16	0.175
OS-00070 (16) Huahui 1 / MH63 [untreated leaf samples (Huahui 1) / untreated leaf ...	0.10	-0.23	-1.16	0.386
OS-00131 (1) osrdr1 / OsRDR1-sibling	0.24	-0.23	-1.18	0.090
OS-00115 (1) potassium (no K for 5d) / untreated seedling samples (sufficient K; 10d)	0.18	-0.23	-1.17	0.173
OS-00237 (10) Rb49 / Mudanjiang 8 [untreated leaf samples (Rb49; 0h) / untreated l...	0.16	-0.23	-1.17	0.204
OS-00018 (3) S. hermonthica (IAC 165; 11dpi) / mock treated root samples (IAC 165;...	0.18	-0.23	-1.17	0.175
OS-00221 (12) salt (PL177; shoot) / untreated shoot samples (PL177)	0.14	-0.23	-1.19	0.254
OS-00121 (21) OsAnt1::AlaAT (1/7) / Nipponbare [ammonium (OsAnt1::AlaAT (1/7); 2...	0.27	-0.23	-1.18	0.073
OS-00037 (53) Zhenshan 97 / MH63 [untreated lemma and palea samples (Zhensh...	0.23	-0.23	-1.18	0.106
OS-00055 (20) IRBB7 / IR24 [X. oryzae pv. oryzae (IRBB7; 12hpi) / X. oryzae pv. oryzae	0.14	-0.24	-1.19	0.244
OS-00103 (10) cold (LTH; 4°C for 48h) / untreated leaf samples (LTH)	0.16	-0.24	-1.17	0.216
OS-00062 (130) RIL162 / Zhenshan 97	0.09	-0.24	-1.15	0.399
OS-00037 (9) endosperm development (MH63; 14dap) / endosperm development (...	0.11	-0.24	-1.19	0.332
OS-00062 (77) RIL103 / MH63	0.14	-0.24	-1.19	0.259
OS-00239 (2) phosphorus (Dular; 1µM Pi; shoot) / phosphorus (Dular; 320µM Pi; sh...	0.14	-0.24	-1.20	0.261
OS-00055 (12) X. oryzae pv. oryzae (IRBB7; 24hpi) / X. oryzae pv. oryzae (IRBB7; 0hp	0.12	-0.24	-1.19	0.321
OS-00235 (3) drought; heat (Moroberekan; anther; 3x6h) / untreated anther samples ...	0.29	-0.24	-1.18	0.066
OS-00235 (14) Nagina 22 / Moroberekan [heat (Nagina 22; anther; 3x6h) / heat (Moro...	0.21	-0.24	-1.18	0.132
OS-00055 (5) X. oryzae pv. oryzae (IRBB5; 3hpi) / X. oryzae pv. oryzae (IRBB5; 0hp	0.17	-0.24	-1.20	0.193
OS-00062 (10) RIL009 / Zhenshan 97	0.11	-0.24	-1.16	0.346
OS-00062 (185) RIL241 / MH63	0.15	-0.25	-1.19	0.234
OS-00062 (3) RIL005 / MH63	0.09	-0.25	-1.17	0.435
OS-00282 (3) phr1/2/3 / Nipponbare	0.25	-0.25	-1.19	0.099
OS-00051 (5) anoxia (N2 for 24h; air for 6h) / anoxia (N2 for 24h)	0.20	-0.25	-1.19	0.154
OS-00062 (79) RIL105 / MH63	0.14	-0.25	-1.19	0.268
OS-00085 (1) X. oryzae pv. oryzae (Y73; PXO341) / mock treated leaf samples (Y73)	0.08	-0.25	-1.19	0.481
OS-00062 (71) RIL090 / MH63	0.16	-0.25	-1.20	0.227
OS-00048 (7) diurnal (8:00am; day 2; flag leaf) / diurnal (8:00am; day 1; flag leaf)	0.18	-0.25	-1.19	0.186
OS-00121 (30) OsAnt1::AlaAT (3/8) / Nipponbare [ammonium (OsAnt1::AlaAT (3/8); 5...	0.44	-0.25	-1.19	0.018
OS-00020 (2) RSV (WuYun3) / non-infected aeral tissue samples (WuYun3)	0.23	-0.25	-1.19	0.126
OS-00069 (2) PSARK::IPT / Kitaake [drought (PSARK::IPT) / drought (Kitaake)]	0.26	-0.26	-1.19	0.098
OS-00236 (11) BRG 15 / Nayanmoni [arsenate (BRG 15) / arsenate (Nayanmoni)]	0.21	-0.26	-1.20	0.154
OS-00037 (15) kinetin (Zhenshan 97) / untreated seedling samples (Zhenshan 97)	0.12	-0.26	-1.22	0.351
OS-00062 (11) RIL012 / MH63	0.17	-0.26	-1.20	0.227
OS-00055 (9) X. oryzae pv. oryzae (IRBB7; 3hpi) / X. oryzae pv. oryzae (IRBB7; 0hp	0.12	-0.26	-1.21	0.357
OS-00095 (5) IR64 / Nipponbare [X. oryzae pv. oryzae (IR64; MAI1) / X. oryzae pv. oryz	0.46	-0.26	-1.20	0.017
OS-00062 (114) RIL150 / Zhenshan 97	0.36	-0.26	-1.20	0.040
OS-00054 (3) 9804-Rxo1 / 9804 [untreated flag leaf samples (9804-Rxo1) / untreate...	0.75	-0.26	-1.20	0.001
OS-00048 (13) diurnal (12:00am; day 1; flag leaf) / diurnal (8:00am; day 1; flag leaf)	0.22	-0.26	-1.20	0.148
OS-00051 (4) anoxia (N2 for 24h; air for 3h) / anoxia (N2 for 24h)	0.28	-0.26	-1.20	0.083
OS-00221 (14) PL177 / IR64 [salt (PL177; ABA; shoot) / salt (IR64; ABA; shoot)]	0.15	-0.26	-1.20	0.265
OS-00062 (123) RIL159 / MH63	0.18	-0.26	-1.21	0.214
OS-00062 (119) RIL155 / MH63	0.17	-0.27	-1.21	0.234
OS-00062 (21) RIL017 / MH63	0.18	-0.27	-1.21	0.215
OS-00062 (135) RIL165 / MH63	0.17	-0.27	-1.21	0.237
OS-00062 (28) RIL020 / Zhenshan 97	0.22	-0.27	-1.20	0.149
OS-00123 (3) arid / Dongjin [dehydration (arid) / dehydration (Dongjin)]	0.12	-0.27	-1.21	0.350
OS-00225 (5) Dhagaddeshi / IR20 [dehydration (Dhagaddeshi; 3h) / dehydration (IR...	0.27	-0.27	-1.21	0.103
OS-00086 (1) B. graminis f. sp. hordei (24hpi) / non-inoculated leaf samples (24h)	0.19	-0.27	-1.21	0.202
OS-00234 (17) PA64s / 93-11 [optimal fertilization (PA64s; leaf) / optimal fertilization (...	0.13	-0.27	-1.24	0.340
OS-00055 (13) IRBB5 / IR24 [X. oryzae pv. oryzae (IRBB5; 3hpi) / X. oryzae pv. oryzae (	0.51	-0.27	-1.21	0.014
OS-00051 (2) anoxia (N2 for 12h) / anoxia (N2 for 1h)	0.21	-0.28	-1.21	0.176
OS-00062 (98) RIL133 / Zhenshan 97	0.27	-0.28	-1.21	0.107
OS-00118 (2) stamen development (stage 4) / stamen development (stage 2)	0.12	-0.28	-1.24	0.356
OS-00062 (132) RIL163 / Zhenshan 97	0.11	-0.28	-1.17	0.412
OS-00036 (9) phosphorus (no P for 10d; shoot) / iron; phosphorus (no Fe and P for 1...	0.42	-0.28	-1.21	0.032
OS-00074 (1) A. tumefaciens (Nipponbare; 1h) / untreated calli samples (Nipponbare)	0.27	-0.28	-1.21	0.108
OS-00062 (138) RIL169 / Zhenshan 97	0.28	-0.28	-1.21	0.104
OS-00037 (60) Zhenshan 97 / MH63 [untreated stem samples (Zhenshan 97; headi...	0.12	-0.28	-1.21	0.377
OS-00062 (44) RIL053 / Zhenshan 97	0.13	-0.28	-1.19	0.333
OS-00235 (7) drought; heat (Nagina 22; anther; 3x6h) / untreated anther samples (N...	0.15	-0.28	-1.25	0.306
OS-00100 (3) CJF1 / Jamaica [leaf]	0.17	-0.28	-1.23	0.261
OS-00062 (161) RIL197 / MH63	0.17	-0.28	-1.22	0.245
OS-00232 (4) N. lugens (Rathu Heenati; 24hpi) / wounding (Rathu Heenati; 24h)	0.18	-0.28	-1.20	0.224
OS-00062 (116) RIL152 / Zhenshan 97	0.41	-0.28	-1.22	0.037
OS-00062 (88) RIL114 / Zhenshan 97	0.41	-0.29	-1.22	0.036
OS-00055 (10) X. oryzae pv. oryzae (IRBB7; 6hpi) / X. oryzae pv. oryzae (IRBB7; 0hp	0.16	-0.29	-1.24	0.285
OS-00236 (13) BRG 15 / CN1646-5 [arsenate (BRG 15) / arsenate (CN1646-5)]	0.32	-0.29	-1.23	0.079
OS-00070 (4) heat (MH63) / untreated leaf samples (MH63)	0.23	-0.29	-1.23	0.161
OS-00062 (150) RIL185 / Zhenshan 97	0.44	-0.29	-1.23	0.030
OS-00062 (31) RIL026 / MH63	0.19	-0.29	-1.23	0.222
OS-00237 (5) X. oryzae pv. oryzae (Rb49; 4hpi) / mock treated leaf samples (Rb49; 4...	0.16	-0.29	-1.22	0.285
OS-00062 (62) RIL081 / Zhenshan 97	0.46	-0.30	-1.23	0.028
OS-00062 (45) RIL054 / MH63	0.22	-0.30	-1.24	0.176
OS-00062 (179) RIL219 / MH63	0.18	-0.30	-1.23	0.247
OS-00061 (12) X. oryzae pv. oryzae (IR24; PXO99AME7) / mock treated leaf samples ..	0.26	-0.30	-1.23	0.131
OS-00003 (1) arsenate (Azucena) / untreated root samples (Azucena)	0.21	-0.30	-1.23	0.202
OS-00234 (9) LYP9 / 93-11 [optimal fertilization (LYP9; leaf) / optimal fertilization (93-...	0.14	-0.30	-1.25	0.345
OS-00287 (2) salt (5h) / untreated shoot samples (5h)	0.65	-0.30	-1.23	0.007
OS-00062 (168) RIL203 / Zhenshan 97	0.23	-0.30	-1.22	0.169
OS-00055 (7) X. oryzae pv. oryzae (IRBB5; 12hpi) / X. oryzae pv. oryzae (IRBB5; 0hp	0.28	-0.30	-1.25	0.121
OS-00235 (2) drought; heat (Moroberekan; anther; 3x6h) / heat (Moroberekan; anther;...	0.39	-0.30	-1.23	0.050
OS-00062 (20) RIL016 / Zhenshan 97	0.45	-0.30	-1.24	0.033
OS-00286 (4) fenclorim (24h) / mock treated cell culture samples (24h)	0.58	-0.30	-1.24	0.013
OS-00051 (3) anoxia (N2 for 24h) / anoxia (N2 for 1h)	0.29	-0.30	-1.24	0.113
OS-00062 (157) RIL193 / MH63	0.15	-0.31	-1.23	0.315
OS-00035 (21) caryopsis development (top 3/4; 3dap) / caryopsis development (top ...	0.11	-0.31	-1.21	0.446
OS-00062 (58) RIL077 / Zhenshan 97	0.21	-0.31	-1.22	0.203
OS-00062 (129) RIL162 / MH63	0.14	-0.31	-1.22	0.350
OS-00062 (48) RIL059 / Zhenshan 97	0.45	-0.31	-1.24	0.036
OS-00236 (14) BRG 15 / CN1646-5 [untreated root samples (BRG 15) / untreated ro...	0.23	-0.31	-1.24	0.187
OS-00048 (9) diurnal (8:00pm; day 1; flag leaf) / diurnal (8:00am; day 1; flag leaf)	0.25	-0.31	-1.24	0.159
OS-00062 (60) RIL080 / Zhenshan 97	0.39	-0.31	-1.24	0.057
OS-00270 (4) drought (Moroberekan; FTSW 0.5) / untreated leaf elongation zone sa...	0.35	-0.31	-1.24	0.075
OS-00221 (8) salt (PL177; ABA; root) / untreated root samples (PL177)	0.20	-0.31	-1.26	0.229
OS-00062 (96) RIL131 / Zhenshan 97	0.24	-0.31	-1.23	0.168
OS-00270 (1) drought (IR64; FTSW 0.2) / untreated leaf elongation zone samples (IR...	0.48	-0.31	-1.24	0.030
OS-00061 (8) X. oryzae pv. oryzae (IR24; PXO99AME2) / mock treated leaf samples (I...	0.40	-0.32	-1.25	0.056
OS-00018 (8) Nipponbare / IAC 165 [mock treated root samples (Nipponbare; 4dpi) /...	0.35	-0.32	-1.25	0.077
OS-00062 (9) RIL009 / MH63	0.16	-0.32	-1.24	0.313
OS-00102 (4) cold (C418; 24h) / untreated shoot samples (C418)	0.32	-0.32	-1.26	0.103
OS-00018 (4) S. hermonthica (Nipponbare; 2dpi) / mock treated root samples (Nipp...	0.13	-0.32	-1.27	0.385
OS-00095 (1) X. oryzae pv. oryzae (IR64; MAI1) / X. oryzae pv. oryzae (IR64; BA	0.41	-0.32	-1.26	0.055
OS-00037 (58) Zhenshan 97 / MH63 [untreated stem samples (Zhenshan 97; boo...	0.10	-0.33	-1.29	0.492
OS-00061 (14) X. oryzae pv. oryzae (IR24; T7174) / mock treated leaf samples (IR24)	0.45	-0.33	-1.26	0.044
OS-00062 (84) RIL111 / Zhenshan 97	0.46	-0.33	-1.26	0.040
OS-00051 (1) anoxia (N2 for 3h) / anoxia (N2 for 1h)	0.38	-0.33	-1.26	0.074
OS-00062 (182) RIL232 / Zhenshan 97	0.17	-0.33	-1.23	0.300
OS-00055 (14) IRBB5 / IR24 [X. oryzae pv. oryzae (IRBB5; 6hpi) / X. oryzae pv. oryzae (	0.53	-0.33	-1.26	0.026
OS-00037 (17) NAA (Zhenshan 97) / untreated seedling samples (Zhenshan 97)	0.20	-0.34	-1.27	0.262
OS-00062 (113) RIL150 / MH63	0.29	-0.34	-1.27	0.135
OS-00233 (14) Douradoagulha / Khaumeo [untreated panicle samples (Douradoag...	0.43	-0.34	-1.27	0.055
OS-00062 (36) RIL039 / Zhenshan 97	0.38	-0.34	-1.26	0.080
OS-00062 (27) RIL020 / MH63	0.25	-0.34	-1.27	0.188
OS-00041 (3) drought (leaf; tillering) / untreated leaf samples (tillering)	0.21	-0.35	-1.25	0.240
OS-00062 (2) RIL004 / Zhenshan 97	0.43	-0.35	-1.27	0.056
OS-00062 (112) RIL149 / Zhenshan 97	0.61	-0.35	-1.27	0.018
OS-00062 (174) RIL209 / Zhenshan 97	0.42	-0.35	-1.27	0.063
OS-00062 (6) RIL006 / Zhenshan 97	0.37	-0.35	-1.27	0.088
OS-00062 (97) RIL133 / MH63	0.28	-0.35	-1.28	0.162
OS-00062 (131) RIL163 / MH63	0.16	-0.35	-1.24	0.360
OS-00037 (24) panicle development (Zhenshan 97; 40-50mm) / panicle developme...	0.08	-0.35	-1.43	0.596
OS-00100 (6) Jamaica / C Bao [root]	0.21	-0.35	-1.27	0.253
OS-00062 (126) RIL160 / Zhenshan 97	0.58	-0.35	-1.28	0.023
OS-00062 (8) RIL007 / Zhenshan 97	0.57	-0.36	-1.28	0.026
OS-00062 (76) RIL101 / Zhenshan 97	0.41	-0.36	-1.28	0.071
OS-00062 (137) RIL169 / MH63	0.29	-0.36	-1.29	0.158
OS-00062 (43) RIL053 / MH63	0.19	-0.36	-1.26	0.300
OS-00121 (24) OsAnt1::AlaAT (1/7) / Nipponbare [ammonium (OsAnt1::AlaAT (1/7); 5...	0.57	-0.36	-1.28	0.025
OS-00062 (115) RIL152 / MH63	0.33	-0.36	-1.29	0.120
OS-00062 (87) RIL114 / MH63	0.33	-0.36	-1.29	0.120
OS-00062 (54) RIL071 / Zhenshan 97	0.34	-0.36	-1.28	0.120
OS-00121 (4) ammonium (Nipponbare; 0.5mM (NH4)2SO4; shoot) / ammonium (Ni...	0.51	-0.36	-1.29	0.040
OS-00103 (8) cold (LTH; 4°C for 8h) / untreated leaf samples (LTH)	0.51	-0.36	-1.29	0.039
OS-00235 (5) drought; heat (Nagina 22; anther; 1x6h) / untreated anther samples (N...	0.23	-0.37	-1.32	0.241
OS-00062 (64) RIL082 / Zhenshan 97	0.58	-0.37	-1.29	0.026
OS-00103 (9) cold (LTH; 4°C for 24h) / untreated leaf samples (LTH)	0.46	-0.37	-1.29	0.055
OS-00062 (148) RIL184 / Zhenshan 97	0.58	-0.37	-1.29	0.027
OS-00062 (149) RIL185 / MH63	0.35	-0.37	-1.30	0.113
OS-00074 (2) A. tumefaciens (Nipponbare; 6h) / untreated calli samples (Nipponbare)	0.36	-0.37	-1.27	0.102
OS-00062 (82) RIL106 / Zhenshan 97	0.57	-0.37	-1.29	0.029
OS-00062 (61) RIL081 / MH63	0.36	-0.37	-1.30	0.110
OS-00105 (1) X. oryzae pv. oryzae (IR24; 24hpi) / mock treated leaf samples (IR24; 2...	0.28	-0.37	-1.31	0.176
OS-00062 (38) RIL042 / Zhenshan 97	0.34	-0.37	-1.28	0.126
OS-00108 (9) imbibition (24h; embryo) / embryo samples from dry seeds	0.27	-0.37	-1.28	0.184
OS-00121 (3) ammonium (Nipponbare; 0.5mM (NH4)2SO4; shoot) / ammonium (Ni...	0.19	-0.37	-1.33	0.305
OS-00062 (102) RIL137 / Zhenshan 97	0.68	-0.37	-1.30	0.015
OS-00062 (167) RIL203 / MH63	0.27	-0.38	-1.29	0.190
OS-00286 (1) CMP (4h) / mock treated cell culture samples (4h)	1.04	-0.38	-1.30	0.002
OS-00116 (3) seed deterioration (45°C) / seed deterioration (40°C)	0.50	-0.38	-1.29	0.048
OS-00080 (1) M. incognita (6dpi) / uninfected root samples	0.31	-0.38	-1.28	0.151
OS-00062 (19) RIL016 / MH63	0.37	-0.38	-1.31	0.109
OS-00062 (57) RIL077 / MH63	0.26	-0.38	-1.30	0.208
OS-00062 (40) RIL047 / Zhenshan 97	0.53	-0.39	-1.30	0.042
OS-00062 (47) RIL059 / MH63	0.38	-0.39	-1.32	0.108
OS-00048 (17) diurnal (0:00am; day 2; flag leaf) / diurnal (8:00am; day 1; flag leaf)	0.44	-0.39	-1.31	0.075
OS-00062 (59) RIL080 / MH63	0.36	-0.39	-1.32	0.120
OS-00062 (95) RIL131 / MH63	0.29	-0.39	-1.31	0.185
OS-00118 (3) stamen development (stage 5) / stamen development (stage 2)	0.23	-0.39	-1.34	0.266
OS-00055 (6) X. oryzae pv. oryzae (IRBB5; 6hpi) / X. oryzae pv. oryzae (IRBB5; 0hp	0.42	-0.40	-1.33	0.086
OS-00018 (2) S. hermonthica (IAC 165; 4dpi) / mock treated root samples (IAC 165; ...	0.50	-0.40	-1.32	0.055
OS-00062 (104) RIL140 / Zhenshan 97	0.59	-0.40	-1.32	0.035
OS-00062 (83) RIL111 / MH63	0.40	-0.41	-1.33	0.104
OS-00062 (18) RIL015 / Zhenshan 97	0.55	-0.41	-1.32	0.044
OS-00234 (6) optimal fertilization (PA64s; root tip) / nutrient deficiency (PA64s; root tip)	0.58	-0.41	-1.32	0.038
OS-00018 (10) Nipponbare / IAC 165 [S. hermonthica (Nipponbare; 2dpi) / S. hermo...	0.42	-0.41	-1.32	0.094
OS-00062 (181) RIL232 / MH63	0.23	-0.41	-1.30	0.272
OS-00062 (128) RIL161 / Zhenshan 97	0.50	-0.41	-1.32	0.060
OS-00061 (1) X. oryzae pv. oryzae (IR24; PXO86) / mock treated leaf samples (IR24)	0.48	-0.41	-1.33	0.067
OS-00074 (8) A. tumefaciens (Zhenshan 97; 24h) / untreated calli samples (Zhensh...	0.23	-0.41	-1.35	0.271
OS-00235 (10) heat (Nagina 22; anther; 3x6h) / untreated anther samples (Nagina 22)	0.31	-0.42	-1.37	0.181
OS-00062 (35) RIL039 / MH63	0.38	-0.42	-1.34	0.124
OS-00061 (4) X. oryzae pv. oryzae (IR24; PXO99A) / mock treated leaf samples (IR24)	0.53	-0.42	-1.34	0.054
OS-00062 (108) RIL145 / Zhenshan 97	0.69	-0.42	-1.34	0.023
OS-00062 (1) RIL004 / MH63	0.41	-0.42	-1.35	0.109
OS-00062 (160) RIL195 / Zhenshan 97	0.80	-0.42	-1.34	0.013
OS-00062 (111) RIL149 / MH63	0.46	-0.42	-1.35	0.082
OS-00062 (173) RIL209 / MH63	0.40	-0.42	-1.35	0.113
OS-00062 (5) RIL006 / MH63	0.38	-0.42	-1.34	0.128
OS-00018 (6) S. hermonthica (Nipponbare; 11dpi) / mock treated root samples (Nip...	0.72	-0.43	-1.34	0.020
OS-00035 (4) anther development (microspores) / anther development (diplotene-tet...	1.25	-0.43	-1.35	0.001
OS-00062 (125) RIL160 / MH63	0.46	-0.43	-1.36	0.084
OS-00108 (3) imbibition (8h; embryo) / embryo samples from dry seeds	0.62	-0.43	-1.35	0.036
OS-00062 (7) RIL007 / MH63	0.46	-0.43	-1.36	0.086
OS-00054 (1) X. oryzae pv. oryzicola (9804) / untreated flag leaf samples (9804)	0.65	-0.43	-1.34	0.031
OS-00062 (75) RIL101 / MH63	0.41	-0.43	-1.35	0.115
OS-00061 (10) X. oryzae pv. oryzae (IR24; PXO99AME5) / mock treated leaf samples ..	0.44	-0.43	-1.34	0.095
OS-00062 (176) RIL210 / Zhenshan 97	0.34	-0.43	-1.32	0.169
OS-00112 (1) OsCGA1-OX / Kaybonnet	0.59	-0.44	-1.37	0.043
OS-00037 (44) Zhenshan 97 / MH63 [panicle development (Zhenshan 97; 40-50mm...	0.79	-0.44	-1.36	0.016
OS-00062 (53) RIL071 / MH63	0.37	-0.44	-1.35	0.144
OS-00035 (16) caryopsis development (top 3/4; 1dap) / caryopsis development (bott...	0.43	-0.44	-1.37	0.108
OS-00062 (63) RIL082 / MH63	0.48	-0.44	-1.37	0.083
OS-00062 (144) RIL180 / Zhenshan 97	0.81	-0.44	-1.36	0.015
OS-00062 (147) RIL184 / MH63	0.48	-0.44	-1.37	0.083
OS-00035 (17) caryopsis development (top 3/4; 1dap) / ovary development (top 3/4; 0...	0.22	-0.44	-1.41	0.319
OS-00062 (81) RIL106 / MH63	0.48	-0.44	-1.37	0.084
OS-00062 (170) RIL205 / Zhenshan 97	0.75	-0.45	-1.36	0.021
OS-00062 (37) RIL042 / MH63	0.37	-0.45	-1.36	0.147
OS-00062 (101) RIL137 / MH63	0.51	-0.45	-1.38	0.073
OS-00221 (11) salt (PL177; root) / untreated root samples (PL177)	0.48	-0.45	-1.39	0.086
OS-00062 (56) RIL072 / Zhenshan 97	0.72	-0.45	-1.37	0.026
OS-00062 (106) RIL141 / Zhenshan 97	0.85	-0.45	-1.37	0.014
OS-00062 (94) RIL129 / Zhenshan 97	0.82	-0.46	-1.37	0.016
OS-00062 (39) RIL047 / MH63	0.48	-0.46	-1.38	0.089
OS-00283 (7) crl1 / Taichung 65 [untreated crown samples (crl1) / untreated crown s...	0.64	-0.47	-1.39	0.044
OS-00061 (6) X. oryzae pv. oryzae (IR24; PXO99AME1) / mock treated leaf samples (I..	0.72	-0.48	-1.39	0.031
OS-00062 (103) RIL140 / MH63	0.53	-0.48	-1.40	0.080
OS-00062 (17) RIL015 / MH63	0.51	-0.48	-1.40	0.086
OS-00062 (122) RIL156 / Zhenshan 97	0.63	-0.48	-1.39	0.050
OS-00062 (127) RIL161 / MH63	0.49	-0.48	-1.40	0.096
OS-00035 (11) callus regeneration (8d) / callus growth	1.26	-0.48	-1.40	0.003
OS-00062 (50) RIL066 / Zhenshan 97	0.52	-0.49	-1.38	0.086
OS-00062 (74) RIL099 / Zhenshan 97	0.75	-0.49	-1.40	0.029
OS-00116 (2) seed deterioration (40°C) / embryo samples from mock treated dry se...	0.93	-0.49	-1.41	0.013
OS-00062 (107) RIL145 / MH63	0.58	-0.50	-1.42	0.068
OS-00062 (159) RIL195 / MH63	0.61	-0.50	-1.42	0.059
OS-00062 (146) RIL183 / Zhenshan 97	0.92	-0.51	-1.42	0.015
OS-00055 (11) X. oryzae pv. oryzae (IRBB7; 12hpi) / X. oryzae pv. oryzae (IRBB7; 0hp	0.57	-0.51	-1.45	0.075
OS-00062 (175) RIL210 / MH63	0.39	-0.51	-1.40	0.169
OS-00061 (31) X. oryzae pv. oryzicola (IR24) / mock treated leaf samples (IR24)	0.96	-0.52	-1.44	0.014
OS-00062 (143) RIL180 / MH63	0.65	-0.52	-1.44	0.057
OS-00062 (169) RIL205 / MH63	0.63	-0.52	-1.44	0.061
OS-00061 (5) X. oryzae pv. oryzae (IR24; PXO99A) / mock treated leaf samples (IR24)	0.83	-0.52	-1.44	0.026
OS-00062 (55) RIL072 / MH63	0.63	-0.53	-1.45	0.064
OS-00062 (105) RIL141 / MH63	0.67	-0.53	-1.45	0.054
OS-00062 (93) RIL129 / MH63	0.67	-0.53	-1.46	0.055
OS-00035 (10) callus regeneration (6d) / callus growth	0.83	-0.53	-1.43	0.027
OS-00062 (152) RIL186 / Zhenshan 97	1.00	-0.54	-1.45	0.013
OS-00062 (164) RIL198 / Zhenshan 97	0.65	-0.54	-1.44	0.062
OS-00062 (121) RIL156 / MH63	0.62	-0.56	-1.47	0.078
OS-00062 (49) RIL066 / MH63	0.55	-0.56	-1.47	0.104
OS-00062 (73) RIL099 / MH63	0.69	-0.56	-1.48	0.061
OS-00062 (24) RIL018 / Zhenshan 97	0.87	-0.57	-1.48	0.029
OS-00062 (145) RIL183 / MH63	0.77	-0.58	-1.50	0.047
OS-00062 (86) RIL112 / Zhenshan 97	1.06	-0.58	-1.50	0.015
OS-00062 (151) RIL186 / MH63	0.84	-0.61	-1.54	0.042
OS-00062 (163) RIL198 / MH63	0.68	-0.62	-1.53	0.081
OS-00062 (172) RIL206 / Zhenshan 97	1.02	-0.63	-1.54	0.024
OS-00037 (4) callus regeneration (Zhenshan 97; 5d) / callus growth (Zhenshan 97; ...	1.24	-0.64	-1.56	0.011
OS-00062 (23) RIL018 / MH63	0.83	-0.64	-1.56	0.052
OS-00074 (5) A. tumefaciens (Zhenshan 97; 1h) / untreated calli samples (Zhensha...	0.53	-0.65	-1.56	0.149
OS-00062 (85) RIL112 / MH63	0.93	-0.66	-1.59	0.039
OS-00037 (11) endosperm development (Zhenshan 97; 14dap) / endosperm develo...	0.94	-0.70	-1.62	0.046
OS-00062 (171) RIL206 / MH63	0.96	-0.71	-1.63	0.043
OS-00284 (2) 35S-15 / Wuxiangjing 9	0.36	-0.74	-1.96	0.320
OS-00074 (7) A. tumefaciens (Zhenshan 97; 12h) / untreated calli samples (Zhensh...	0.92	-0.82	-1.78	0.076
OS-00035 (9) callus regeneration (4d) / callus growth	3.34	-0.95	-1.93	<0.001
OS-00035 (8) callus regeneration (2d) / callus growth	1.87	-0.97	-1.91	0.012
OS-00074 (13) Zhenshan 97 / Nipponbare [untreated calli samples (Zhenshan 97) / ...	1.70	-0.99	-1.92	0.019
OS-00074 (12) Zhenshan 97 / Nipponbare [A. tumefaciens (Zhenshan 97; 24h) / A. tu...	2.42	-1.32	-2.45	0.015
OS-00074 (9) Zhenshan 97 / Nipponbare [A. tumefaciens (Zhenshan 97; 1h) / A. tum...	2.77	-1.35	-2.49	0.009
OS-00088 (2) embryo development (12daa) / embryo development (9daa)	1.26	-1.39	-2.78	0.123
OS-00035 (20) caryopsis development (top 3/4; 3dap) / caryopsis development (bott...	2.47	-1.41	-2.63	0.018
OS-00279 (1) fst (cyn) / Junambyeo [untreated panicle samples (fst (cyn); 5dap) / unt...	1.97	-1.46	-3.03	0.045
OS-00088 (3) endosperm development (9daa) / endosperm development (3daa)	1.27	-1.59	-3.27	0.159
OS-00037 (47) Zhenshan 97 / MH63 [untreated calli samples (Zhenshan 97; 26°C; 1...	0.47	-1.72	-1.48	0.531
OS-00035 (18) caryopsis development (top 3/4; 2dap) / caryopsis development (bott...	4.54	-1.75	-3.36	0.003
OS-00074 (11) Zhenshan 97 / Nipponbare [A. tumefaciens (Zhenshan 97; 12h) / A. tu...	5.01	-1.80	-3.38	0.002
OS-00035 (22) caryopsis development (top 3/4; 4dap) / caryopsis development (bott...	3.64	-1.90	-4.07	0.012
OS-00074 (6) A. tumefaciens (Zhenshan 97; 6h) / untreated calli samples (Zhensha...	3.94	-2.38	-4.47	0.022
OS-00088 (1) embryo development (9daa) / embryo development (3daa)	3.00	-2.42	-5.13	0.057
OS-00074 (10) Zhenshan 97 / Nipponbare [A. tumefaciens (Zhenshan 97; 6h) / A. tu...	6.18	-3.00	-6.75	0.009

created with GENEVESTIGATOR
